# Publisher Correction: ELI trifocal microscope: a precise system to prepare target cryo-lamellae for in situ cryo-ET study

**DOI:** 10.1038/s41592-023-01867-2

**Published:** 2023-03-31

**Authors:** Shuoguo Li, Ziyan Wang, Xing Jia, Tongxin Niu, Jianguo Zhang, Guoliang Yin, Xiaoyun Zhang, Yun Zhu, Gang Ji, Fei Sun

**Affiliations:** 1 Center for Biological Imaging, Core Facilities for Protein Science, Institute of Biophysics, Chinese Academy of Sciences, Beijing, China; 2 University of Chinese Academy of Sciences, Beijing, China; 3 National Key Laboratory of Biomacromolecules, CAS Center for Excellence in Biomacromolecules, Institute of Biophysics, Chinese Academy of Sciences, Beijing, China

**Keywords:** Molecular biophysics, Cryoelectron microscopy, Cryoelectron tomography

Correction to: *Nature Methods* 10.1038/s41592-022-01748-0. Published online 16 January 2022.

In the version of this article originally published, Shuoguo Li and Ziyan Wang were missing descriptors as equally contributing authors. The error has been corrected in the HTML and PDF versions of the article.

