# Supplementary material for: ELI trifocal microscope: a precise system to prepare target cryo-lamellae for in situ cryo-ET study
Source: Nat Methods. 2023 Jan 16;20(2):276–83. doi: 10.1038/s41592-022-01748-0 (PMC9911351; doi:10.1038/s41592-022-01748-0)
Supplement: Supplementary file 1 — Supplementary Fig. 1 [file 41592_2022_1748_MOESM1_ESM.pdf]

nature methods

Article

https://doi.org/10.1038/s41592-022-01748-0

# ELI trifocal microscope: a precise system to prepare target cryo-lamellae for in situ cryo-ET study

In the format provided by the authors and unedited

# Supplementary information for “ELI trifocal microscope: A precise cryogenic fabrication system to prepare target cryo-lamellae of cells for in situ cryo-ET study”

**Supplementary Figure 1. The list of cryo-EM images of all the cryo-lamellae fabricated in this study.**

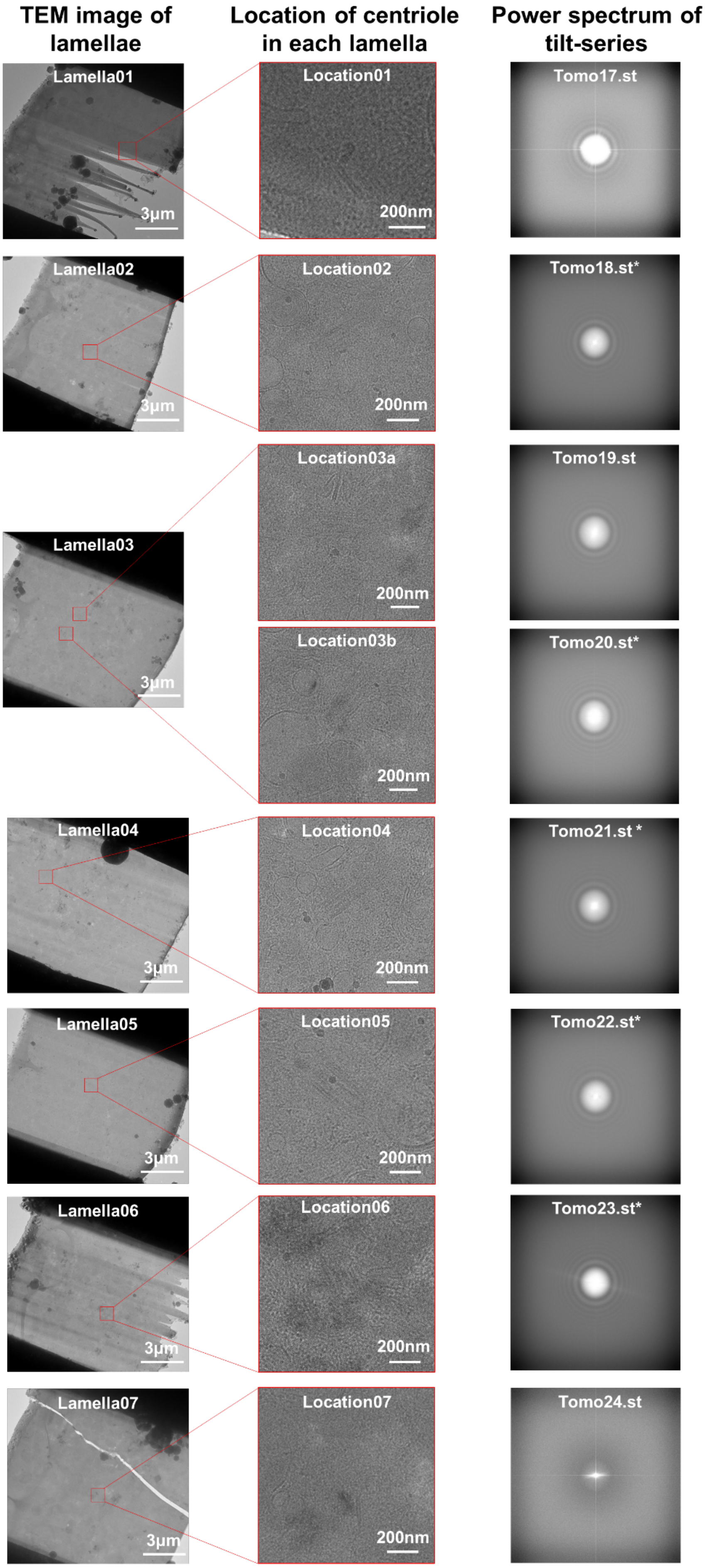
TEM image of lamellae
Location of centriole in each lamella
Power spectrum of tilt-series
Lamella01
Location01
Tomo17.st
3µm
200nm
Lamella02
Location02
Tomo18.st*
3µm
200nm
Lamella03
Location03a
Tomo19.st
Location03b
Tomo20.st*
3µm
200nm
200nm
Lamella04
Location04
Tomo21.st *
3µm
200nm
Lamella05
Location05
Tomo22.st*
3µm
200nm
Lamella06
Location06
Tomo23.st*
3µm
200nm
Lamella07
Location07
Tomo24.st
3µm
200nm

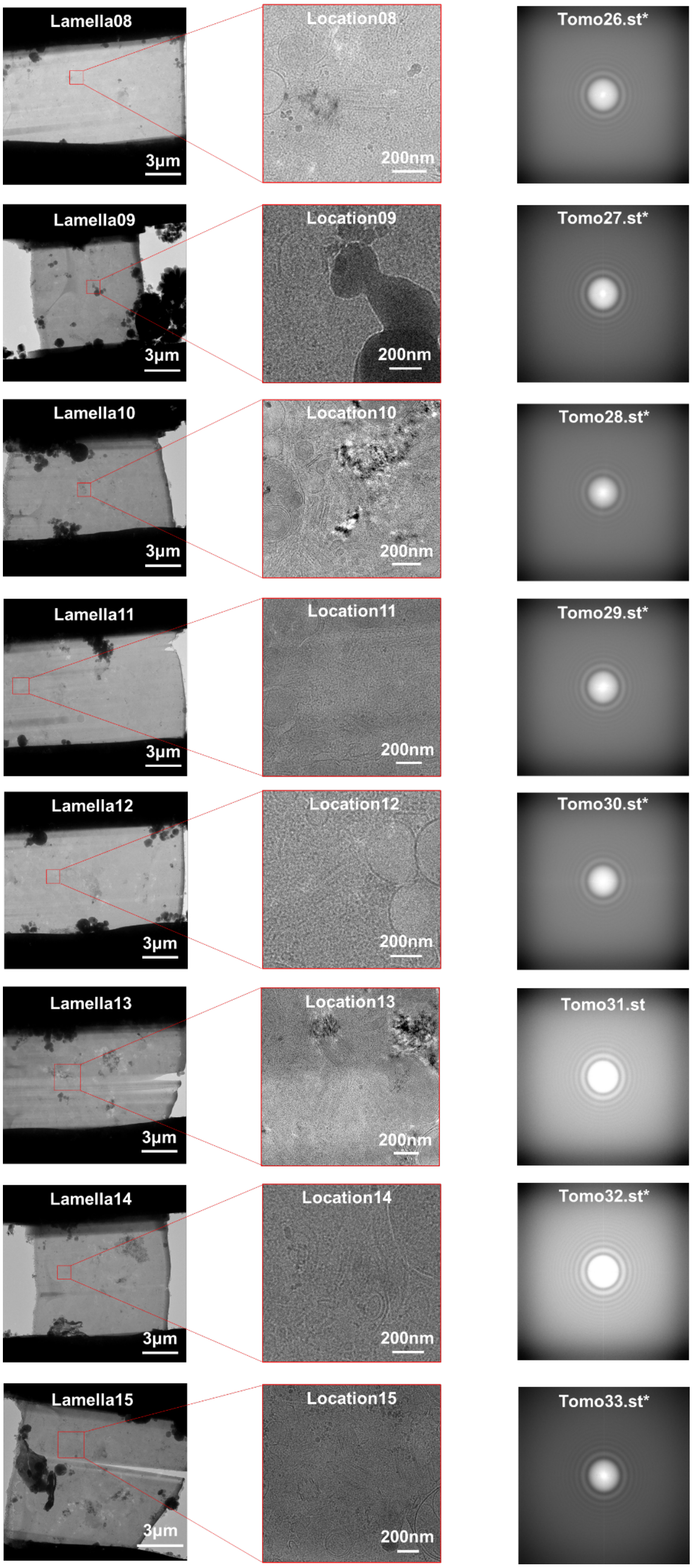

Lamella08
3μm
Location08
200nm
Tomo26.st*
Lamella09
3μm
Location09
200nm
Tomo27.st*
Lamella10
3μm
Location10
200nm
Tomo28.st*
Lamella11
3μm
Location11
200nm
Tomo29.st*
Lamella12
3μm
Location12
200nm
Tomo30.st*
Lamella13
3μm
Location13
200nm
Tomo31.st
Lamella14
3μm
Location14
200nm
Tomo32.st*
Lamella15
3μm
Location15
200nm
Tomo33.st*

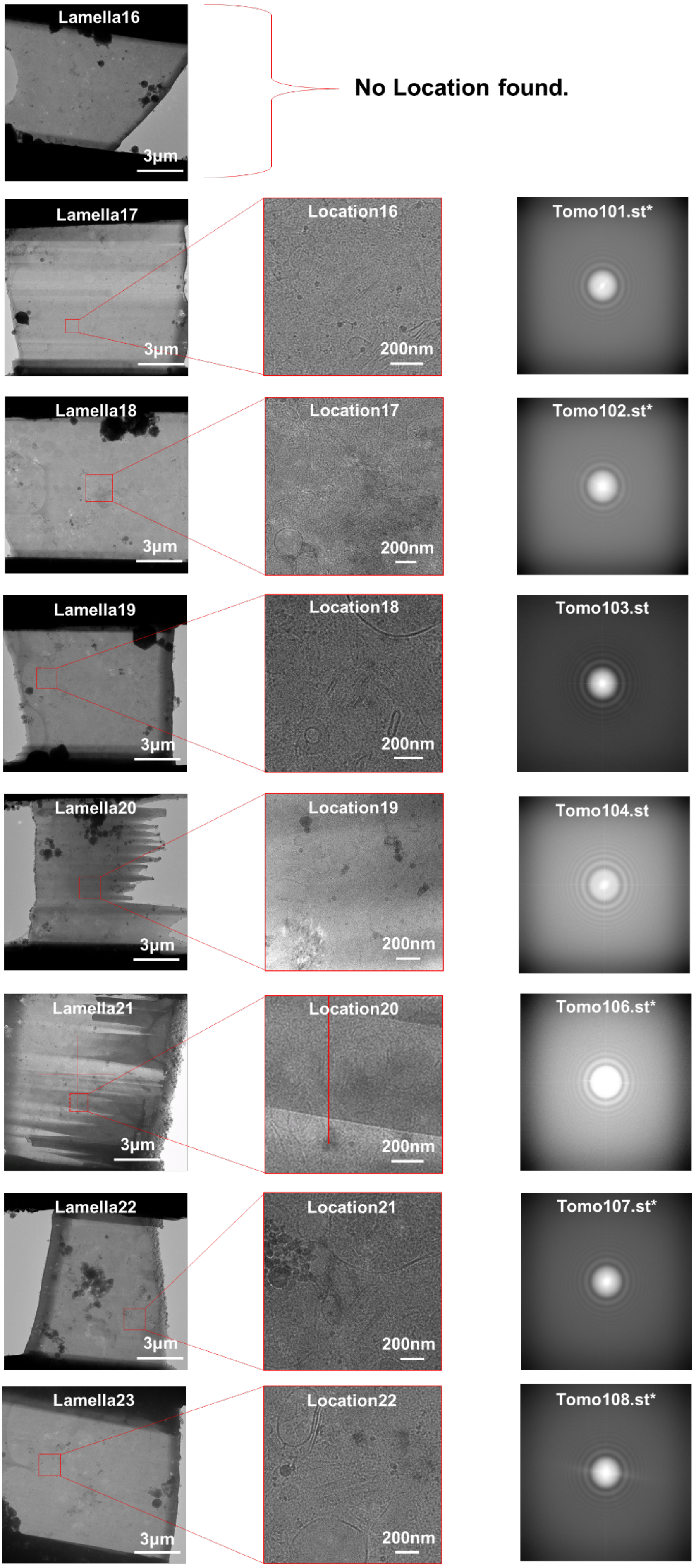
Lamella16
3μm
No Location found.
Lamella17
3μm
Location16
200nm
Tomo101.st*
Lamella18
3μm
Location17
200nm
Tomo102.st*
Lamella19
3μm
Location18
200nm
Tomo103.st
Lamella20
3μm
Location19
200nm
Tomo104.st
Lamella21
3μm
Location20
200nm
Tomo106.st*
Lamella22
3μm
Location21
200nm
Tomo107.st*
Lamella23
3μm
Location22
200nm
Tomo108.st*

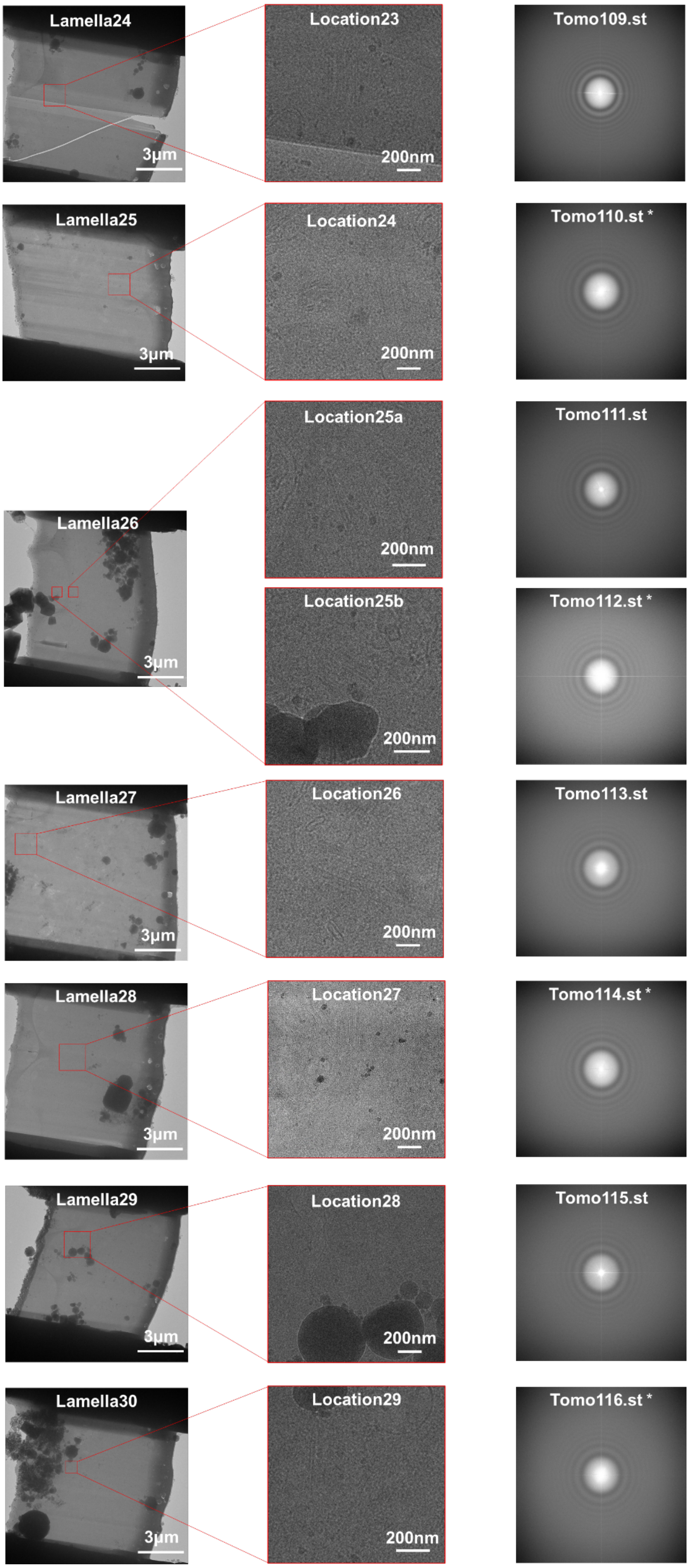
Lamella24
3μm
Location23
200nm
Tomo109.st
Lamella25
3μm
Location24
200nm
Tomo110.st *
Location25a
200nm
Tomo111.st
Lamella26
3μm
Location25b
200nm
Tomo112.st *
Lamella27
3μm
Location26
200nm
Tomo113.st
Lamella28
3μm
Location27
200nm
Tomo114.st *
Lamella29
3μm
Location28
200nm
Tomo115.st
Lamella30
3μm
Location29
200nm
Tomo116.st *

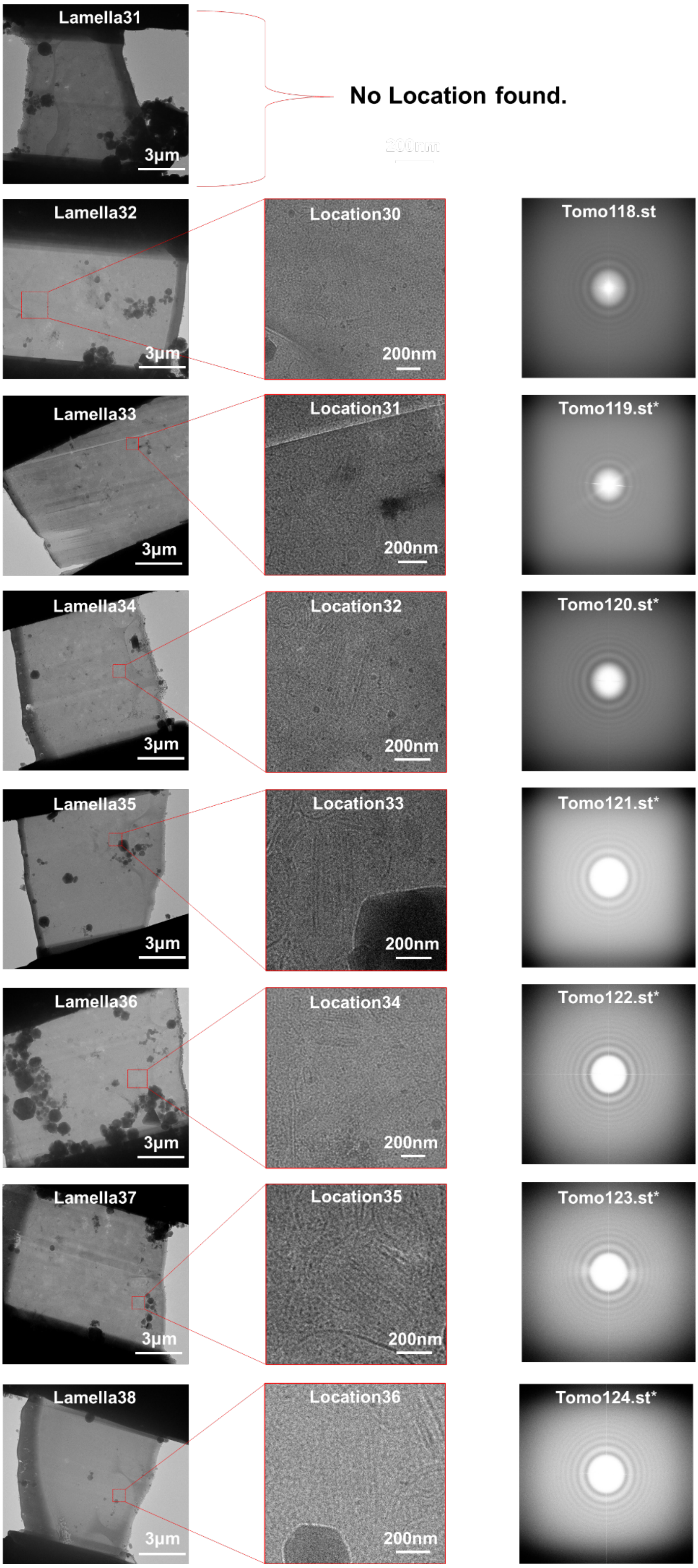

Lamella31
3μm
No Location found.
Lamella32
3μm
Location30
200nm
Tomo118.st
Lamella33
3μm
Location31
200nm
Tomo119.st*
Lamella34
3μm
Location32
200nm
Tomo120.st*
Lamella35
3μm
Location33
200nm
Tomo121.st*
Lamella36
3μm
Location34
200nm
Tomo122.st*
Lamella37
3μm
Location35
200nm
Tomo123.st*
Lamella38
3μm
Location36
200nm
Tomo124.st*

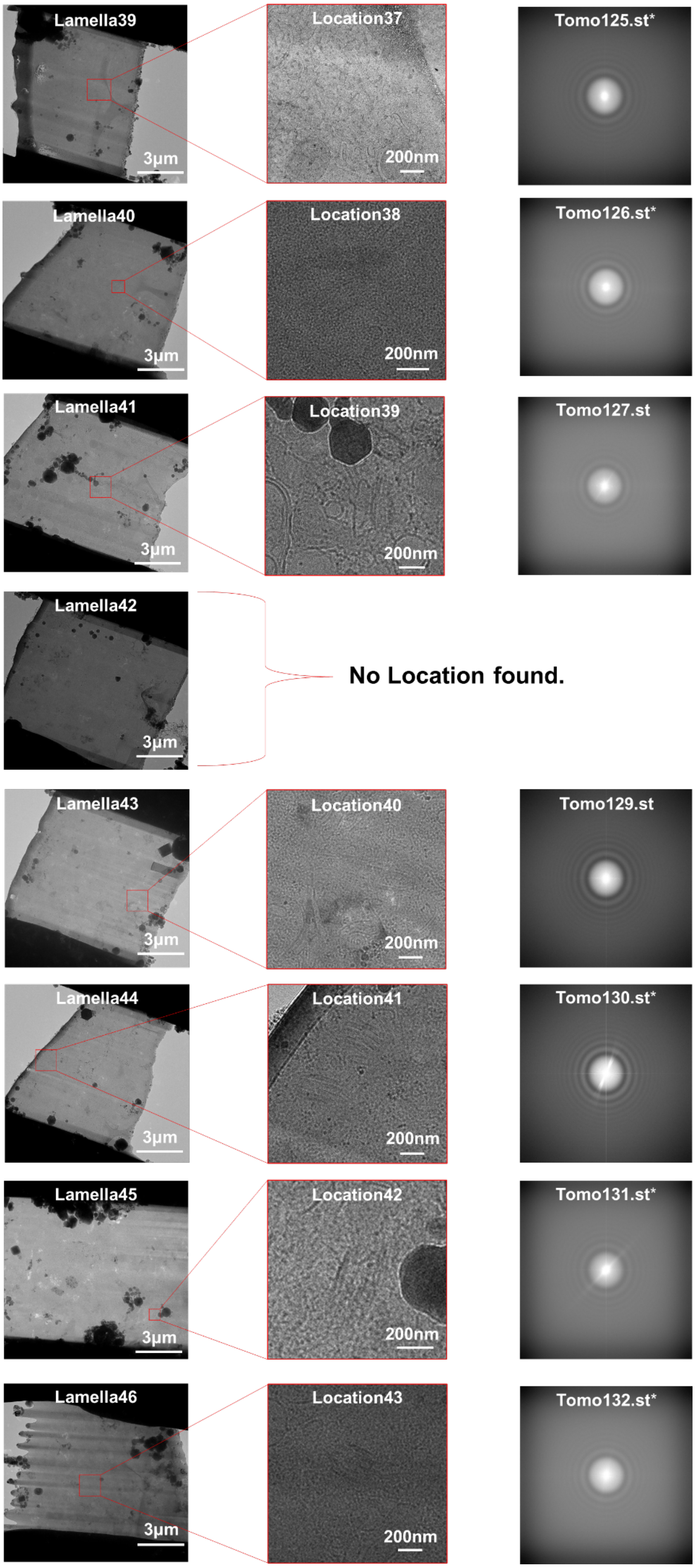
Lamella39
3μm
Location37
200nm
Tomo125.st*
Lamella40
3μm
Location38
200nm
Tomo126.st*
Lamella41
3μm
Location39
200nm
Tomo127.st
Lamella42
3μm
No Location found.
Lamella43
3μm
Location40
200nm
Tomo129.st
Lamella44
3μm
Location41
200nm
Tomo130.st*
Lamella45
3μm
Location42
200nm
Tomo131.st*
Lamella46
3μm
Location43
200nm
Tomo132.st*

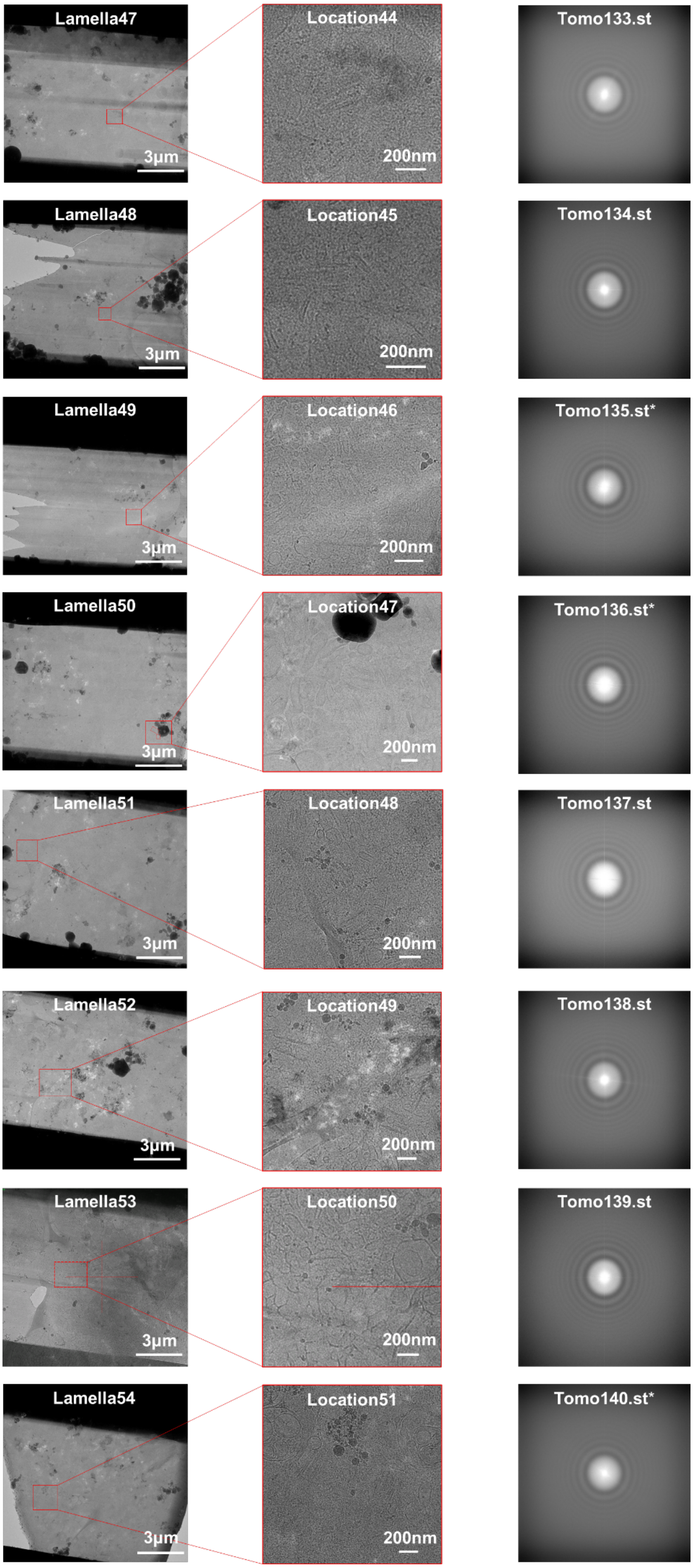
Lamella47
3μm
Location44
200nm
Tomo133.st
Lamella48
3μm
Location45
200nm
Tomo134.st
Lamella49
3μm
Location46
200nm
Tomo135.st*
Lamella50
3μm
Location47
200nm
Tomo136.st*
Lamella51
3μm
Location48
200nm
Tomo137.st
Lamella52
3μm
Location49
200nm
Tomo138.st
Lamella53
3μm
Location50
200nm
Tomo139.st
Lamella54
3μm
Location51
200nm
Tomo140.st*

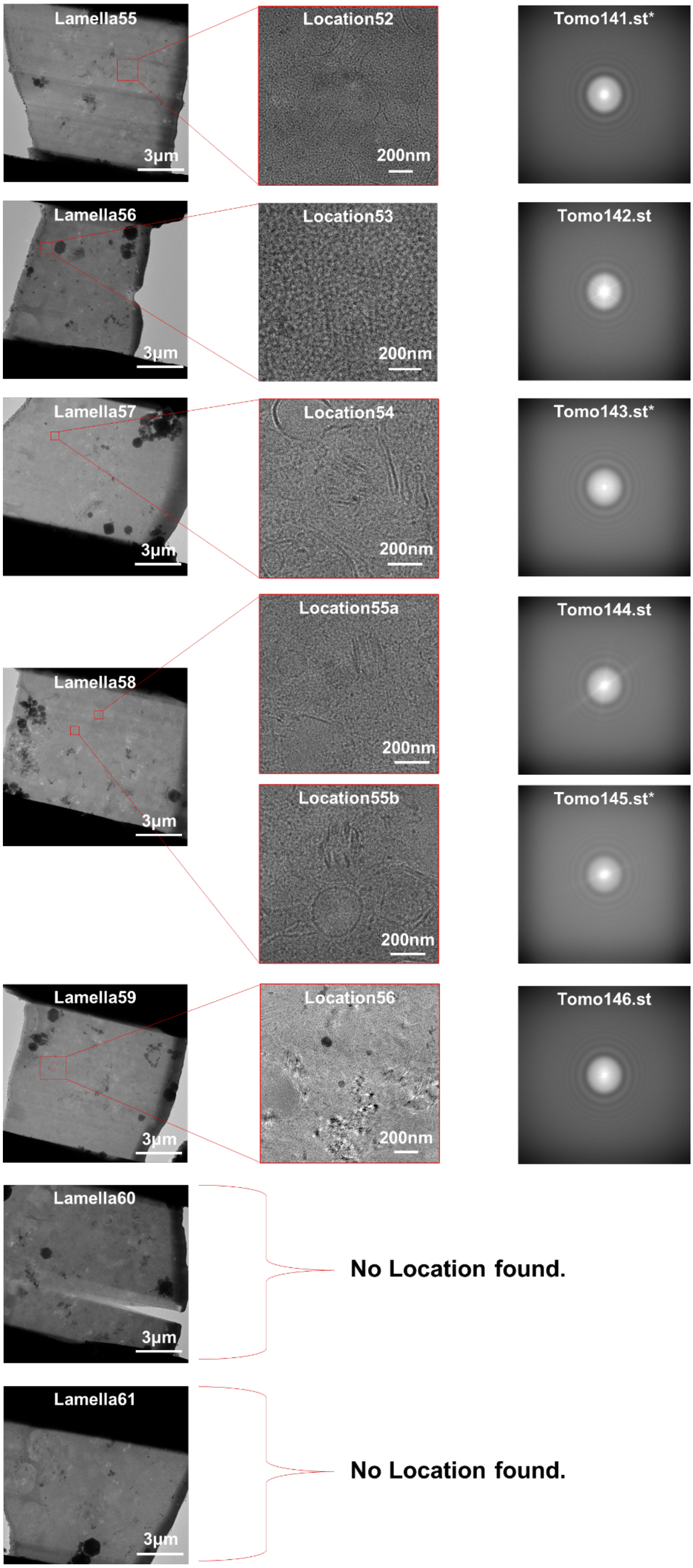

Lamella55
3μm
Location52
200nm
Tomo141.st*
Lamella56
3μm
Location53
200nm
Tomo142.st
Lamella57
3μm
Location54
200nm
Tomo143.st*
Lamella58
3μm
Location55a
200nm
Tomo144.st
Location55b
200nm
Tomo145.st*
Lamella59
3μm
Location56
200nm
Tomo146.st
Lamella60
3μm
No Location found.
Lamella61
3μm
No Location found.

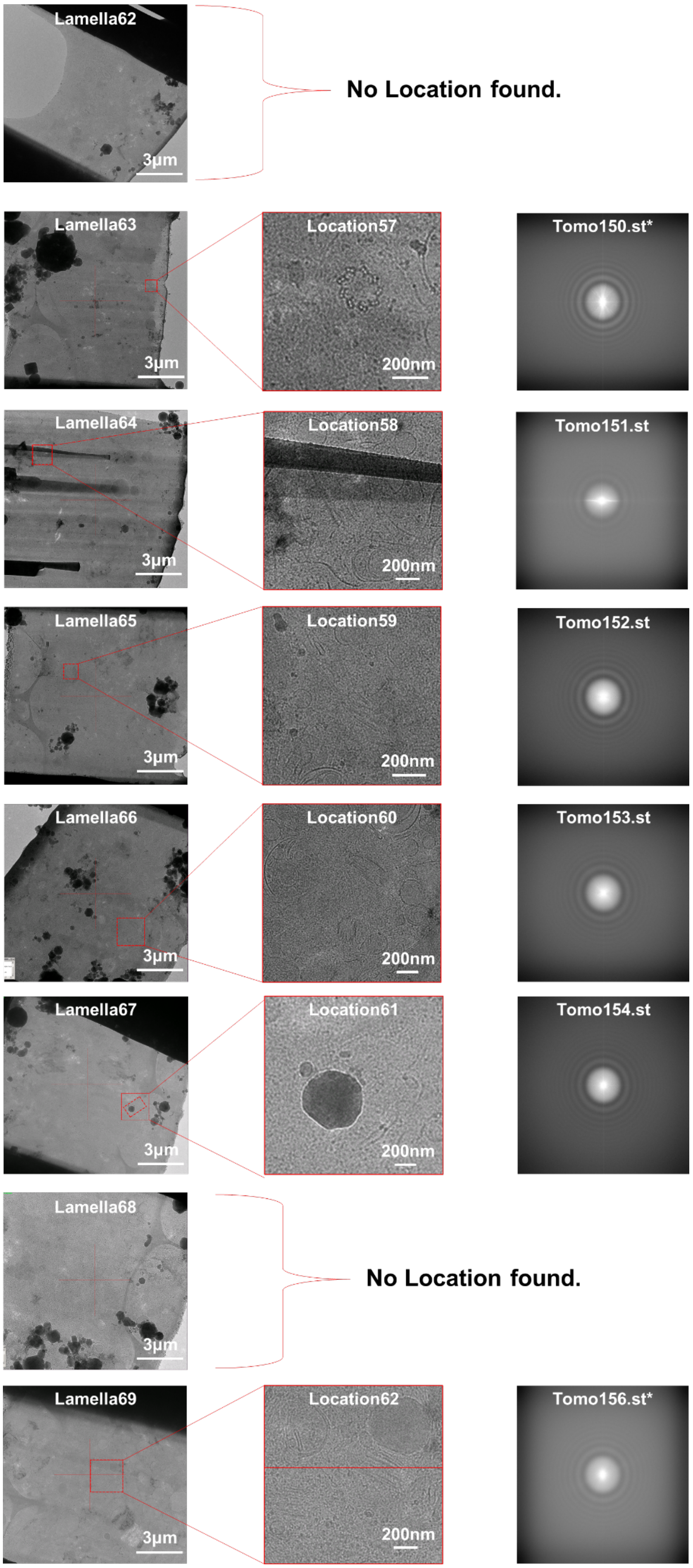
Lamella62
3μm
No Location found.
Lamella63
3μm
Location57
200nm
Tomo150.st*
Lamella64
3μm
Location58
200nm
Tomo151.st
Lamella65
3μm
Location59
200nm
Tomo152.st
Lamella66
3μm
Location60
200nm
Tomo153.st
Lamella67
3μm
Location61
200nm
Tomo154.st
Lamella68
3μm
No Location found.
Lamella69
3μm
Location62
200nm
Tomo156.st*

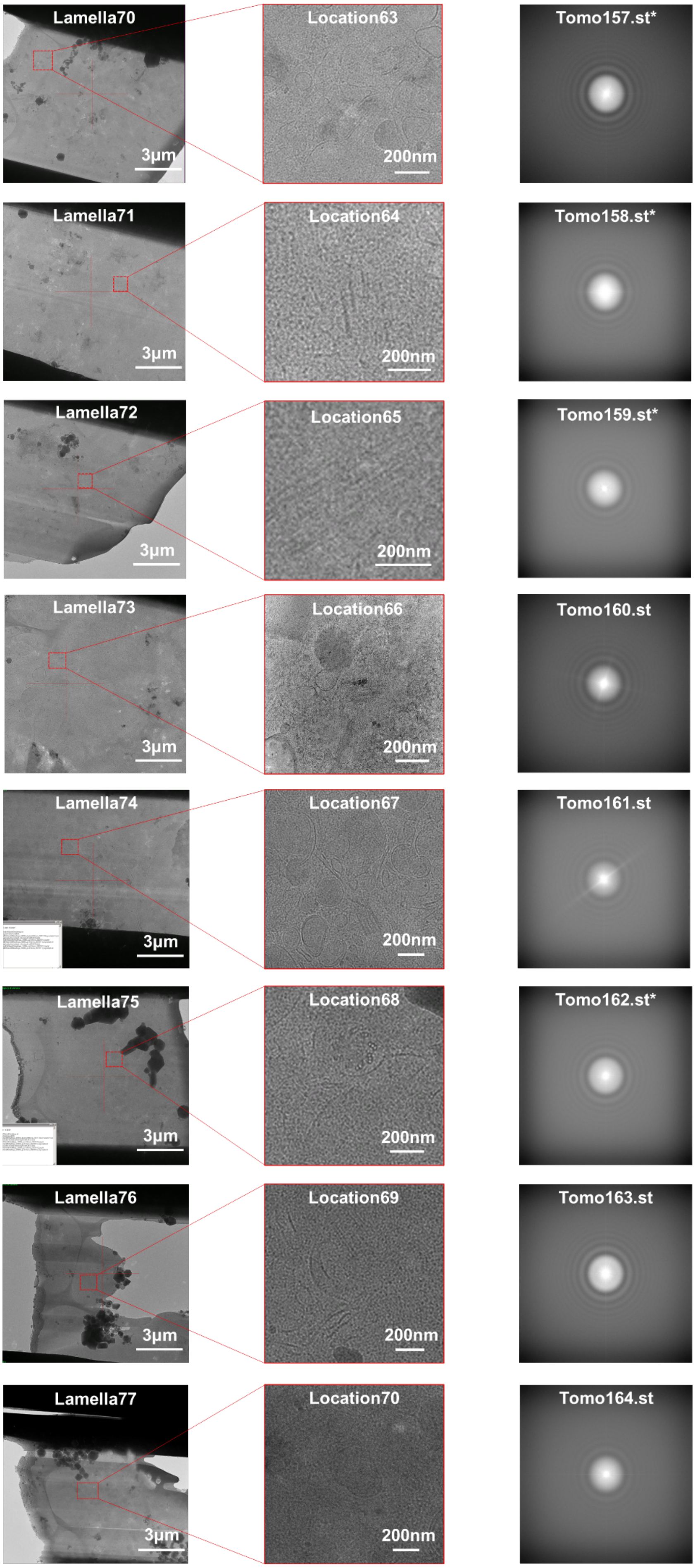
Lamella70
3µm
Location63
200nm
Tomo157.st*
Lamella71
3µm
Location64
200nm
Tomo158.st*
Lamella72
3µm
Location65
200nm
Tomo159.st*
Lamella73
3µm
Location66
200nm
Tomo160.st
Lamella74
3µm
Location67
200nm
Tomo161.st
Lamella75
3µm
Location68
200nm
Tomo162.st*
Lamella76
3µm
Location69
200nm
Tomo163.st
Lamella77
3µm
Location70
200nm
Tomo164.st

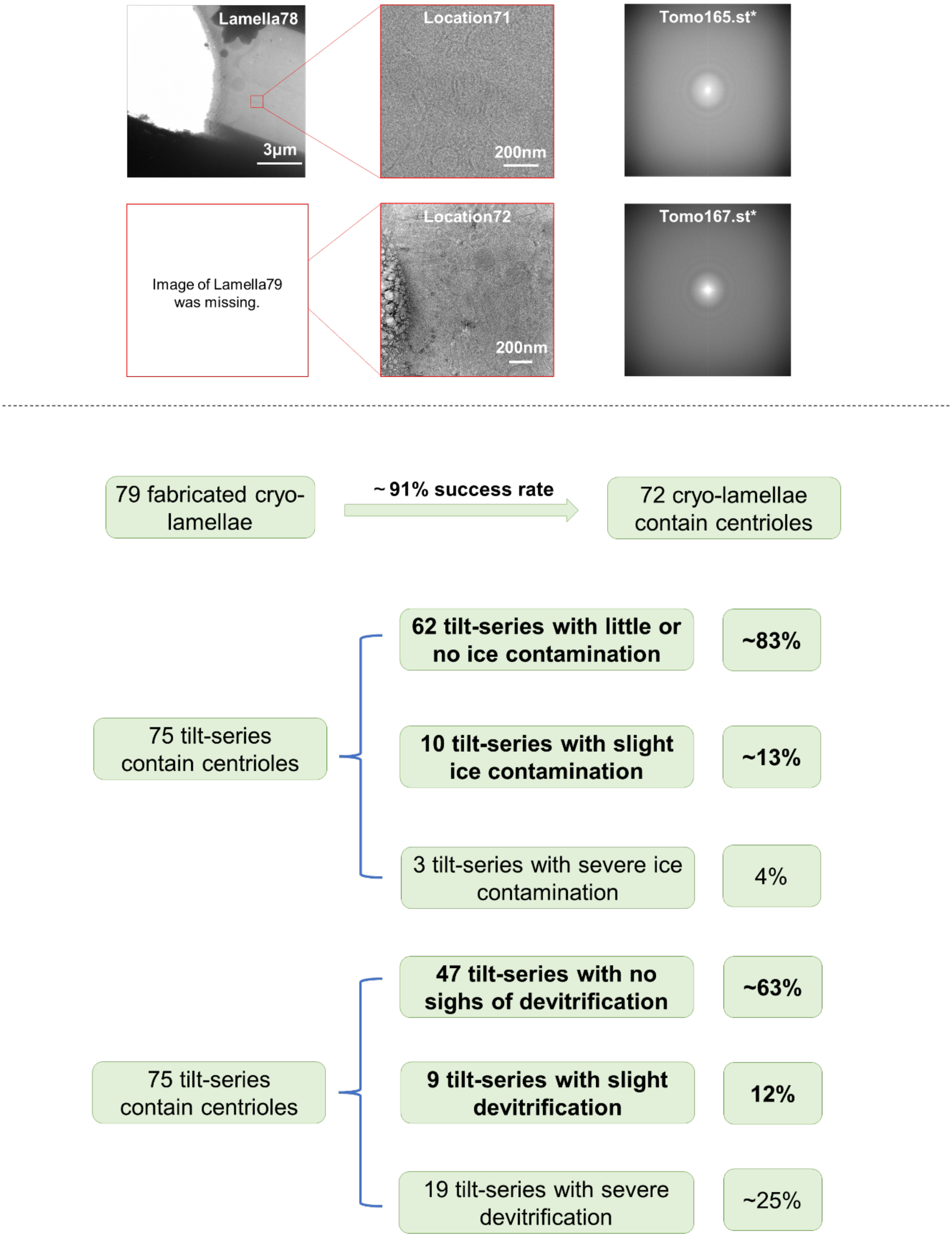


**Supplementary Figure 1. The list of cryo-EM images of all the cryo-lamellae fabricated in this study.** The low magnification cryo-EM image of each cryo-lamella is shown at the left (see Supplementary Data 1 for the

raw and uncompressed image) and the regions containing target centrioles are zoomed in and shown at the middle. The averaged power spectrum of the corresponding tilt series is shown at the right. The statistics of ice contamination and devitrification for all 75 tilt series are shown at the bottom. The tilt series with good quality used in the subsequent image processing are marked with asterisk (*) near their names, which have been deposited in EMPIAR (the Electron Microscopy Public Image Archive) China (http://www.emdb-china.org.cn) under accession code EMPIARC-200003.
