# Supplementary material for: ELI trifocal microscope: a precise system to prepare target cryo-lamellae for in situ cryo-ET study
Source: Nat Methods. 2023 Jan 16;20(2):276–83. doi: 10.1038/s41592-022-01748-0 (PMC9911351; doi:10.1038/s41592-022-01748-0)
Supplement: Supplementary file 2 — Reporting Summary [file 41592_2022_1748_MOESM2_ESM.pdf]

nature research

Corresponding author(s): Fei Sun, Gang Ji, Yun Zhu.

Last updated by author(s): Nov 6, 2022

Reporting Summary

Nature Research wishes to improve the reproducibility of the work that we publish. This form provides structure for consistency and transparency in reporting. For further information on Nature Research policies, see our Editorial Policies and the Editorial Policy Checklist.

Statistics

For all statistical analyses, confirm that the following items are present in the figure legend, table legend, main text, or Methods section.

n/a	Confirmed	
☐	☒	The exact sample size (*n*) for each experimental group/condition, given as a discrete number and unit of measurement
☐	☒	A statement on whether measurements were taken from distinct samples or whether the same sample was measured repeatedly
☒	☐	The statistical test(s) used AND whether they are one- or two-sided<br>*Only common tests should be described solely by name; describe more complex techniques in the Methods section.*
☒	☐	A description of all covariates tested
☒	☐	A description of any assumptions or corrections, such as tests of normality and adjustment for multiple comparisons
☐	☒	A full description of the statistical parameters including central tendency (e.g. means) or other basic estimates (e.g. regression coefficient) AND variation (e.g. standard deviation) or associated estimates of uncertainty (e.g. confidence intervals)
☒	☐	For null hypothesis testing, the test statistic (e.g. *F*, *t*, *r*) with confidence intervals, effect sizes, degrees of freedom and *P* value noted<br>*Give P values as exact values whenever suitable.*
☒	☐	For Bayesian analysis, information on the choice of priors and Markov chain Monte Carlo settings
☒	☐	For hierarchical and complex designs, identification of the appropriate level for tests and full reporting of outcomes
☒	☐	Estimates of effect sizes (e.g. Cohen's *d*, Pearson's *r*), indicating how they were calculated

Our web collection on statistics for biologists contains articles on many of the points above.

Software and code

Policy information about availability of computer code

Data collection	Cryo-ET data were automatically collected using SerialEM3.8 at Center for Biological Imaging (CBI, http://cbi.ibp.ac.cn), Institute of Biophysics, Chinese Academy of Sciences. Micro-Manager (ver 2.0) was used to control the camera and record fluorescence images. The movement and tilt of the stage can be controlled via customized software written in LabVIEW 2011 (National Instruments, USA). The LabVIEW program for device controlling is hardware-dependent and the code to control the stage of our ELI-TriScope is available at the GitHub: https://github.com/hilbertsun/ELI-TriScope.
Data analysis	Cryo-EM data were analyzed using the RELION3.1, RELION3.0, UCSF-ChimeraX 1.3, IMOD 4.12.16, Imaris 9.8.0, Warp1.0.9.

For manuscripts utilizing custom algorithms or software that are central to the research but not yet described in published literature, software must be made available to editors and reviewers. We strongly encourage code deposition in a community repository (e.g. GitHub). See the Nature Research guidelines for submitting code & software for further information.

Data

Policy information about availability of data

All manuscripts must include a data availability statement. This statement should provide the following information, where applicable:

- Accession codes, unique identifiers, or web links for publicly available datasets
- A list of figures that have associated raw data
- A description of any restrictions on data availability

The raw tilt series used in this study has been deposited in EMPIAR (the Electron Microscopy Public Image Archive) China (http://www.emdb-china.org.cn) under accession code EMPIARC-200003. The sub-tomogram averaged cryo-EM maps of the MTTs with complete C tubule, MTTs with incomplete C tubule, A tubule, A-C linker and pinhead have been deposited in the Electron Microscopy Database (EMDB) with the accession codes EMD-33417, EMD-33418, EMD-33419, EMD-33420 and EMD-33421, respectively. All other data that support the conclusion of this study are provided in the supplementary data and source data.

Field-specific reporting

Please select the one below that is the best fit for your research. If you are not sure, read the appropriate sections before making your selection.

☒ Life sciences ☐ Behavioural & social sciences ☐ Ecological, evolutionary & environmental sciences

For a reference copy of the document with all sections, see nature.com/documents/nr-reporting-summary-flat.pdf

Life sciences study design

All studies must disclose on these points even when the disclosure is negative.

Sample size	We prepared 40 vitrified cryo-grids, and 21 of them showed good quality (proper ice thickness and well distributed fluorescent signals) in the cryo-FM screening and were used for ELI-TriScope milling. We fabricated a total of 79 cryo-lamellae, and found 72 of them containing centrioles, which were used for the subsequent cryo-ET data collection. During sub-tomogram averaging process, the number of final particles that went into each refined map were determined through 3D classification as described in Extended Data Fig. 4. The sample size is limited by the resources of microscope time and the current size has been enough to determine the successful rate of our ELI-TriScope workflow.
Data exclusions	Tomograms with poor quality or unable to be aligned were excluded.
Replication	To measure the successful rate of our ELI-TriScope workflow, we repeated performing ELI-TriScope milling of 21 vitrified grids and the number of repeating ELI-TriScope workflow is 79 and we found 72 of them were successful to target the regions containing centrioles.
Randomization	Randomization was not applicable because there was nothing related to comparison between control and experimental groups in this study.
Blinding	Blinding was not applicable because there was nothing related to comparison among different groups in this study.

Reporting for specific materials, systems and methods

We require information from authors about some types of materials, experimental systems and methods used in many studies. Here, indicate whether each material, system or method listed is relevant to your study. If you are not sure if a list item applies to your research, read the appropriate section before selecting a response.

Materials & experimental systems

n/a	Involved in the study	
☒	☐	Antibodies
☐	☒	Eukaryotic cell lines
☒	☐	Palaeontology and archaeology
☒	☐	Animals and other organisms
☒	☐	Human research participants
☒	☐	Clinical data
☒	☐	Dual use research of concern

Methods

n/a	Involved in the study	
☒	☐	ChIP-seq
☒	☐	Flow cytometry
☒	☐	MRI-based neuroimaging

Eukaryotic cell lines

Policy information about cell lines

Cell line source(s)	HeLa (ATCC: CCL-2) cells
Authentication	HeLa (ATCC: CCL-2) cells were obtained from ATCC and provided from Prof. Jianguo Chen's lab in Peking University and the cell lines were made according to the literature (EMBO Reports 1, 524-529)
Mycoplasma contamination	Not detected
Commonly misidentified lines (See ICLAC register)	No commonly misidentified cell lines were used.
