# Supplementary figures and images for: ELI trifocal microscope: a precise system to prepare target cryo-lamellae for in situ cryo-ET study
Source: Nat Methods. 2023 Jan 16;20(2):276–83. doi: 10.1038/s41592-022-01748-0 (PMC9911351; doi:10.1038/s41592-022-01748-0)

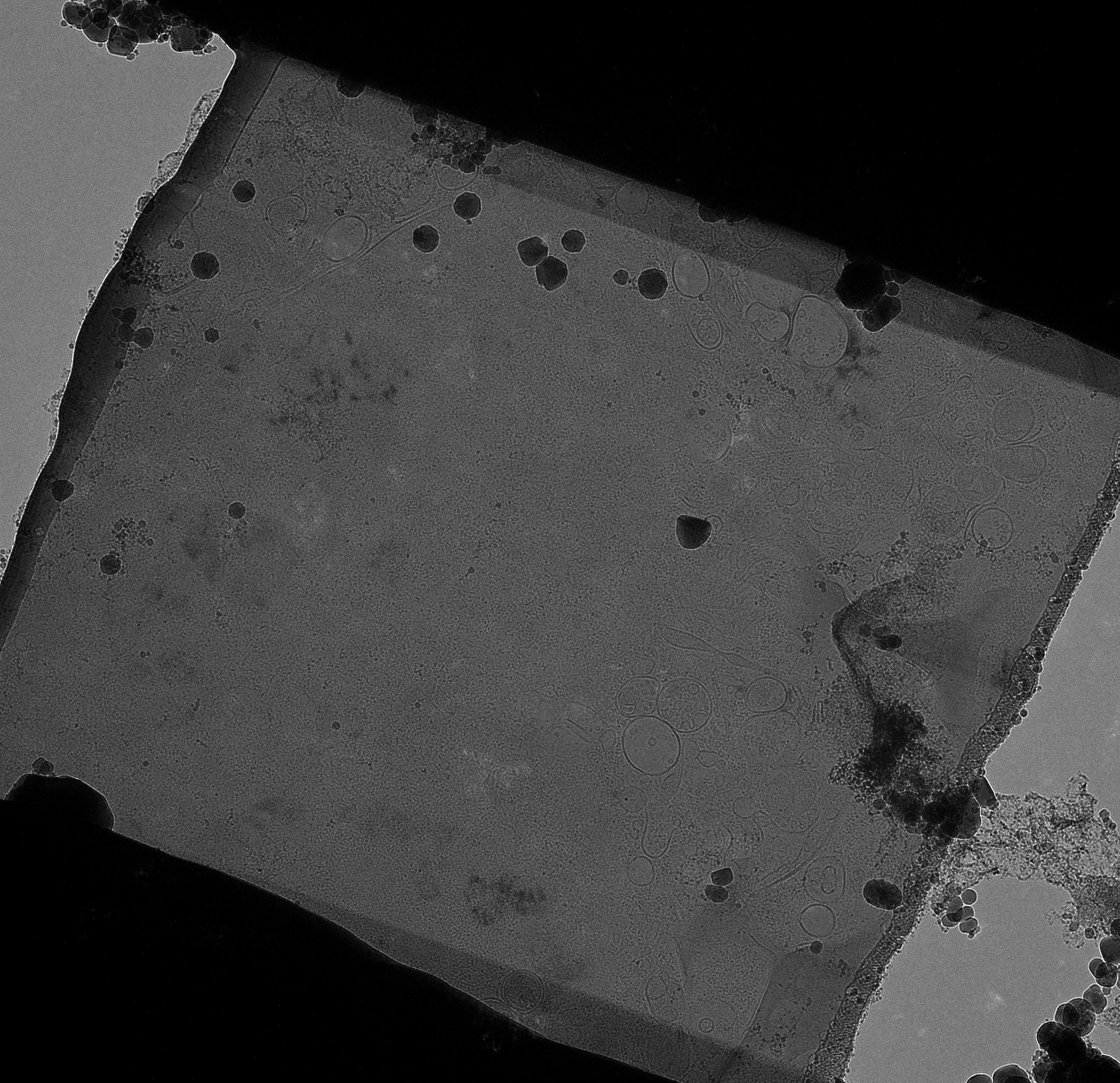

Supplement: Supplementary file 8 — Raw cryo-EM images of all the cryo-lamellae shown in Supplementary Fig. 1. The locations of centrioles are marked by dashed squares. [file 41592_2022_1748_MOESM8_ESM.zip › Supplementary_Data1/Lamella42_NoLocation.jpg]

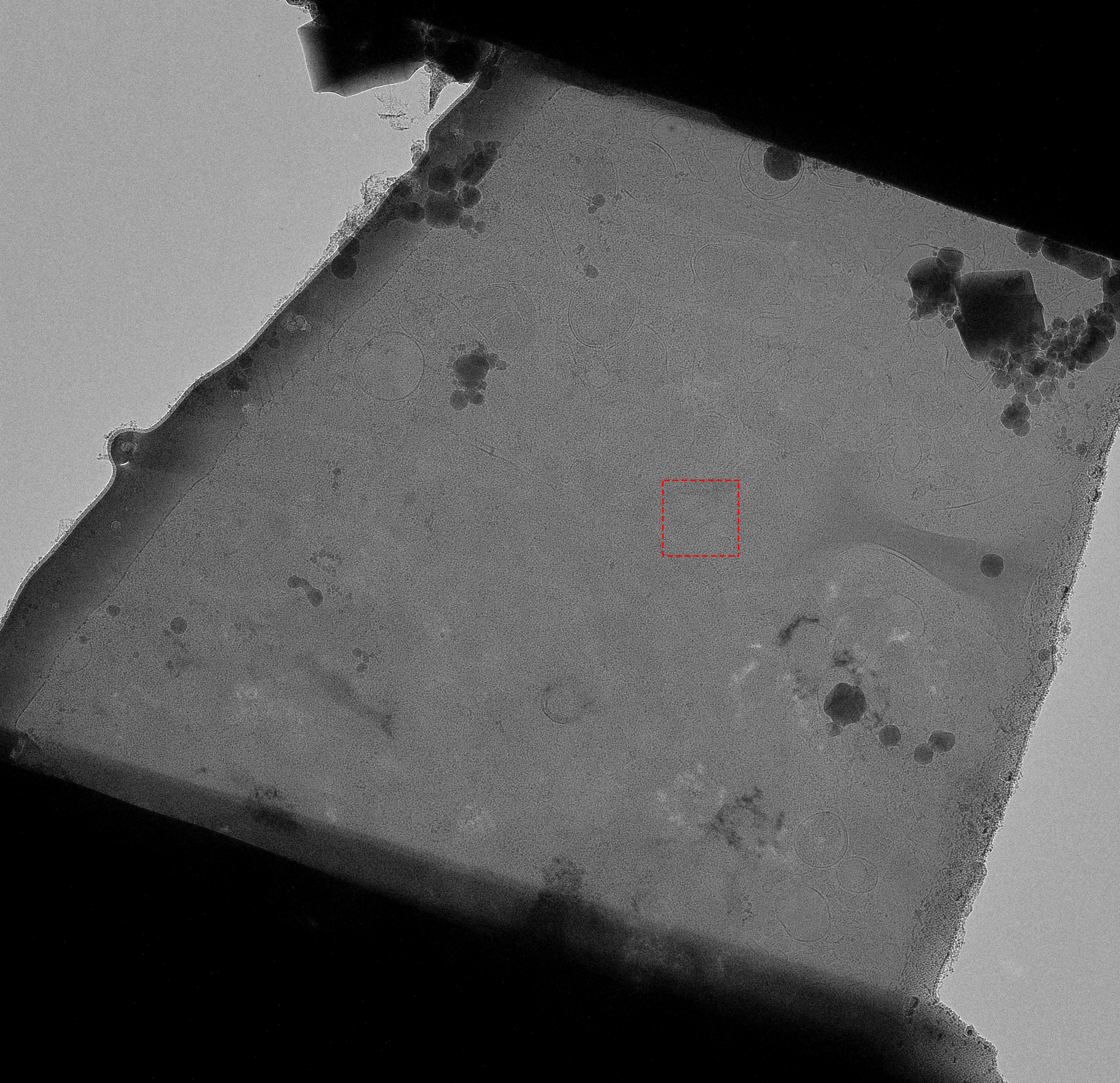

Supplement: Supplementary file 8 — Raw cryo-EM images of all the cryo-lamellae shown in Supplementary Fig. 1. The locations of centrioles are marked by dashed squares. [file 41592_2022_1748_MOESM8_ESM.zip › Supplementary_Data1/Lamella40_Location38.jpg]

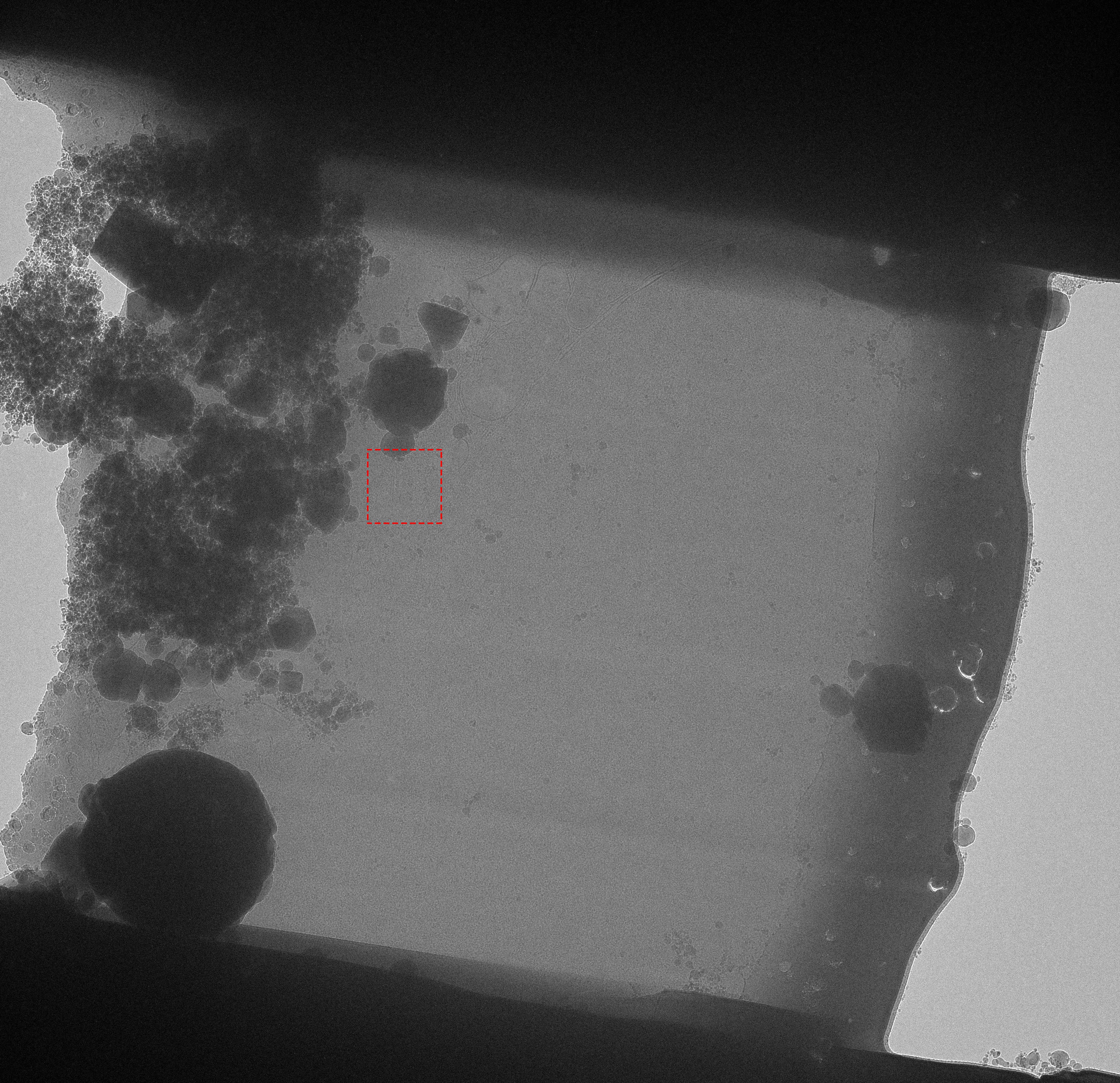

Supplement: Supplementary file 8 — Raw cryo-EM images of all the cryo-lamellae shown in Supplementary Fig. 1. The locations of centrioles are marked by dashed squares. [file 41592_2022_1748_MOESM8_ESM.zip › Supplementary_Data1/Lamella30_Location29.jpg]

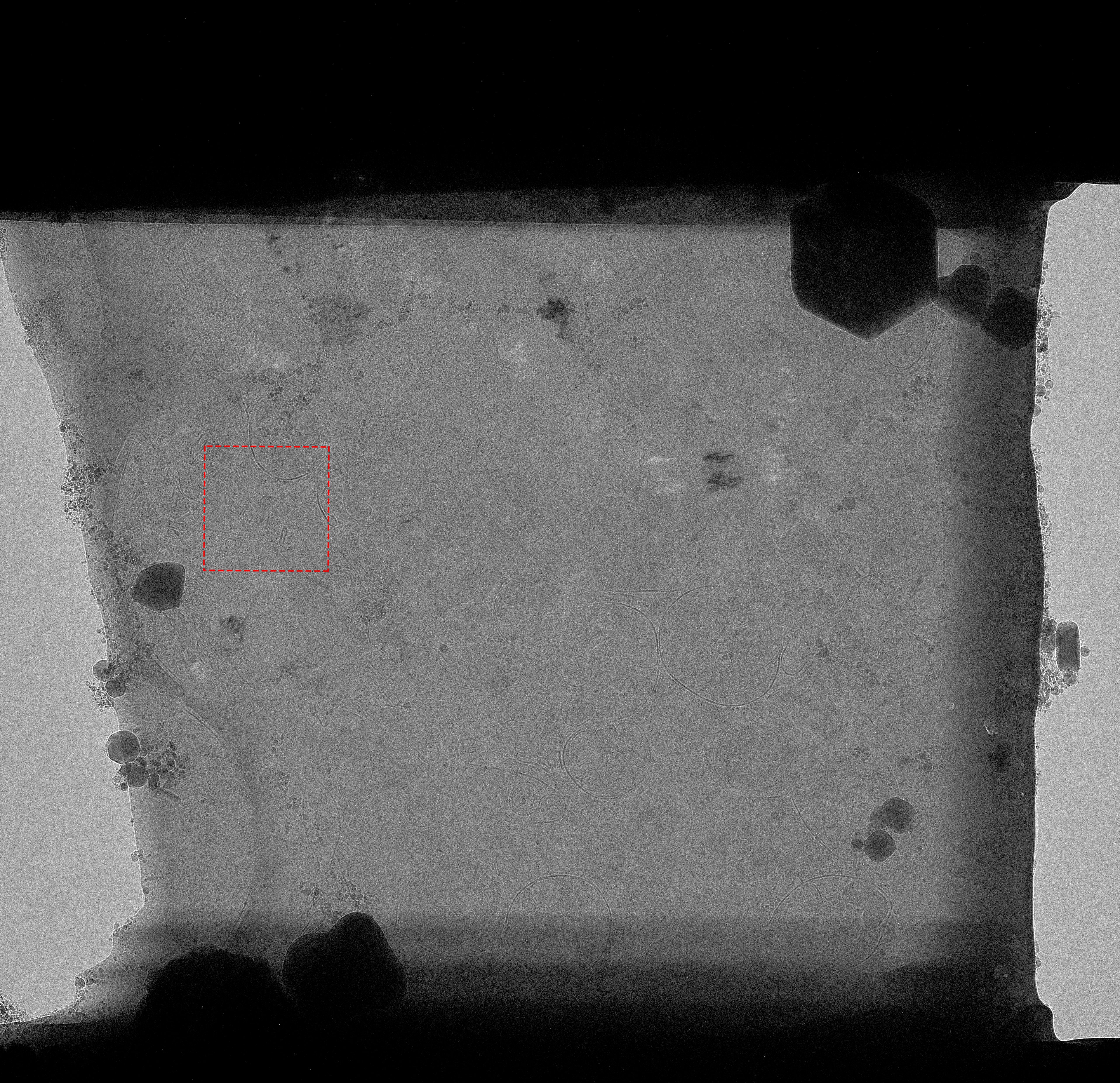

Supplement: Supplementary file 8 — Raw cryo-EM images of all the cryo-lamellae shown in Supplementary Fig. 1. The locations of centrioles are marked by dashed squares. [file 41592_2022_1748_MOESM8_ESM.zip › Supplementary_Data1/Lamella19_Location18.jpg]

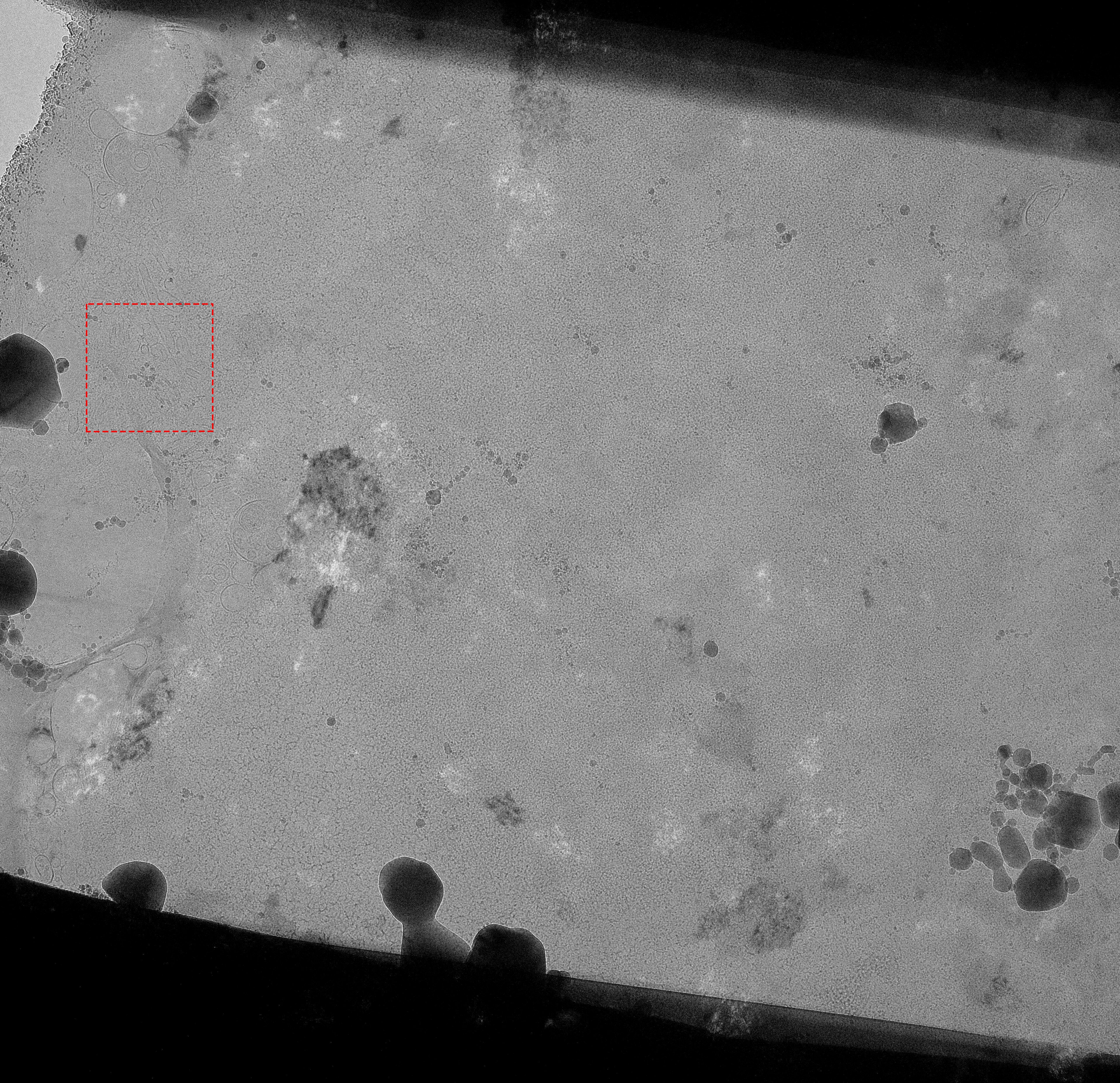

Supplement: Supplementary file 8 — Raw cryo-EM images of all the cryo-lamellae shown in Supplementary Fig. 1. The locations of centrioles are marked by dashed squares. [file 41592_2022_1748_MOESM8_ESM.zip › Supplementary_Data1/Lamella51_Location48.jpg]

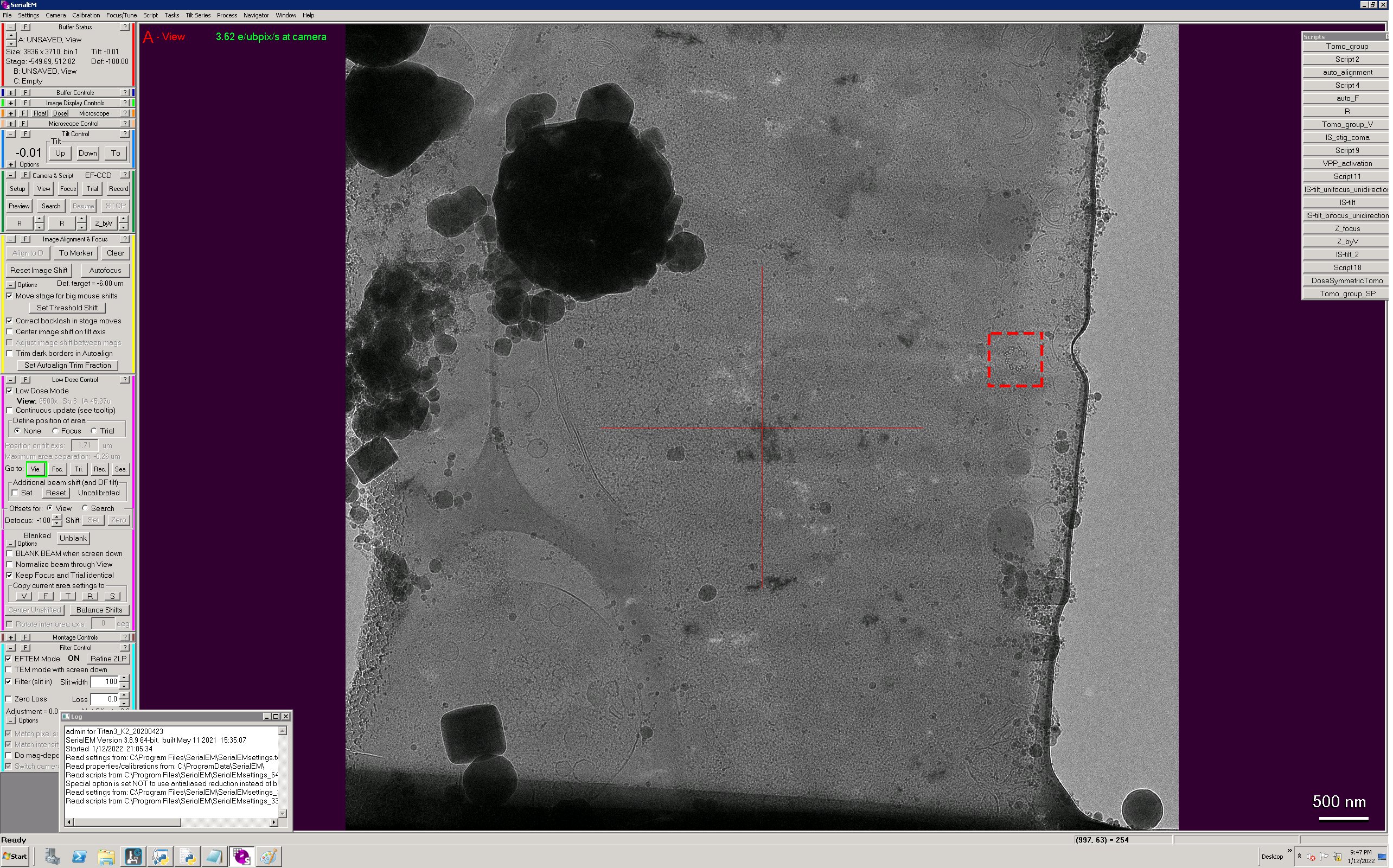

Supplement: Supplementary file 8 — Raw cryo-EM images of all the cryo-lamellae shown in Supplementary Fig. 1. The locations of centrioles are marked by dashed squares. [file 41592_2022_1748_MOESM8_ESM.zip › Supplementary_Data1/Lamella63_Location57.jpg]

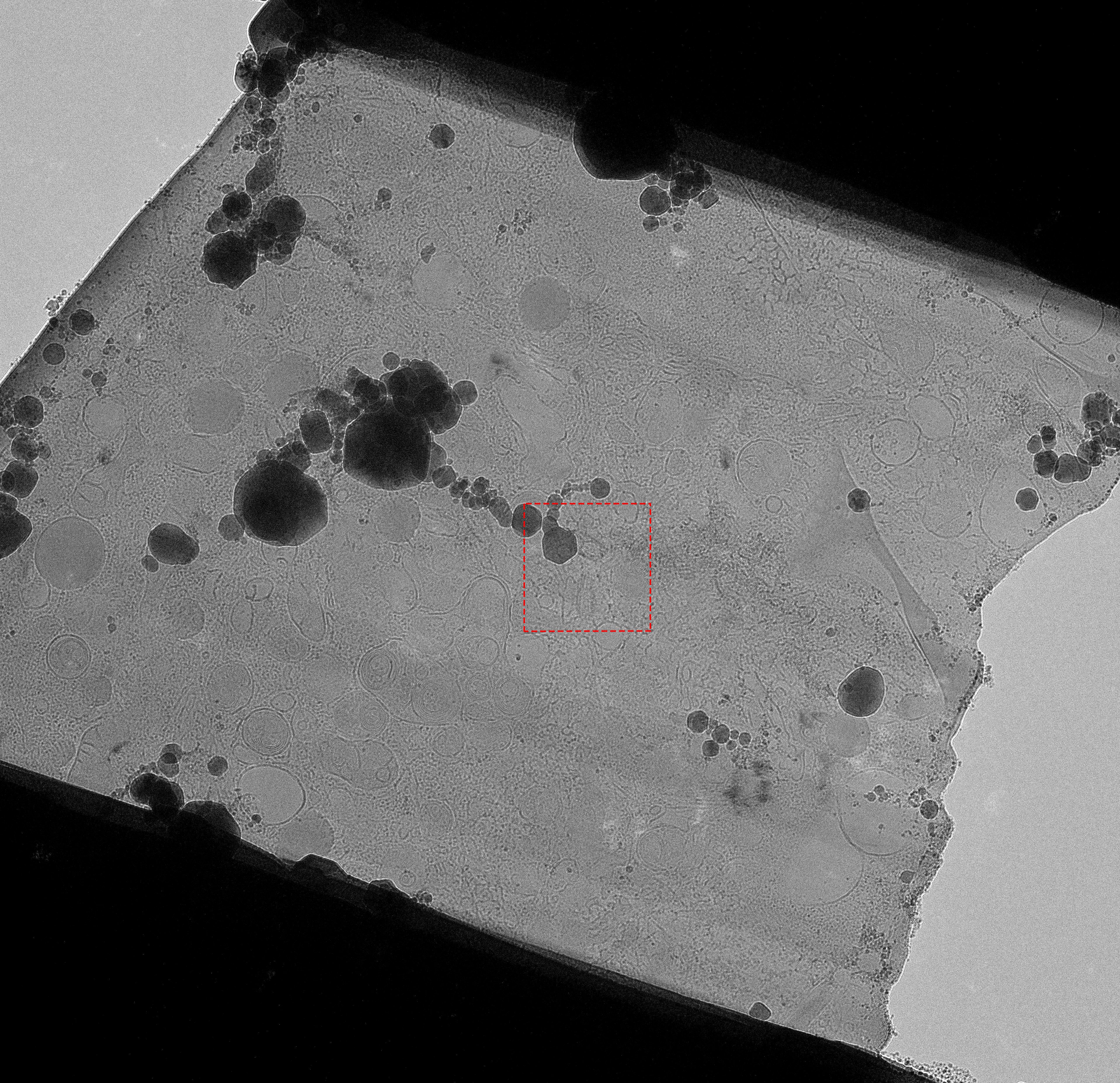

Supplement: Supplementary file 8 — Raw cryo-EM images of all the cryo-lamellae shown in Supplementary Fig. 1. The locations of centrioles are marked by dashed squares. [file 41592_2022_1748_MOESM8_ESM.zip › Supplementary_Data1/Lamella41_Location39.jpg]

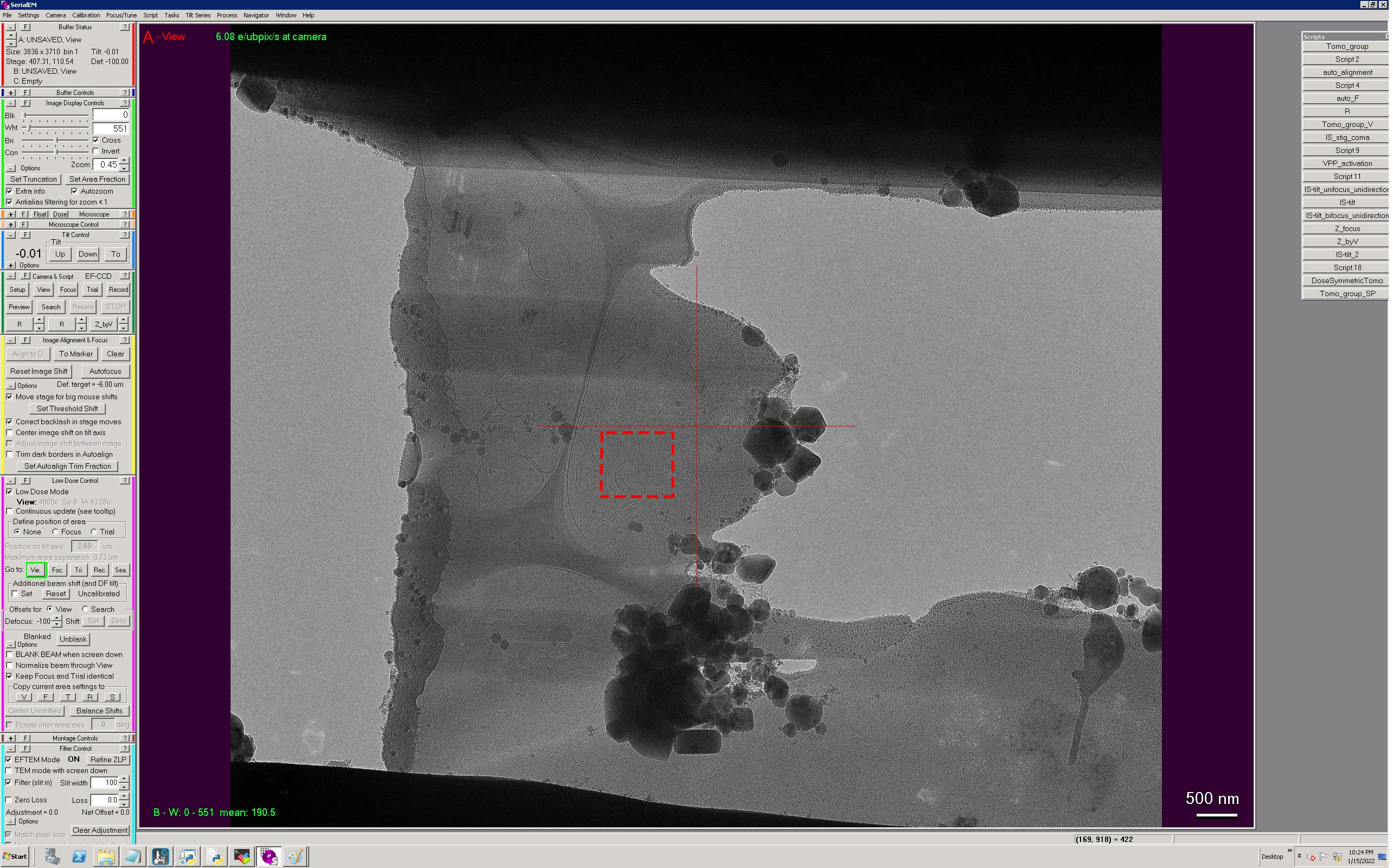

Supplement: Supplementary file 8 — Raw cryo-EM images of all the cryo-lamellae shown in Supplementary Fig. 1. The locations of centrioles are marked by dashed squares. [file 41592_2022_1748_MOESM8_ESM.zip › Supplementary_Data1/Lamella76_Location69.jpg]

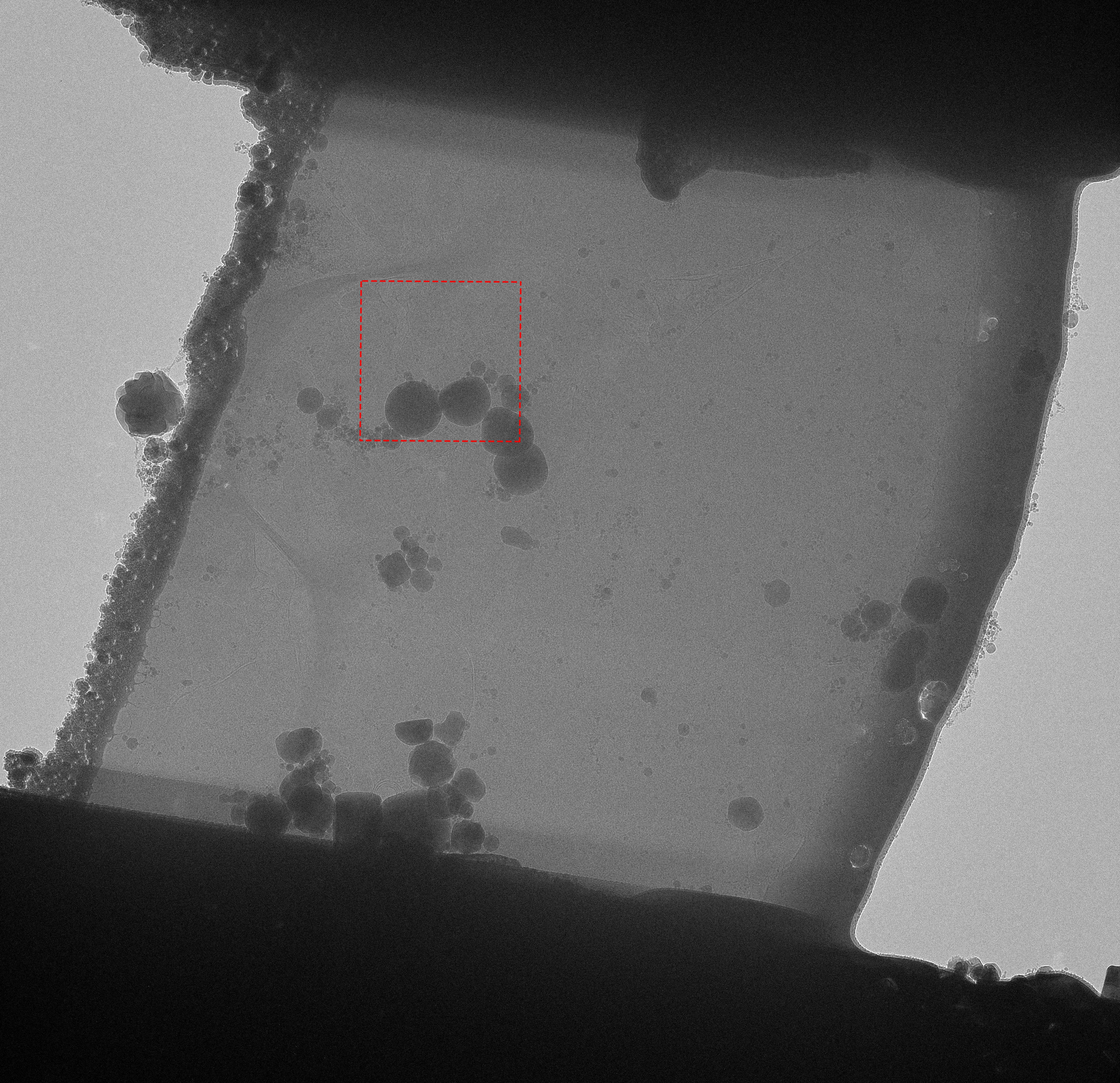

Supplement: Supplementary file 8 — Raw cryo-EM images of all the cryo-lamellae shown in Supplementary Fig. 1. The locations of centrioles are marked by dashed squares. [file 41592_2022_1748_MOESM8_ESM.zip › Supplementary_Data1/Lamella29_Location28.jpg]

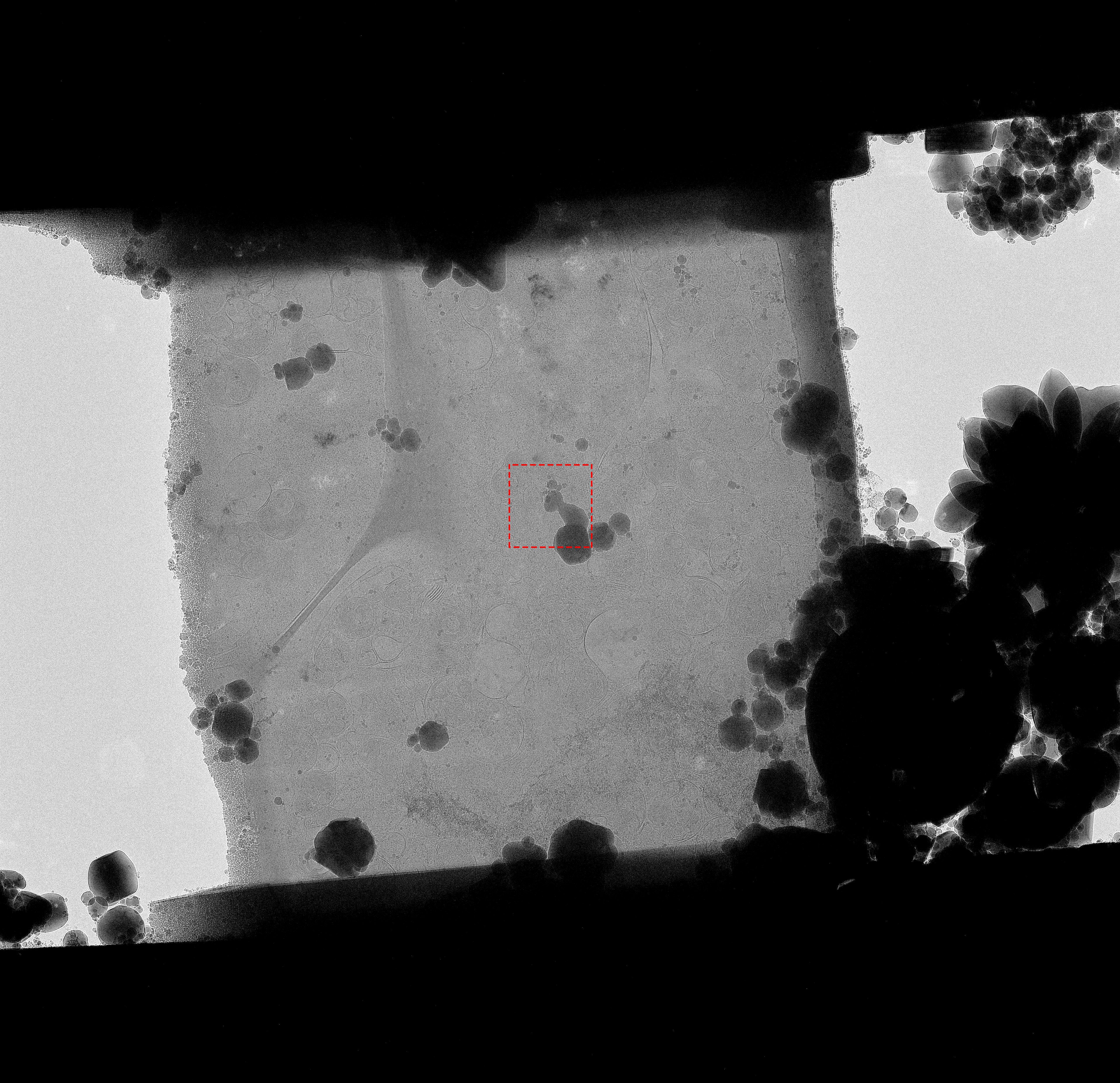

Supplement: Supplementary file 8 — Raw cryo-EM images of all the cryo-lamellae shown in Supplementary Fig. 1. The locations of centrioles are marked by dashed squares. [file 41592_2022_1748_MOESM8_ESM.zip › Supplementary_Data1/Lamella09_Location09.jpg]

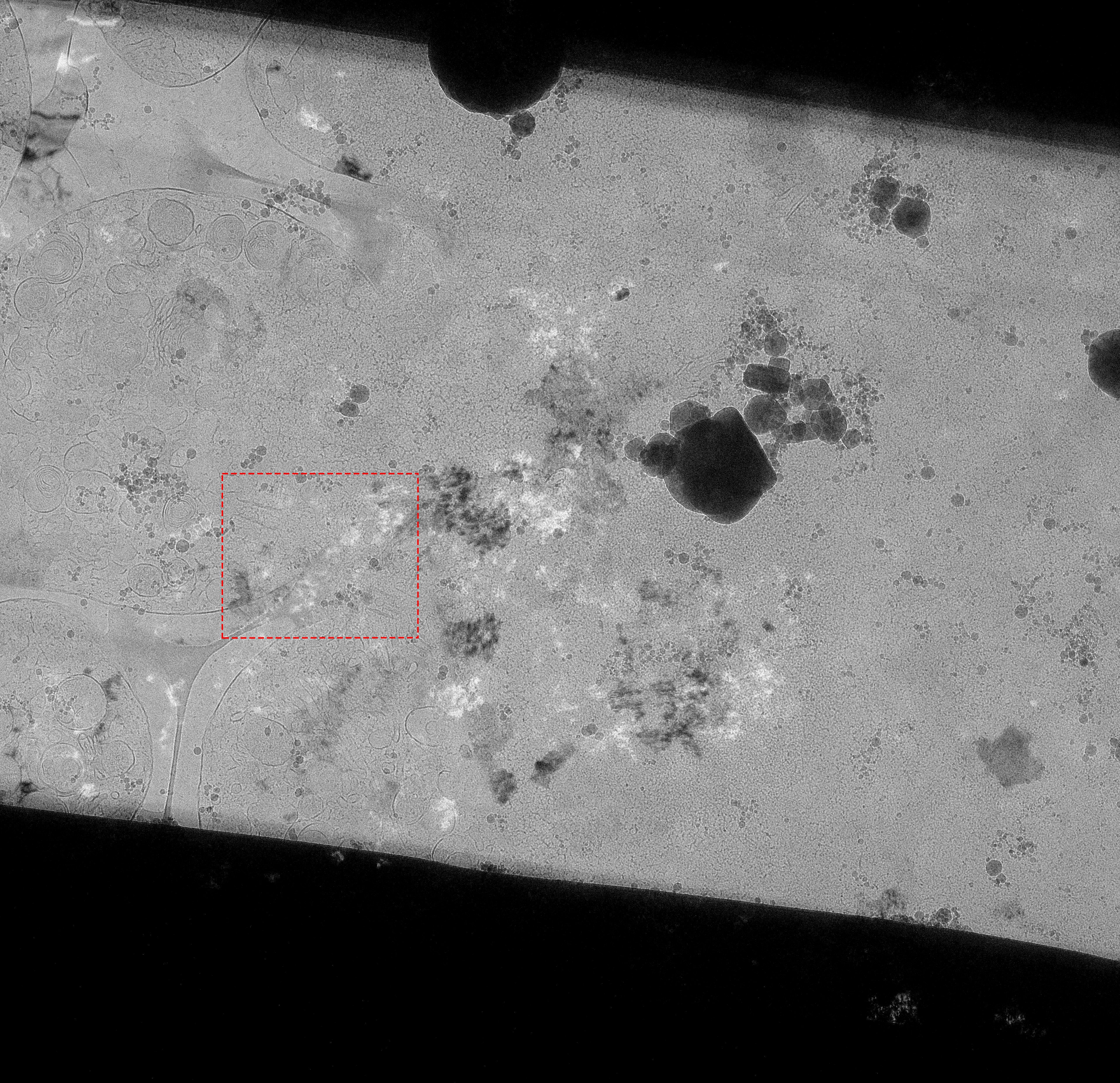

Supplement: Supplementary file 8 — Raw cryo-EM images of all the cryo-lamellae shown in Supplementary Fig. 1. The locations of centrioles are marked by dashed squares. [file 41592_2022_1748_MOESM8_ESM.zip › Supplementary_Data1/Lamella52_Location49.jpg]

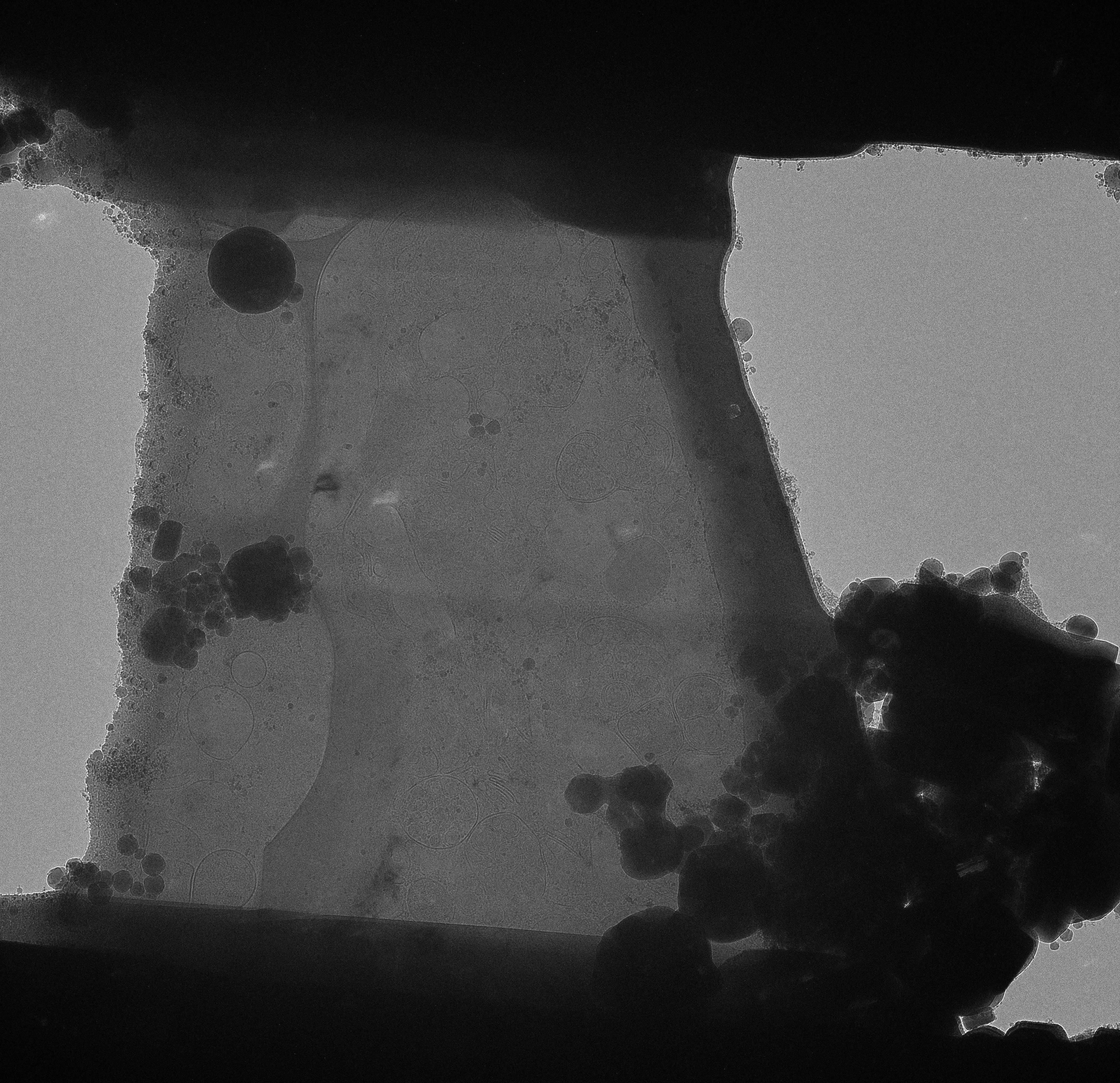

Supplement: Supplementary file 8 — Raw cryo-EM images of all the cryo-lamellae shown in Supplementary Fig. 1. The locations of centrioles are marked by dashed squares. [file 41592_2022_1748_MOESM8_ESM.zip › Supplementary_Data1/Lamella31_NoLocation.jpg]

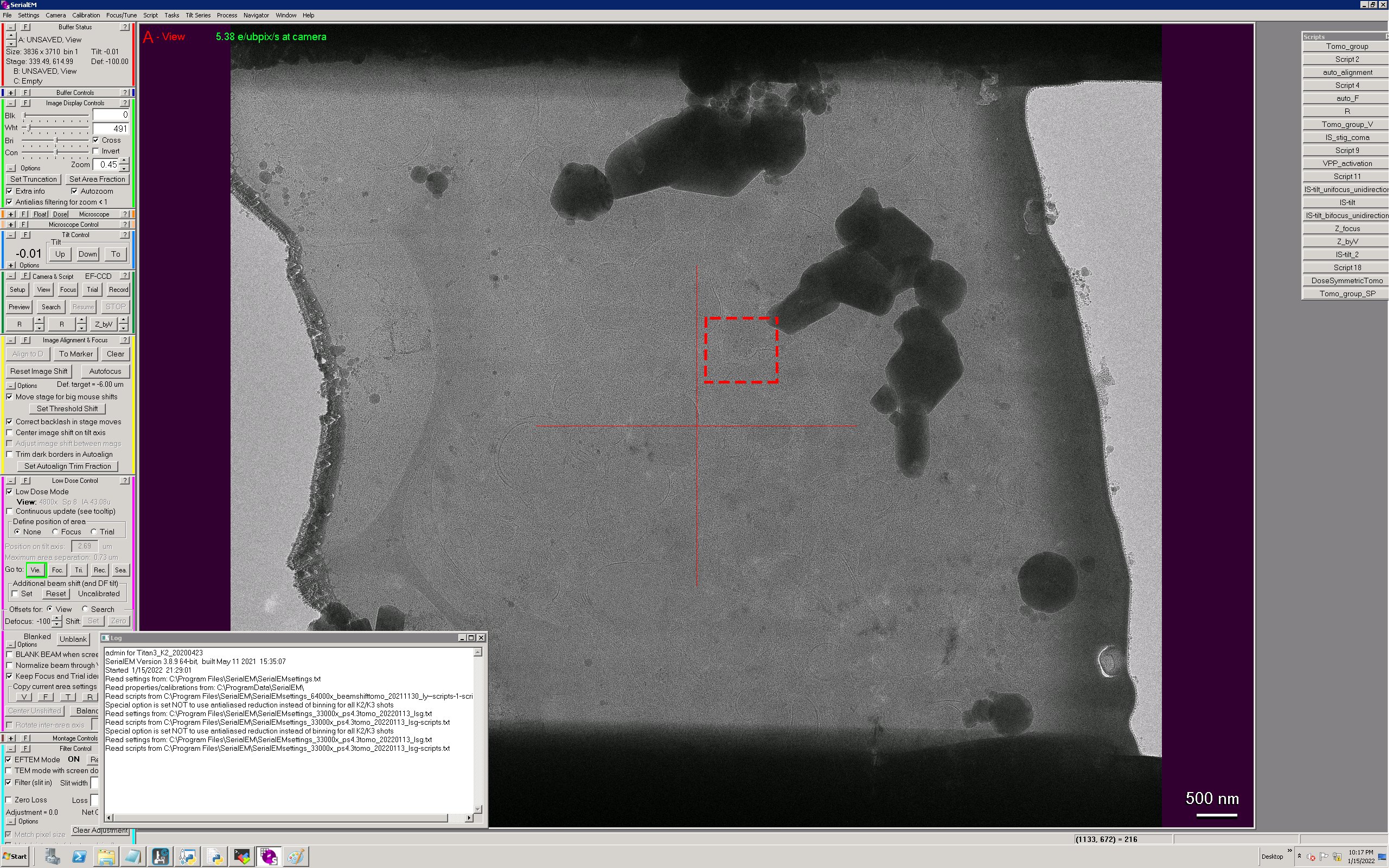

Supplement: Supplementary file 8 — Raw cryo-EM images of all the cryo-lamellae shown in Supplementary Fig. 1. The locations of centrioles are marked by dashed squares. [file 41592_2022_1748_MOESM8_ESM.zip › Supplementary_Data1/Lamella75_Location68.jpg]

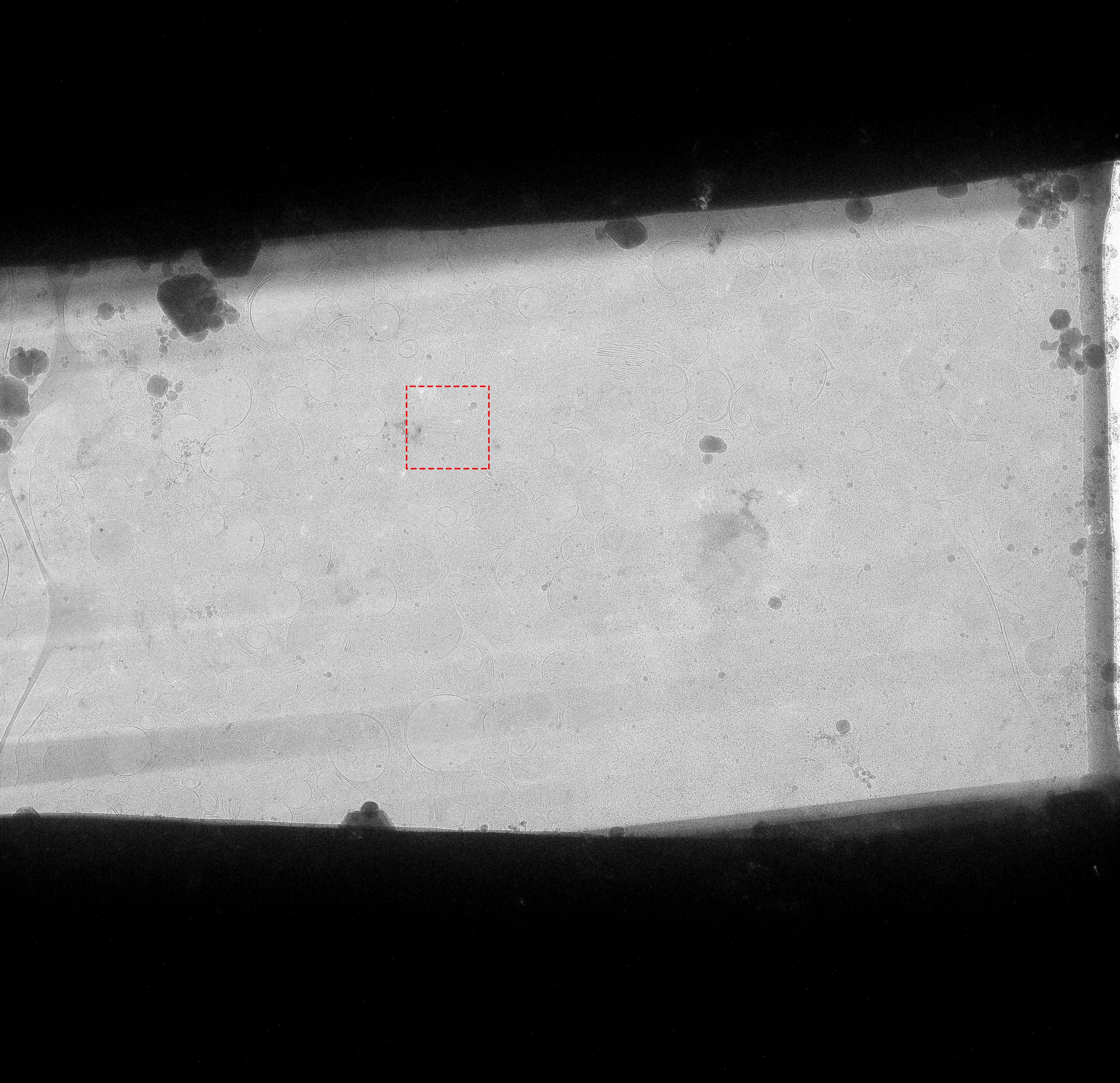

Supplement: Supplementary file 8 — Raw cryo-EM images of all the cryo-lamellae shown in Supplementary Fig. 1. The locations of centrioles are marked by dashed squares. [file 41592_2022_1748_MOESM8_ESM.zip › Supplementary_Data1/Lamella08_Location08.jpg]

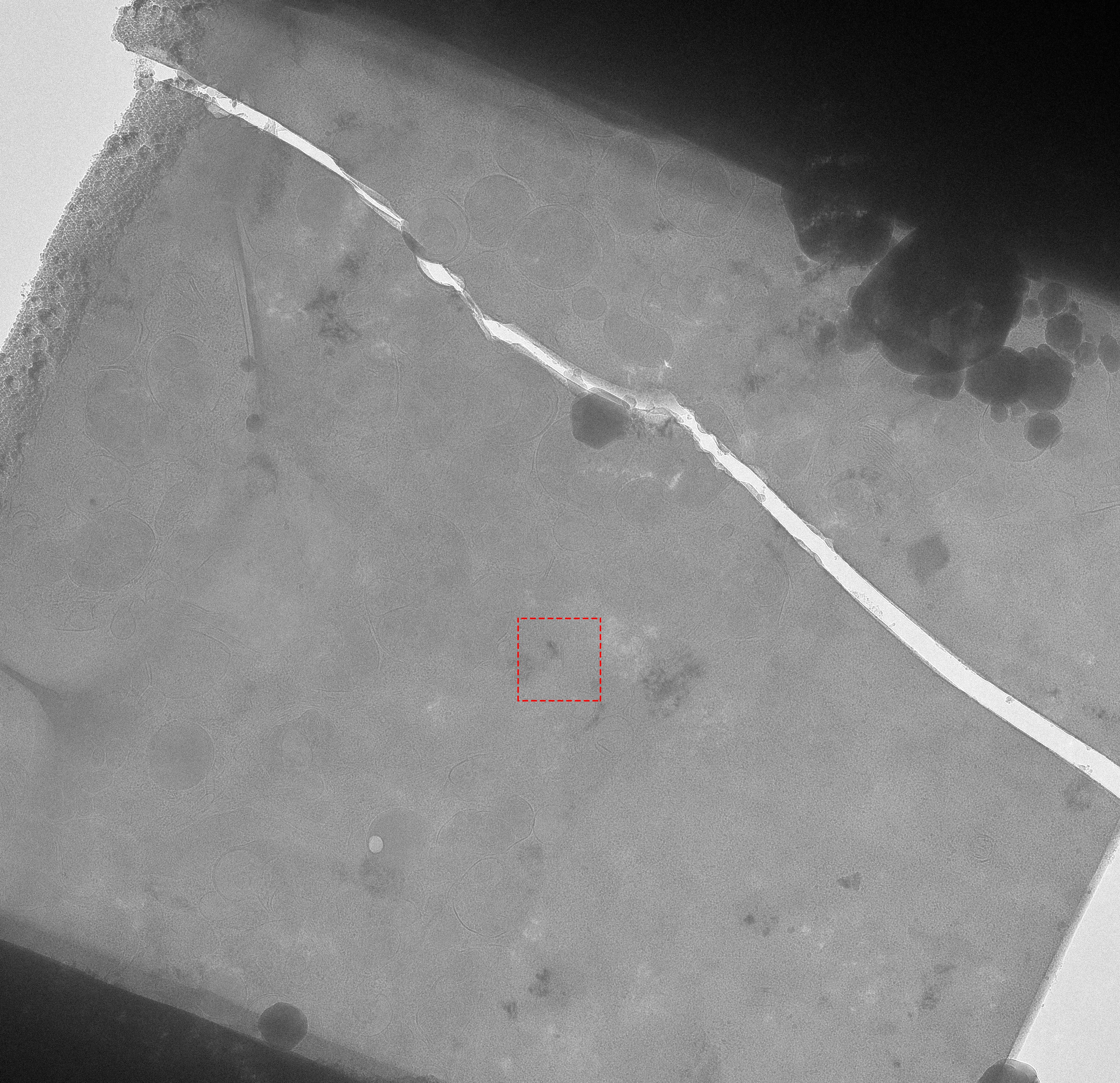

Supplement: Supplementary file 8 — Raw cryo-EM images of all the cryo-lamellae shown in Supplementary Fig. 1. The locations of centrioles are marked by dashed squares. [file 41592_2022_1748_MOESM8_ESM.zip › Supplementary_Data1/Lamella07_Location07.jpg]

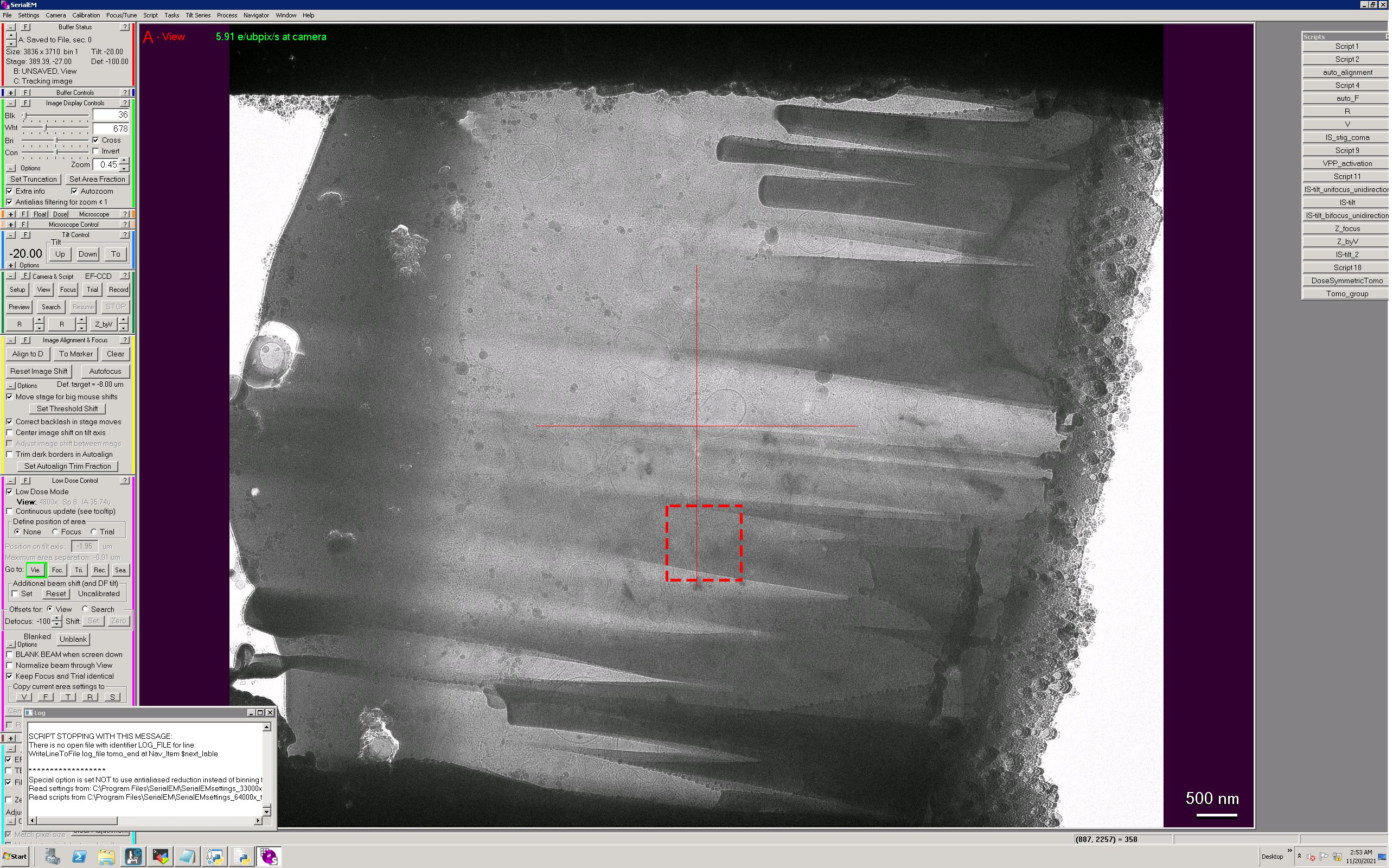

Supplement: Supplementary file 8 — Raw cryo-EM images of all the cryo-lamellae shown in Supplementary Fig. 1. The locations of centrioles are marked by dashed squares. [file 41592_2022_1748_MOESM8_ESM.zip › Supplementary_Data1/Lamella21_Location20.jpg]

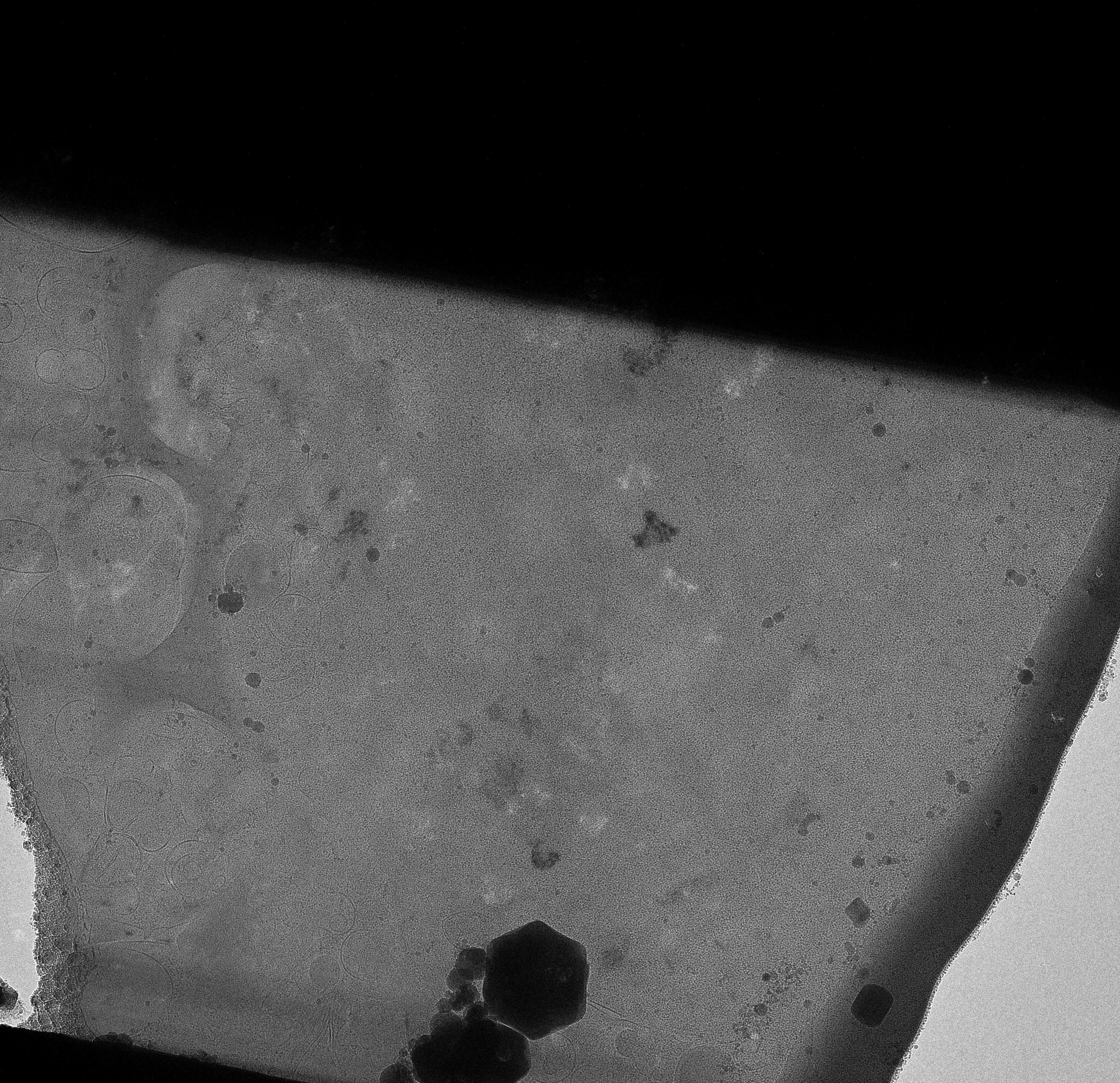

Supplement: Supplementary file 8 — Raw cryo-EM images of all the cryo-lamellae shown in Supplementary Fig. 1. The locations of centrioles are marked by dashed squares. [file 41592_2022_1748_MOESM8_ESM.zip › Supplementary_Data1/Lamella61_NoLocation.jpg]

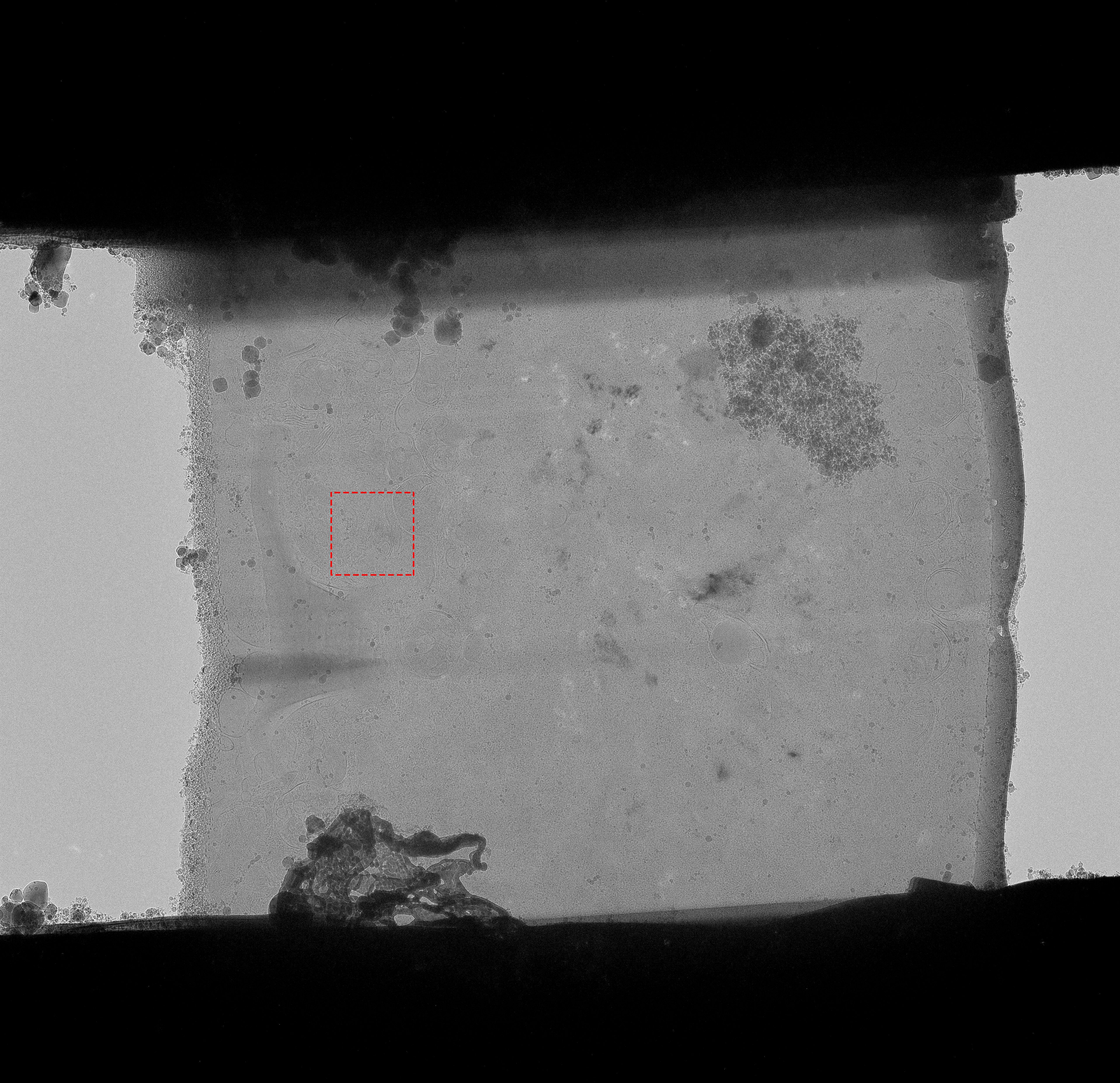

Supplement: Supplementary file 8 — Raw cryo-EM images of all the cryo-lamellae shown in Supplementary Fig. 1. The locations of centrioles are marked by dashed squares. [file 41592_2022_1748_MOESM8_ESM.zip › Supplementary_Data1/Lamella14_Location14.jpg]

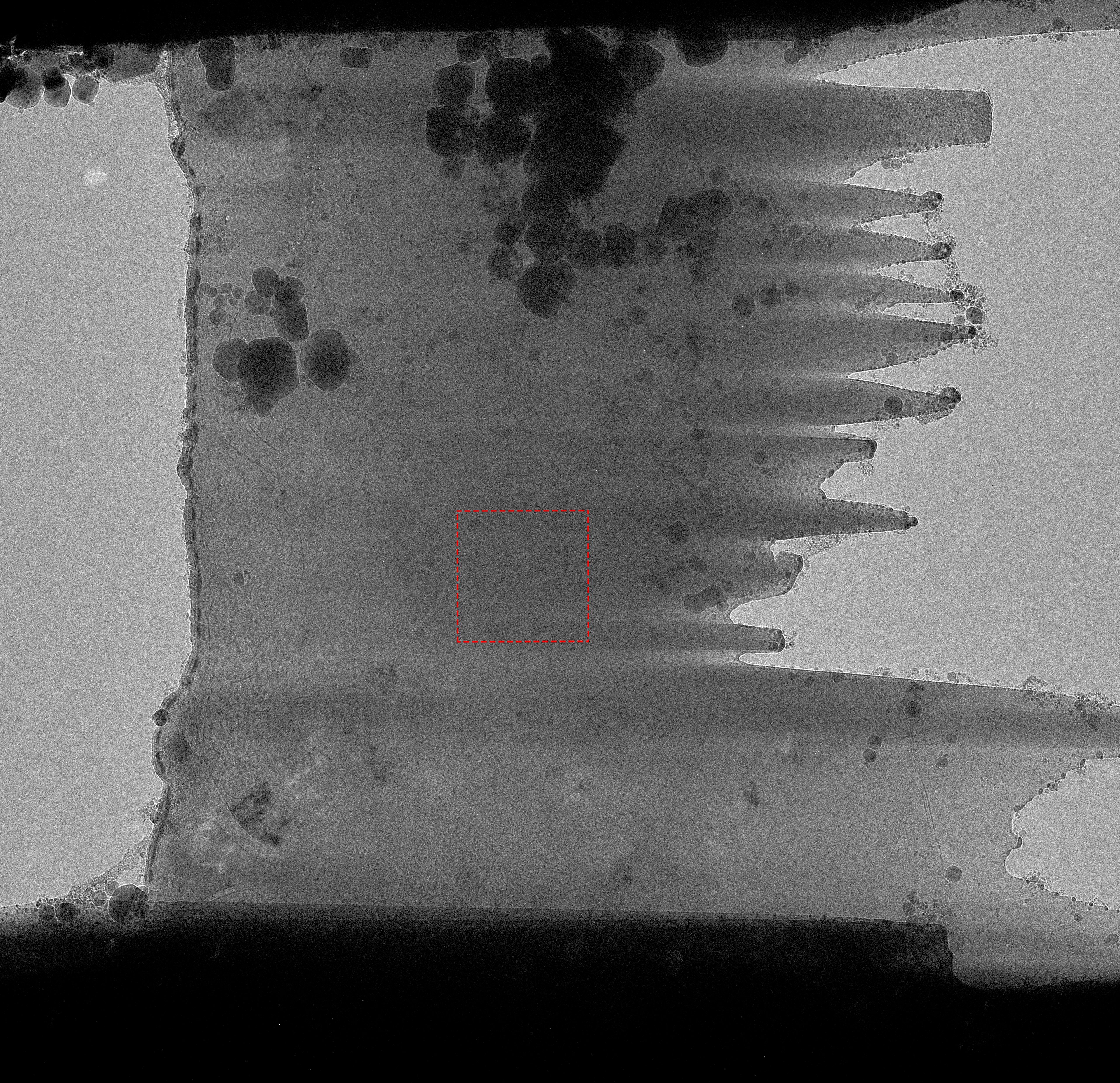

Supplement: Supplementary file 8 — Raw cryo-EM images of all the cryo-lamellae shown in Supplementary Fig. 1. The locations of centrioles are marked by dashed squares. [file 41592_2022_1748_MOESM8_ESM.zip › Supplementary_Data1/Lamella20_Location19.jpg]

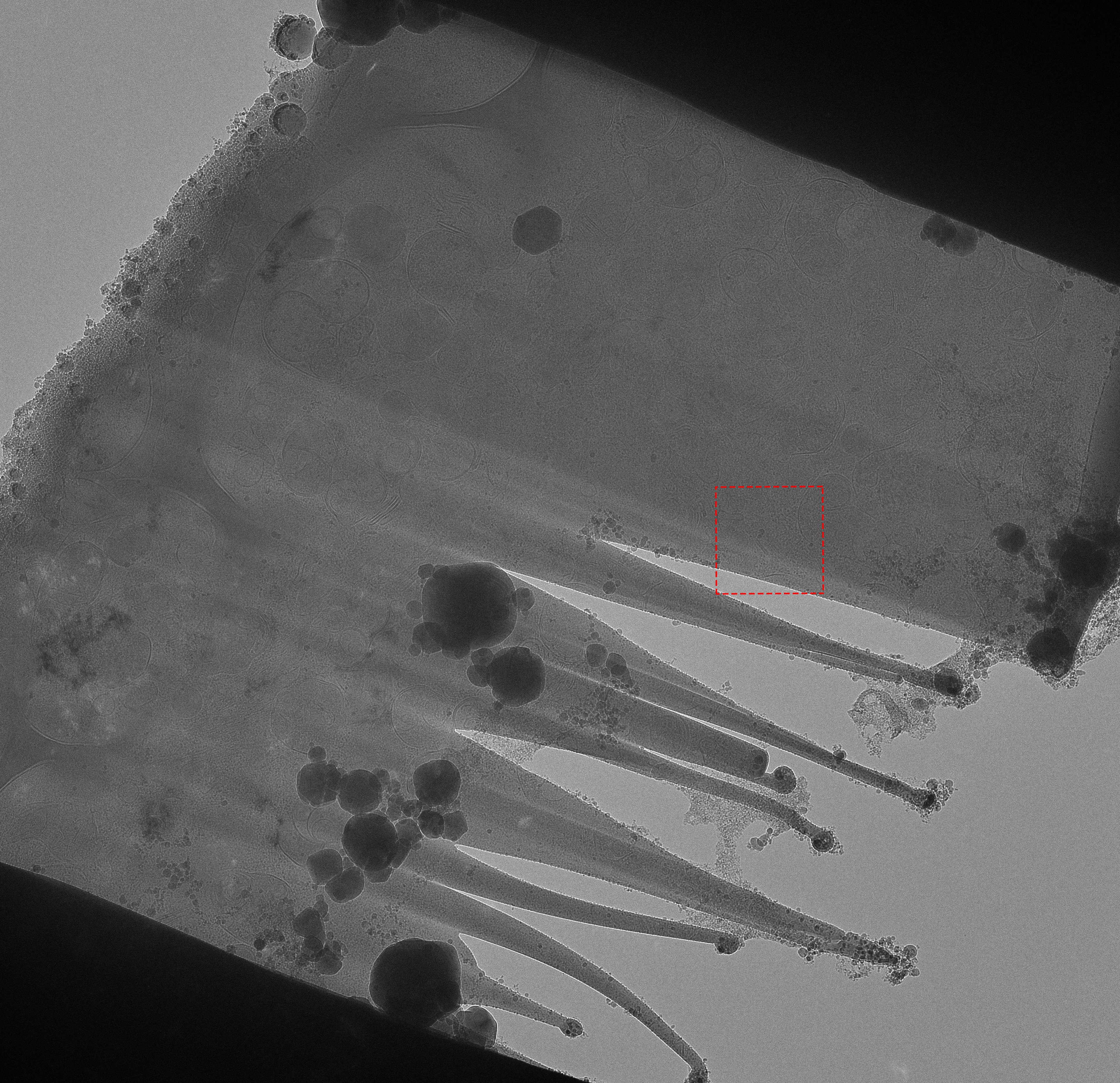

Supplement: Supplementary file 8 — Raw cryo-EM images of all the cryo-lamellae shown in Supplementary Fig. 1. The locations of centrioles are marked by dashed squares. [file 41592_2022_1748_MOESM8_ESM.zip › Supplementary_Data1/Lamella01_Location01.jpg]

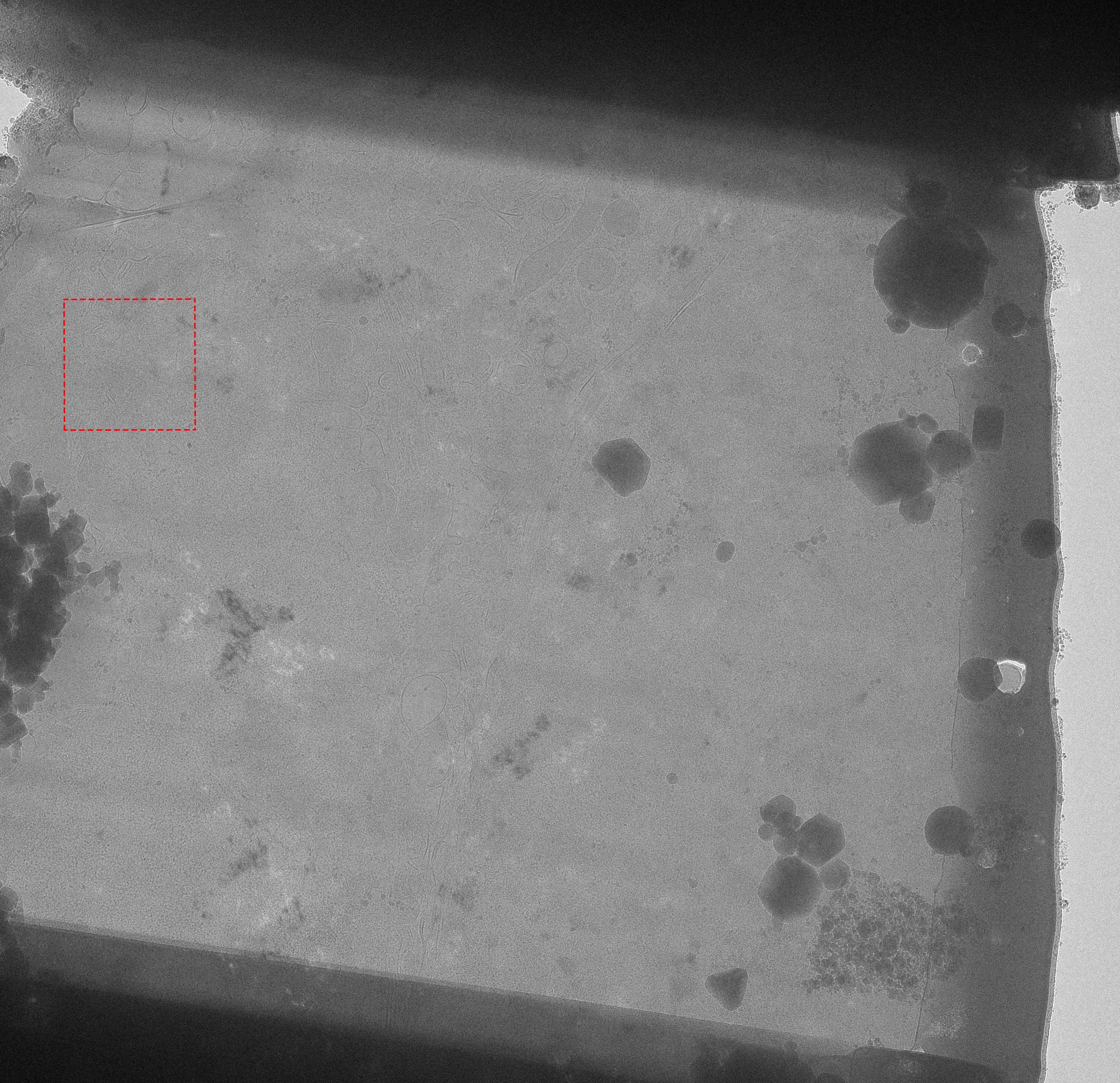

Supplement: Supplementary file 8 — Raw cryo-EM images of all the cryo-lamellae shown in Supplementary Fig. 1. The locations of centrioles are marked by dashed squares. [file 41592_2022_1748_MOESM8_ESM.zip › Supplementary_Data1/Lamella27_Location26.jpg]

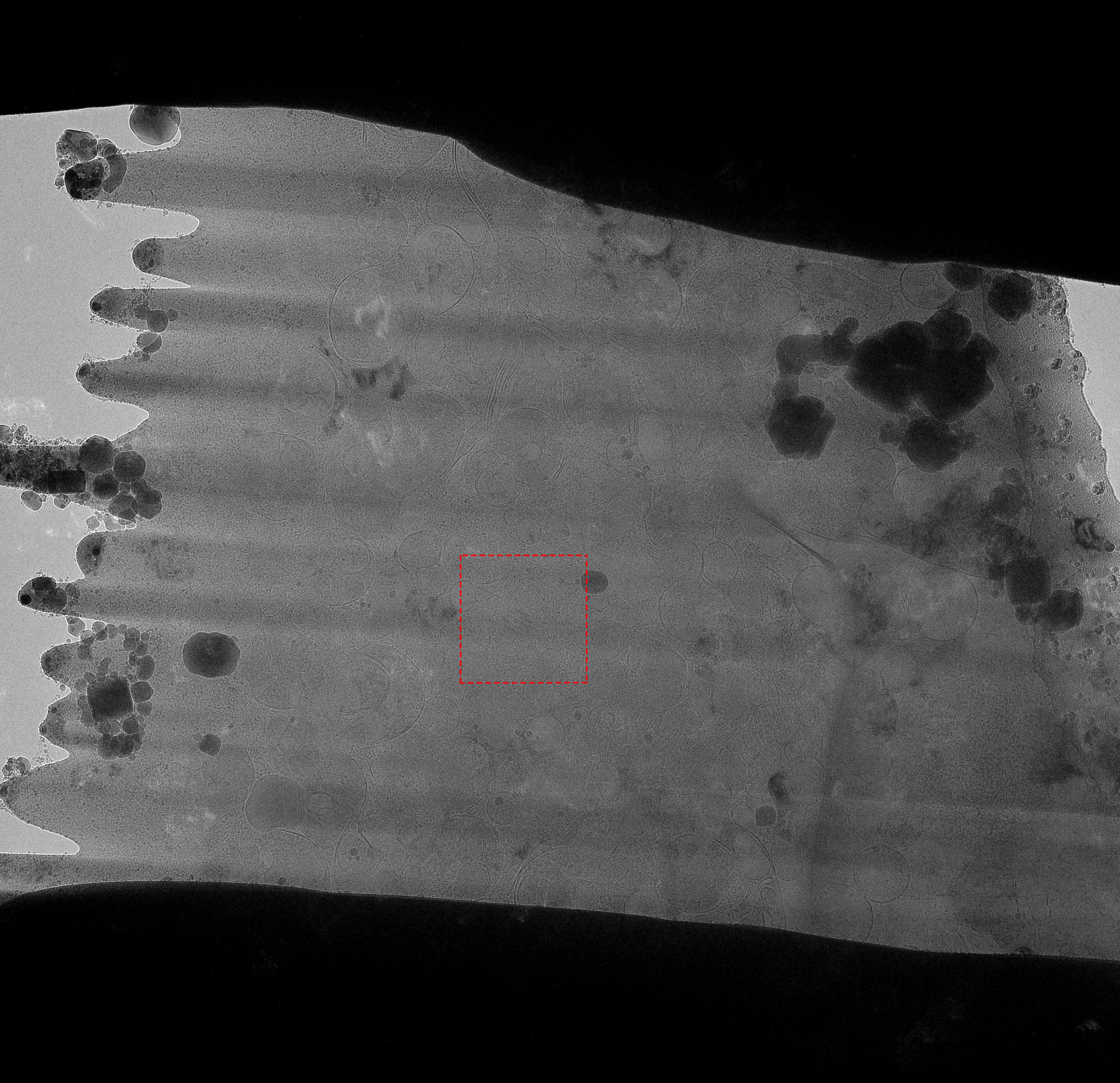

Supplement: Supplementary file 8 — Raw cryo-EM images of all the cryo-lamellae shown in Supplementary Fig. 1. The locations of centrioles are marked by dashed squares. [file 41592_2022_1748_MOESM8_ESM.zip › Supplementary_Data1/Lamella46_Location43.jpg]

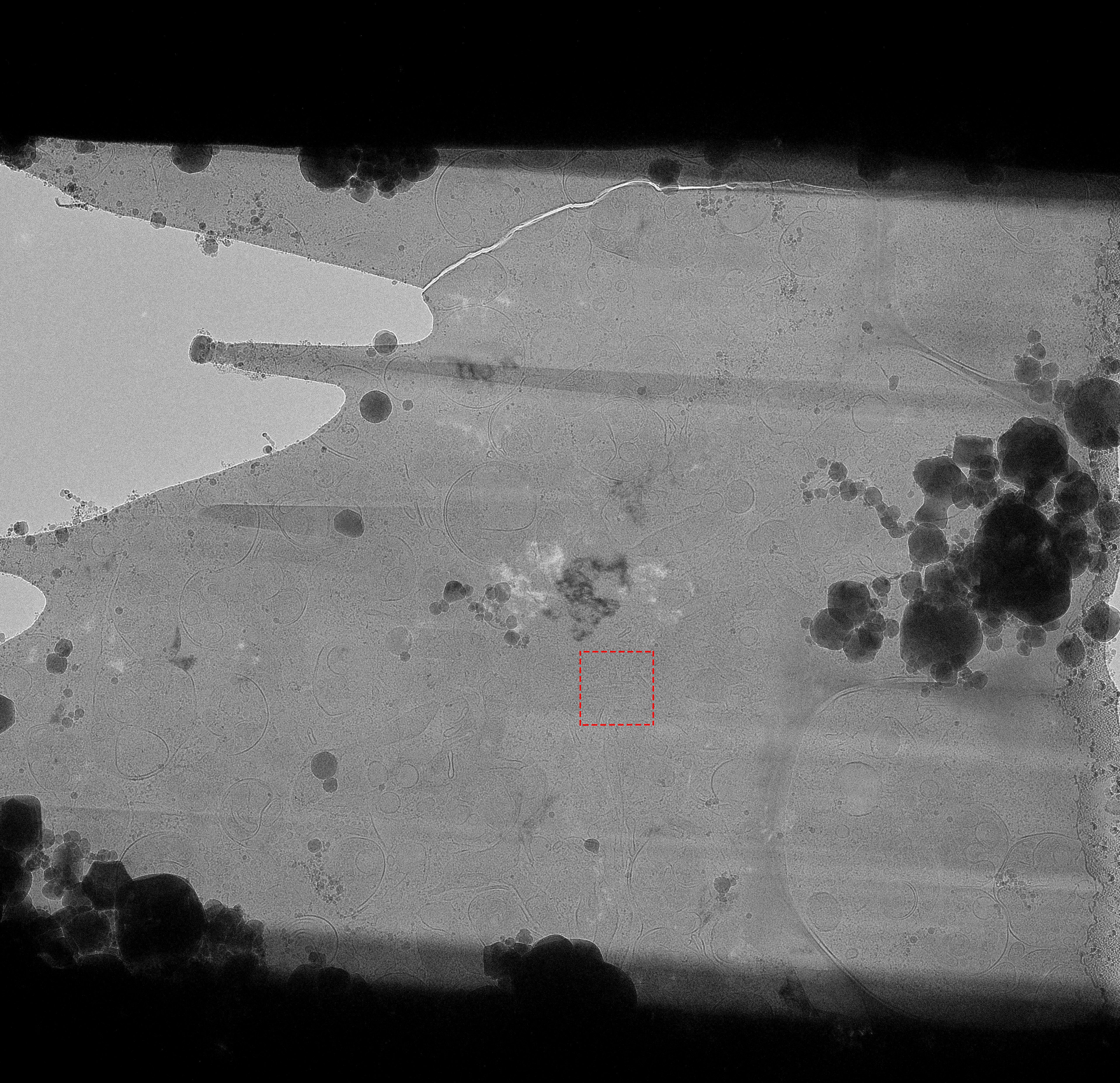

Supplement: Supplementary file 8 — Raw cryo-EM images of all the cryo-lamellae shown in Supplementary Fig. 1. The locations of centrioles are marked by dashed squares. [file 41592_2022_1748_MOESM8_ESM.zip › Supplementary_Data1/Lamella48_Location45.jpg]

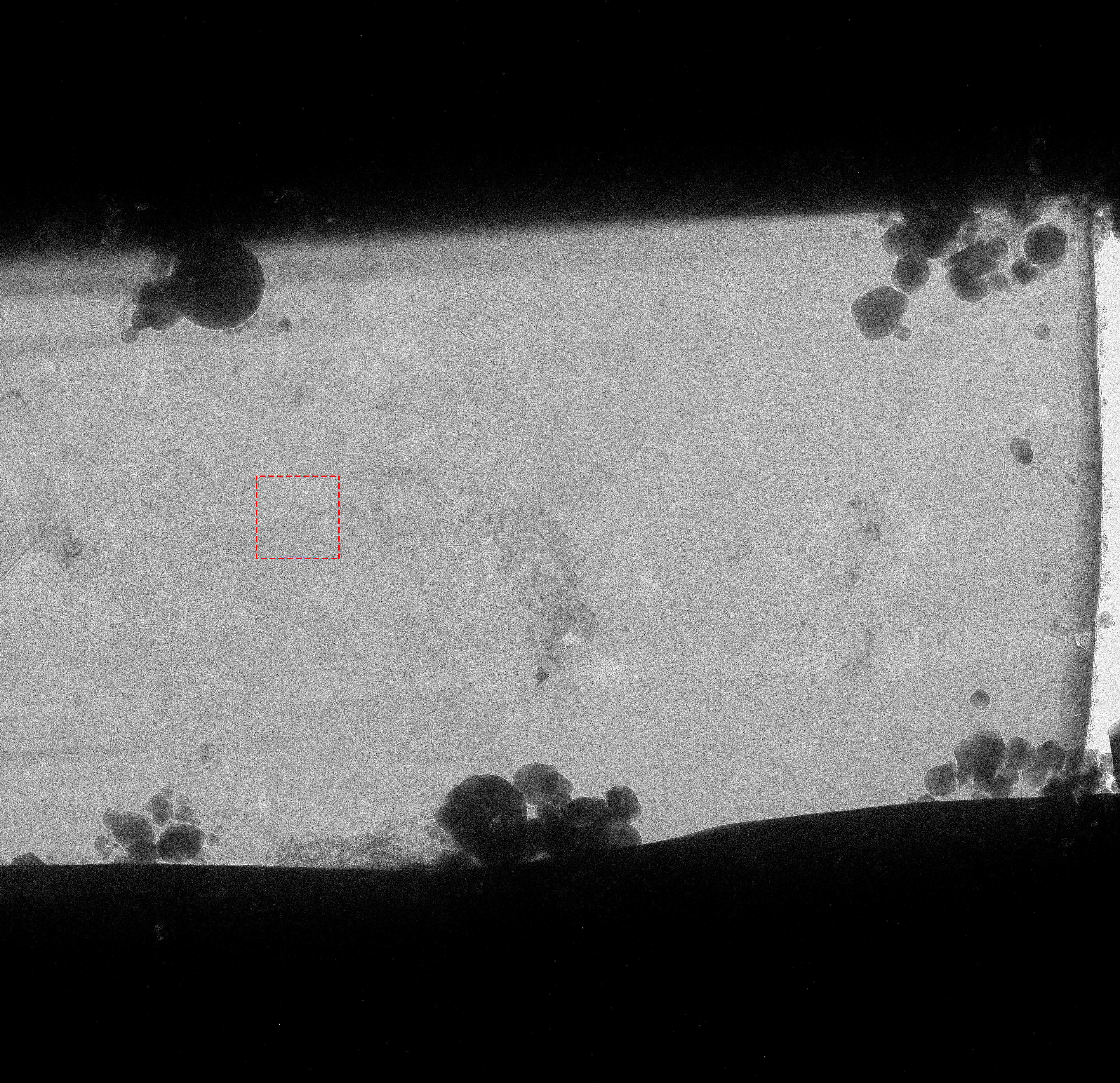

Supplement: Supplementary file 8 — Raw cryo-EM images of all the cryo-lamellae shown in Supplementary Fig. 1. The locations of centrioles are marked by dashed squares. [file 41592_2022_1748_MOESM8_ESM.zip › Supplementary_Data1/Lamella12_Location12.jpg]

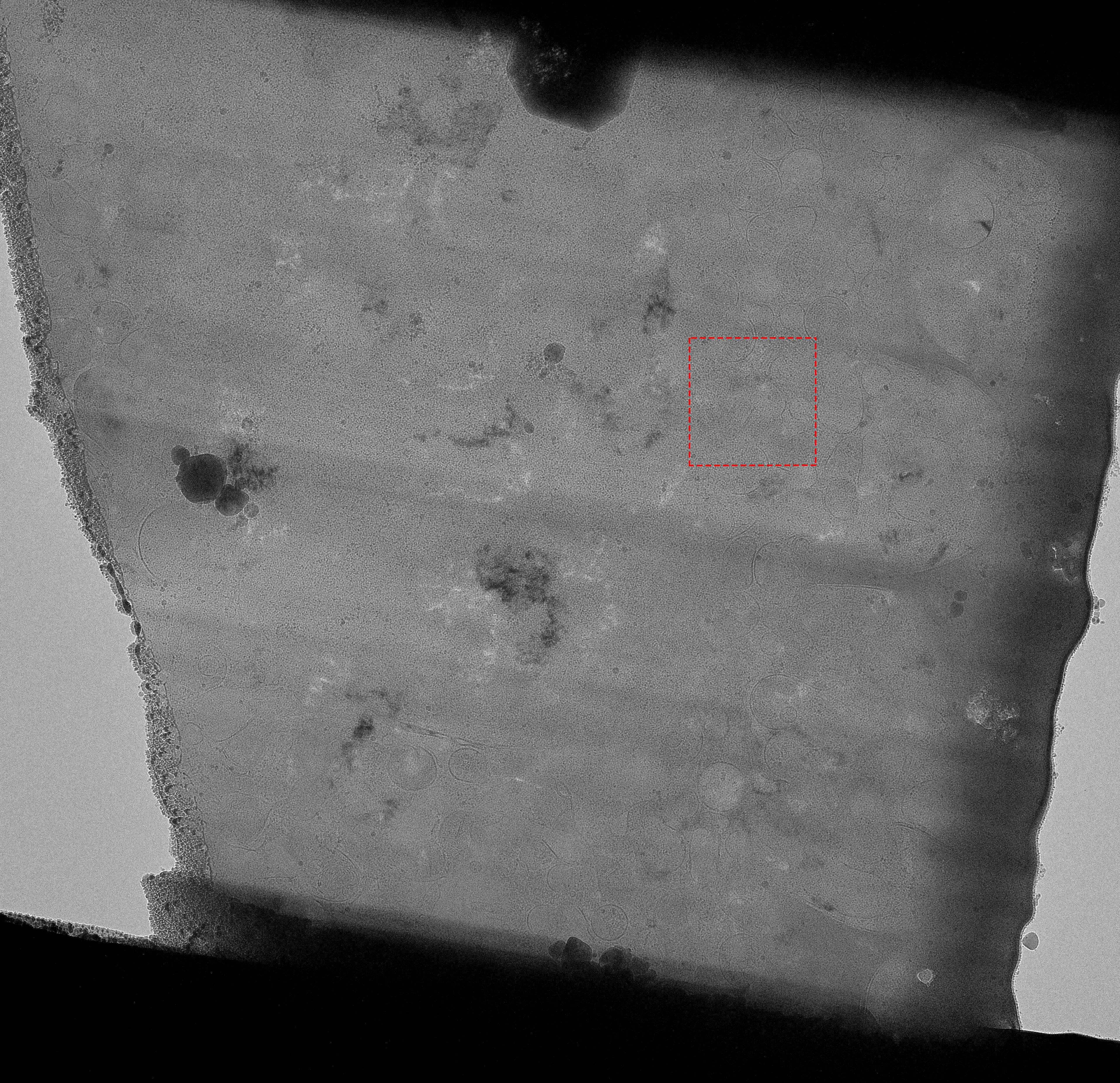

Supplement: Supplementary file 8 — Raw cryo-EM images of all the cryo-lamellae shown in Supplementary Fig. 1. The locations of centrioles are marked by dashed squares. [file 41592_2022_1748_MOESM8_ESM.zip › Supplementary_Data1/Lamella55_Location52.jpg]

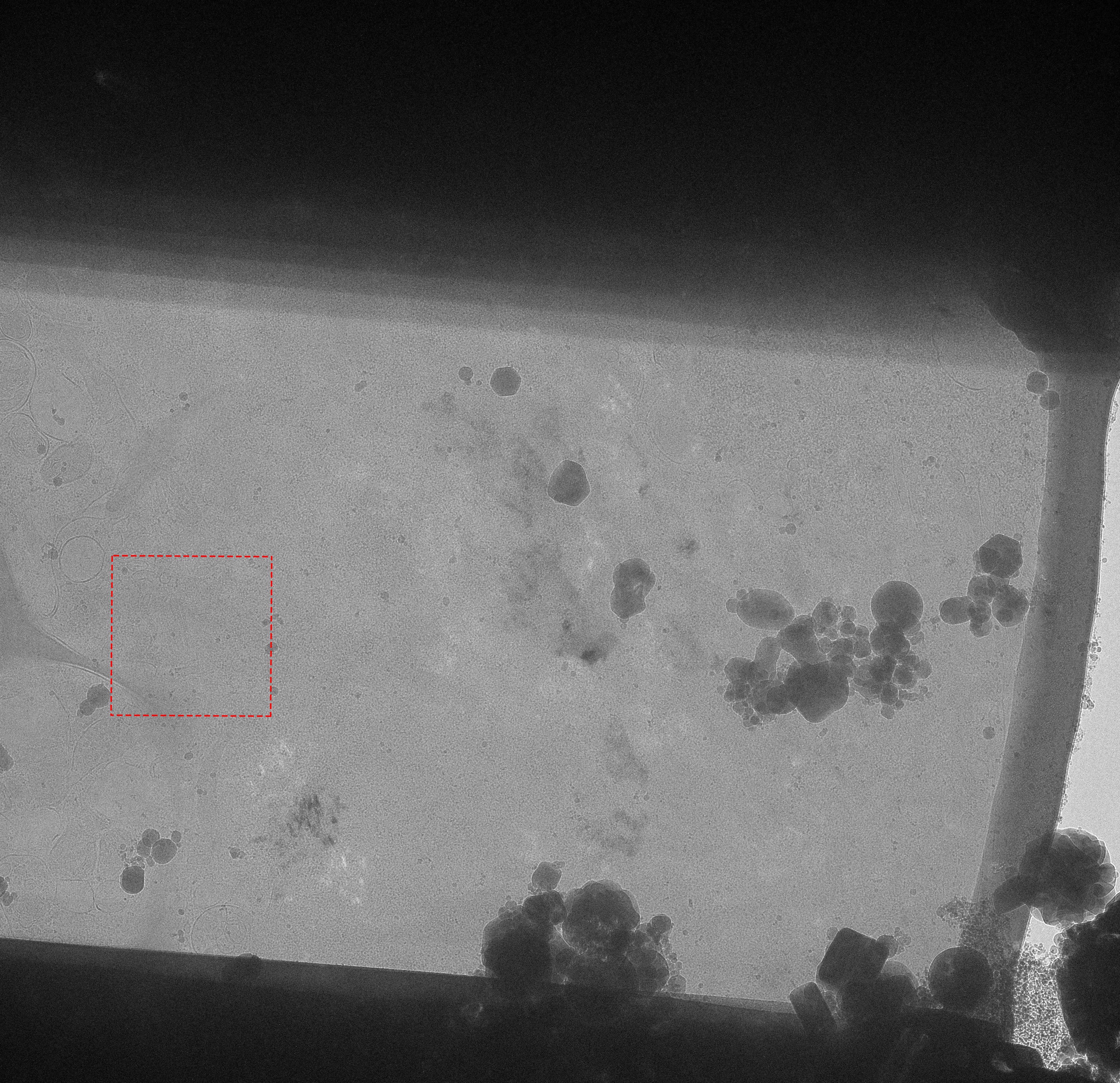

Supplement: Supplementary file 8 — Raw cryo-EM images of all the cryo-lamellae shown in Supplementary Fig. 1. The locations of centrioles are marked by dashed squares. [file 41592_2022_1748_MOESM8_ESM.zip › Supplementary_Data1/Lamella32_Location30.jpg]

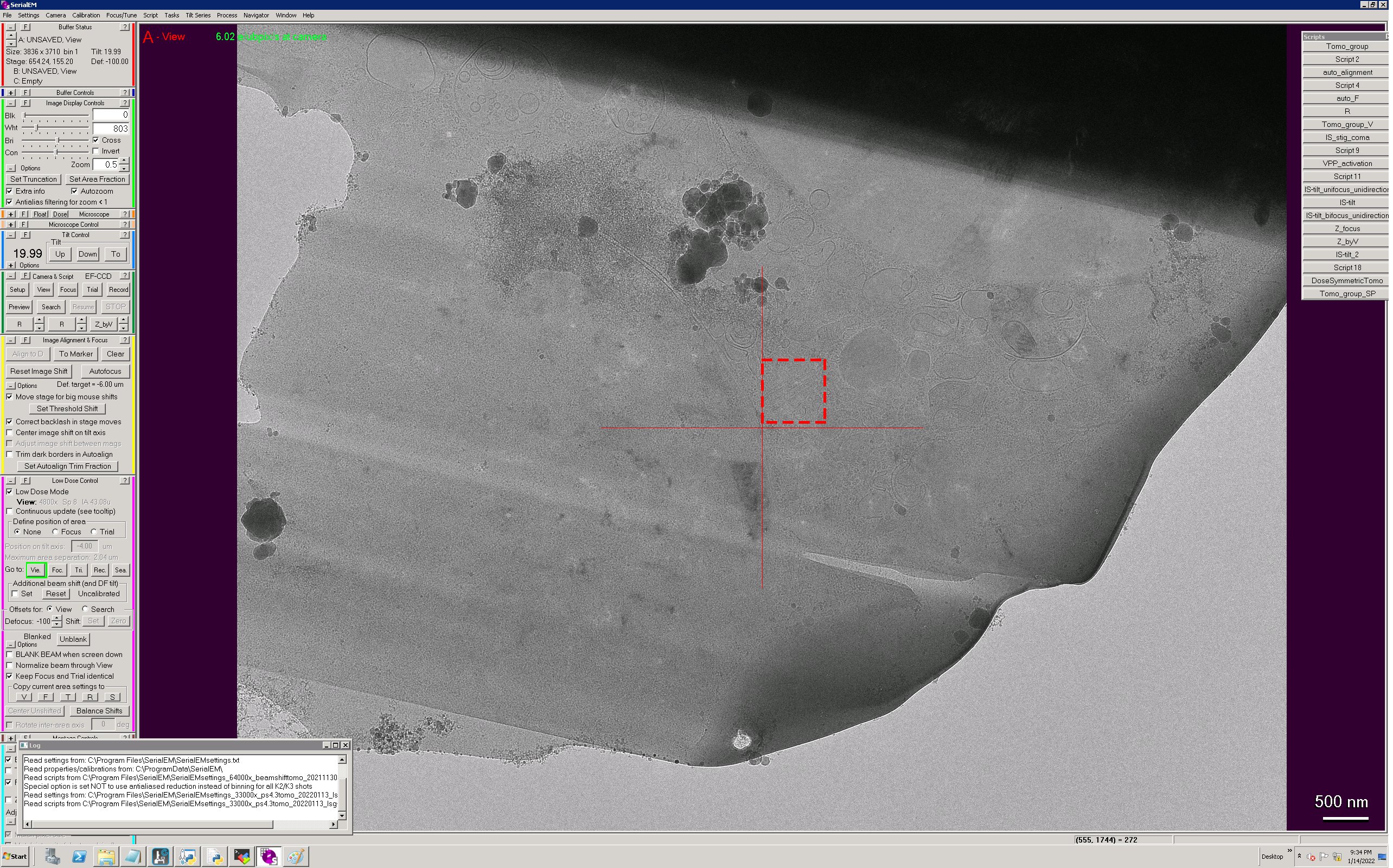

Supplement: Supplementary file 8 — Raw cryo-EM images of all the cryo-lamellae shown in Supplementary Fig. 1. The locations of centrioles are marked by dashed squares. [file 41592_2022_1748_MOESM8_ESM.zip › Supplementary_Data1/Lamella72_Location65.jpg]

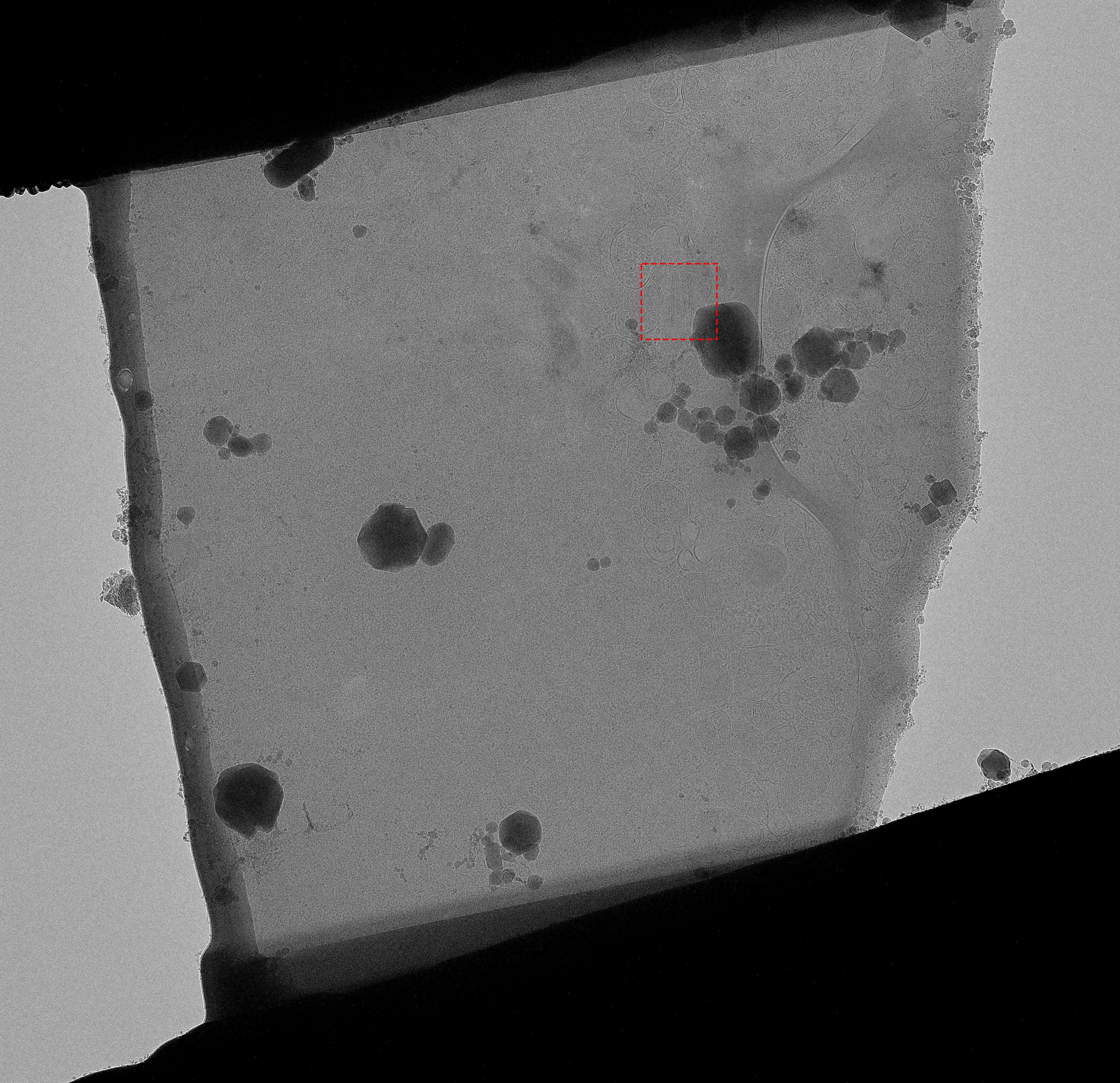

Supplement: Supplementary file 8 — Raw cryo-EM images of all the cryo-lamellae shown in Supplementary Fig. 1. The locations of centrioles are marked by dashed squares. [file 41592_2022_1748_MOESM8_ESM.zip › Supplementary_Data1/Lamella35_Location33.jpg]

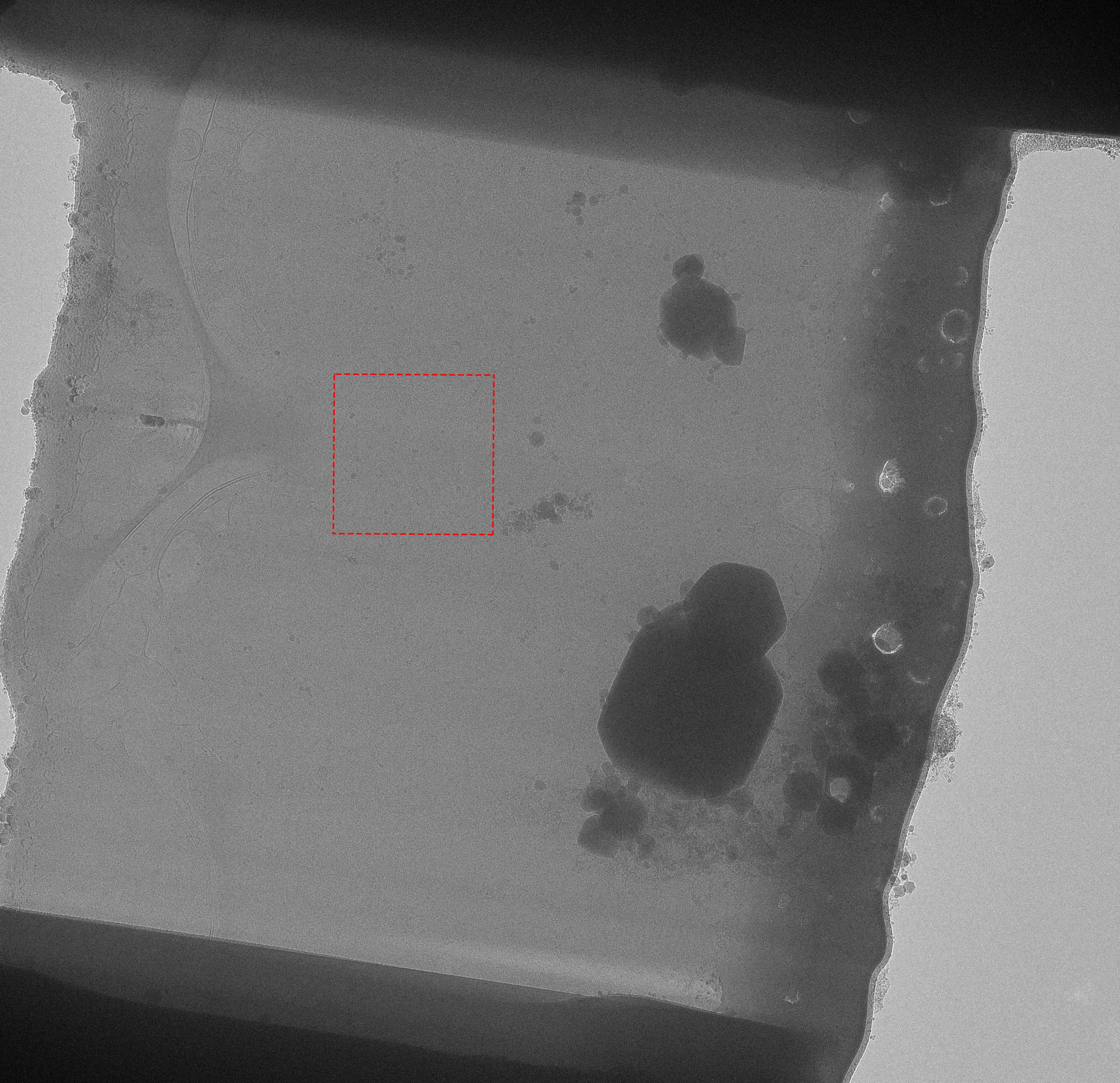

Supplement: Supplementary file 8 — Raw cryo-EM images of all the cryo-lamellae shown in Supplementary Fig. 1. The locations of centrioles are marked by dashed squares. [file 41592_2022_1748_MOESM8_ESM.zip › Supplementary_Data1/Lamella28_Location27.jpg]

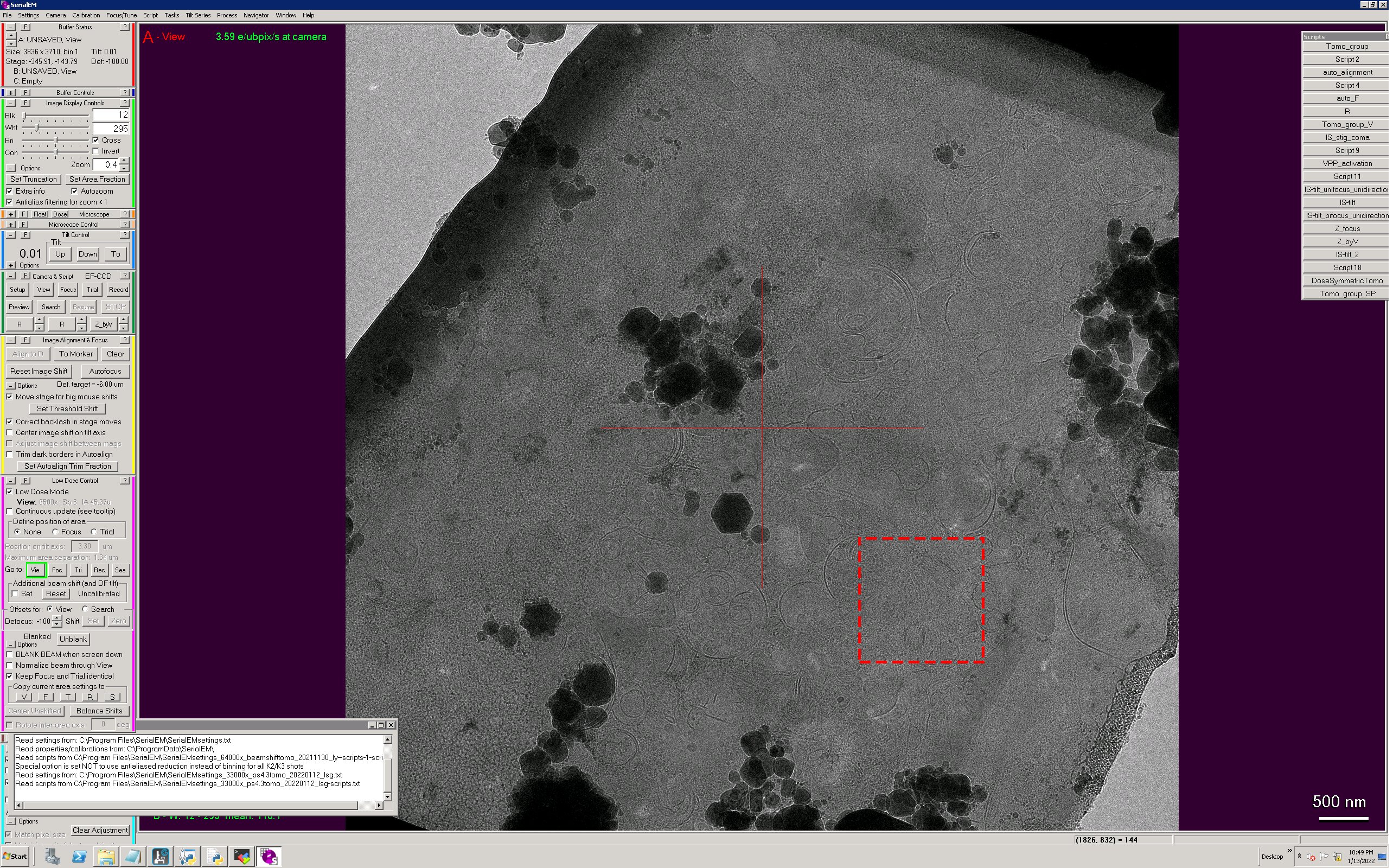

Supplement: Supplementary file 8 — Raw cryo-EM images of all the cryo-lamellae shown in Supplementary Fig. 1. The locations of centrioles are marked by dashed squares. [file 41592_2022_1748_MOESM8_ESM.zip › Supplementary_Data1/Lamella66_Location60.jpg]

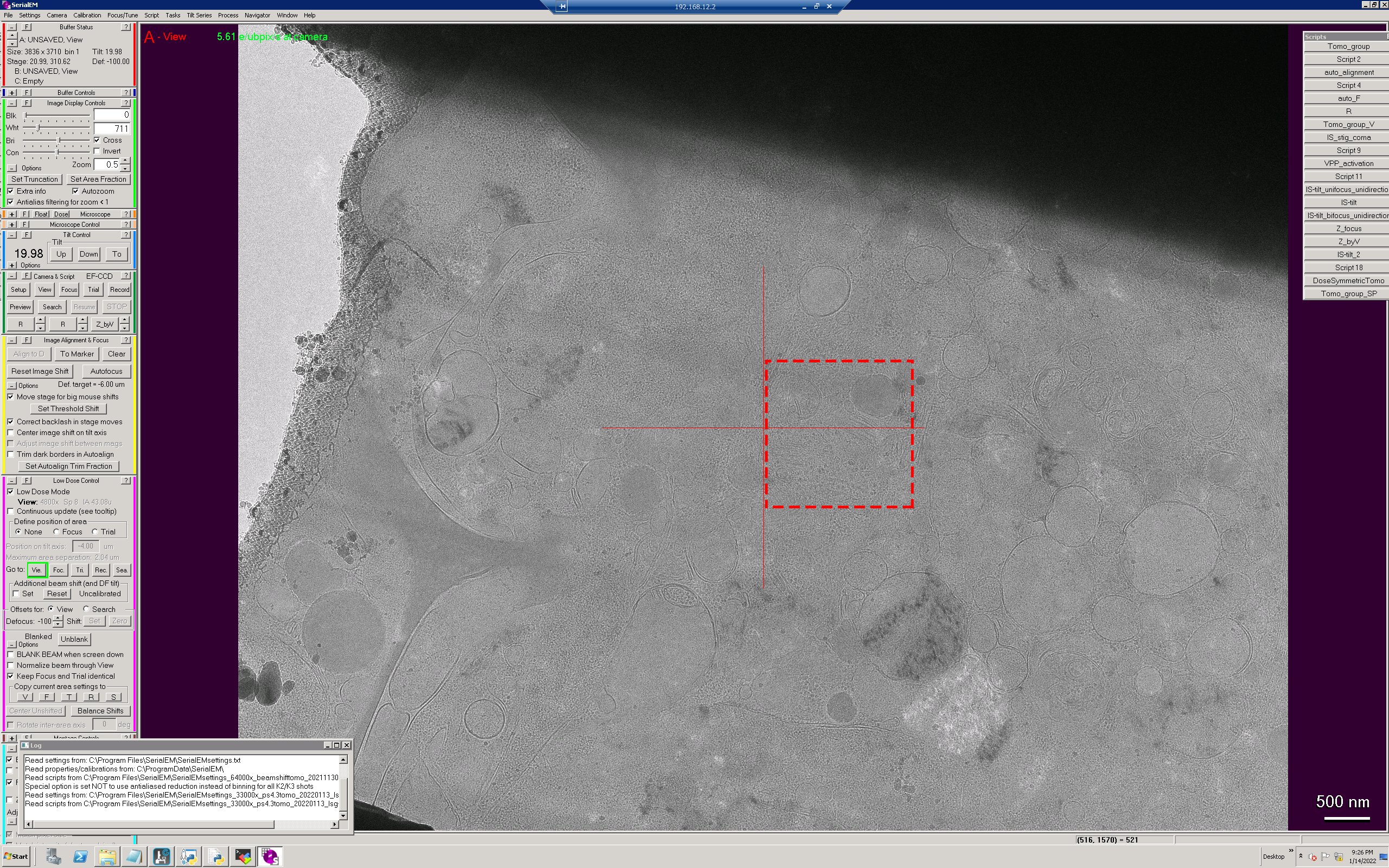

Supplement: Supplementary file 8 — Raw cryo-EM images of all the cryo-lamellae shown in Supplementary Fig. 1. The locations of centrioles are marked by dashed squares. [file 41592_2022_1748_MOESM8_ESM.zip › Supplementary_Data1/Lamella69_Location62.jpg]

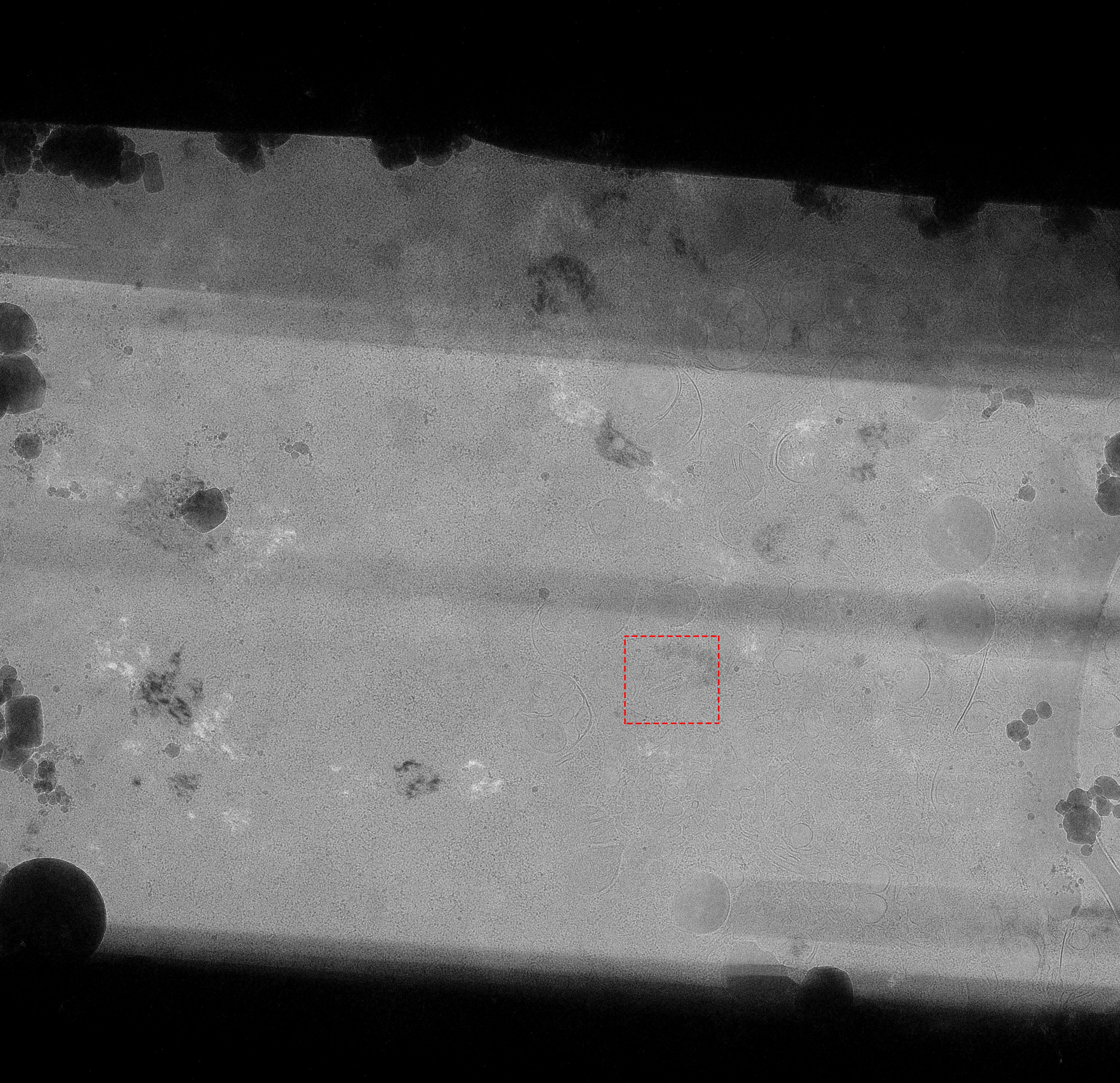

Supplement: Supplementary file 8 — Raw cryo-EM images of all the cryo-lamellae shown in Supplementary Fig. 1. The locations of centrioles are marked by dashed squares. [file 41592_2022_1748_MOESM8_ESM.zip › Supplementary_Data1/Lamella47_Location44.jpg]

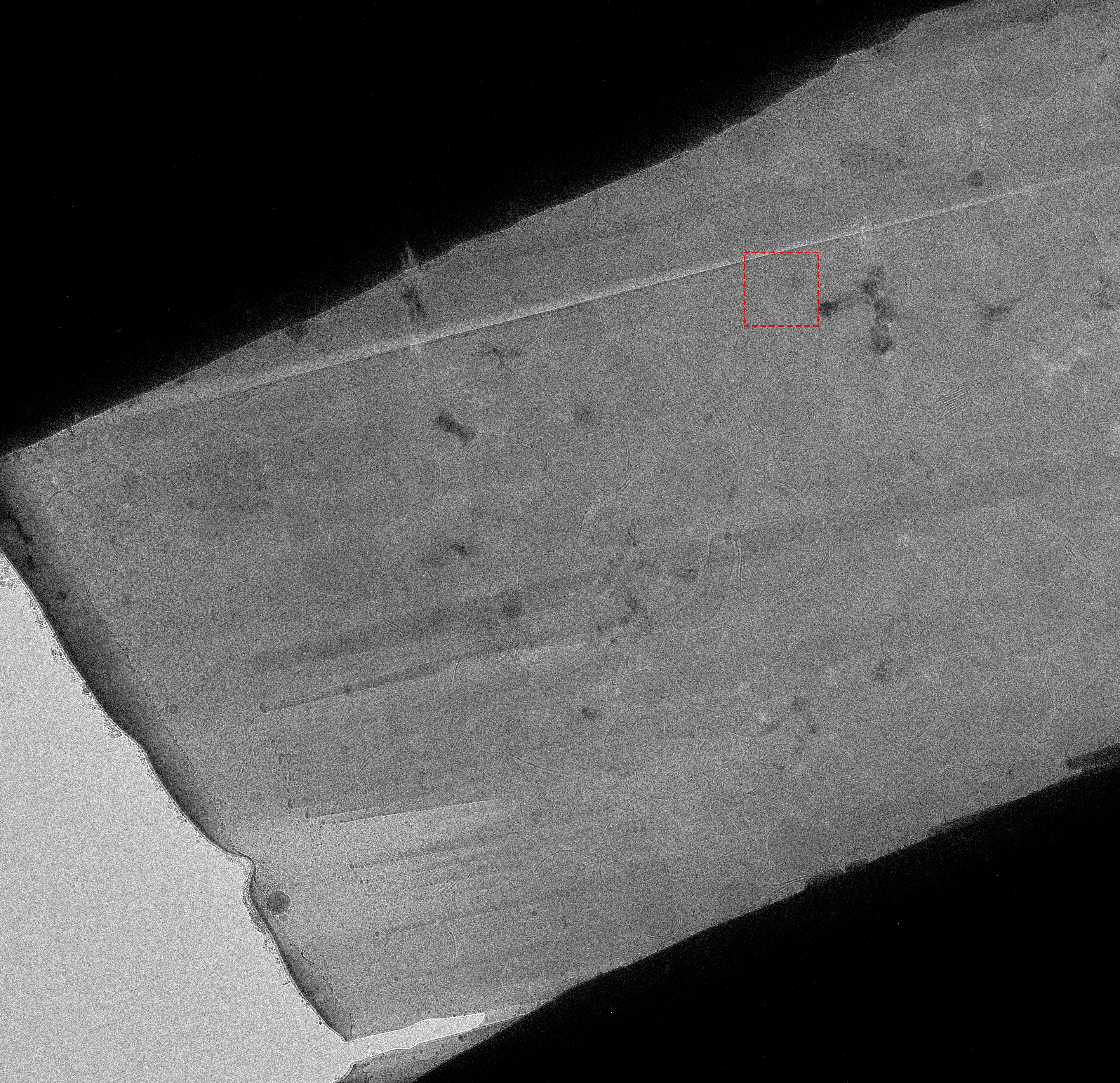

Supplement: Supplementary file 8 — Raw cryo-EM images of all the cryo-lamellae shown in Supplementary Fig. 1. The locations of centrioles are marked by dashed squares. [file 41592_2022_1748_MOESM8_ESM.zip › Supplementary_Data1/Lamella33_Location31.jpg]

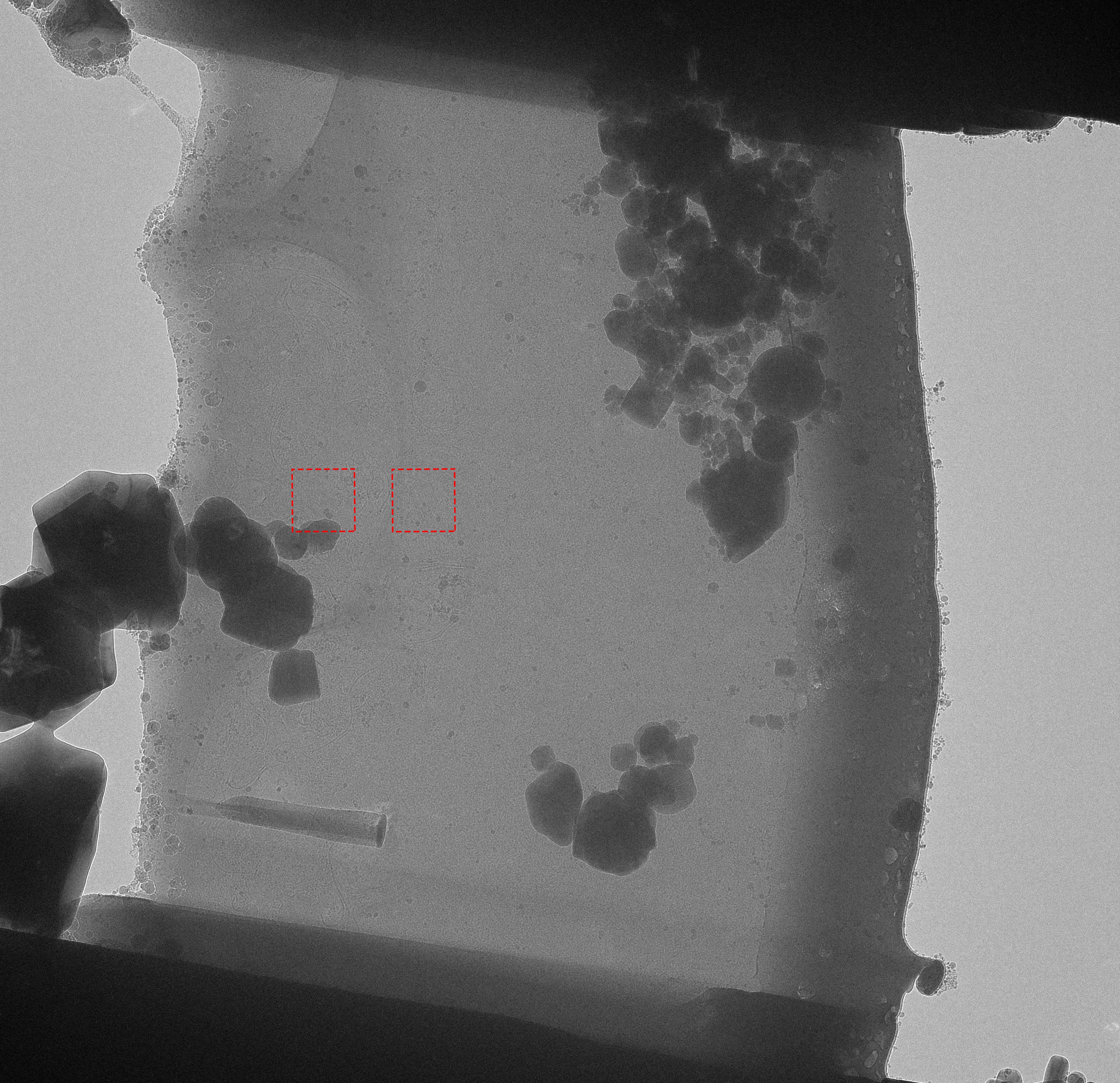

Supplement: Supplementary file 8 — Raw cryo-EM images of all the cryo-lamellae shown in Supplementary Fig. 1. The locations of centrioles are marked by dashed squares. [file 41592_2022_1748_MOESM8_ESM.zip › Supplementary_Data1/Lamella26_Location25.jpg]

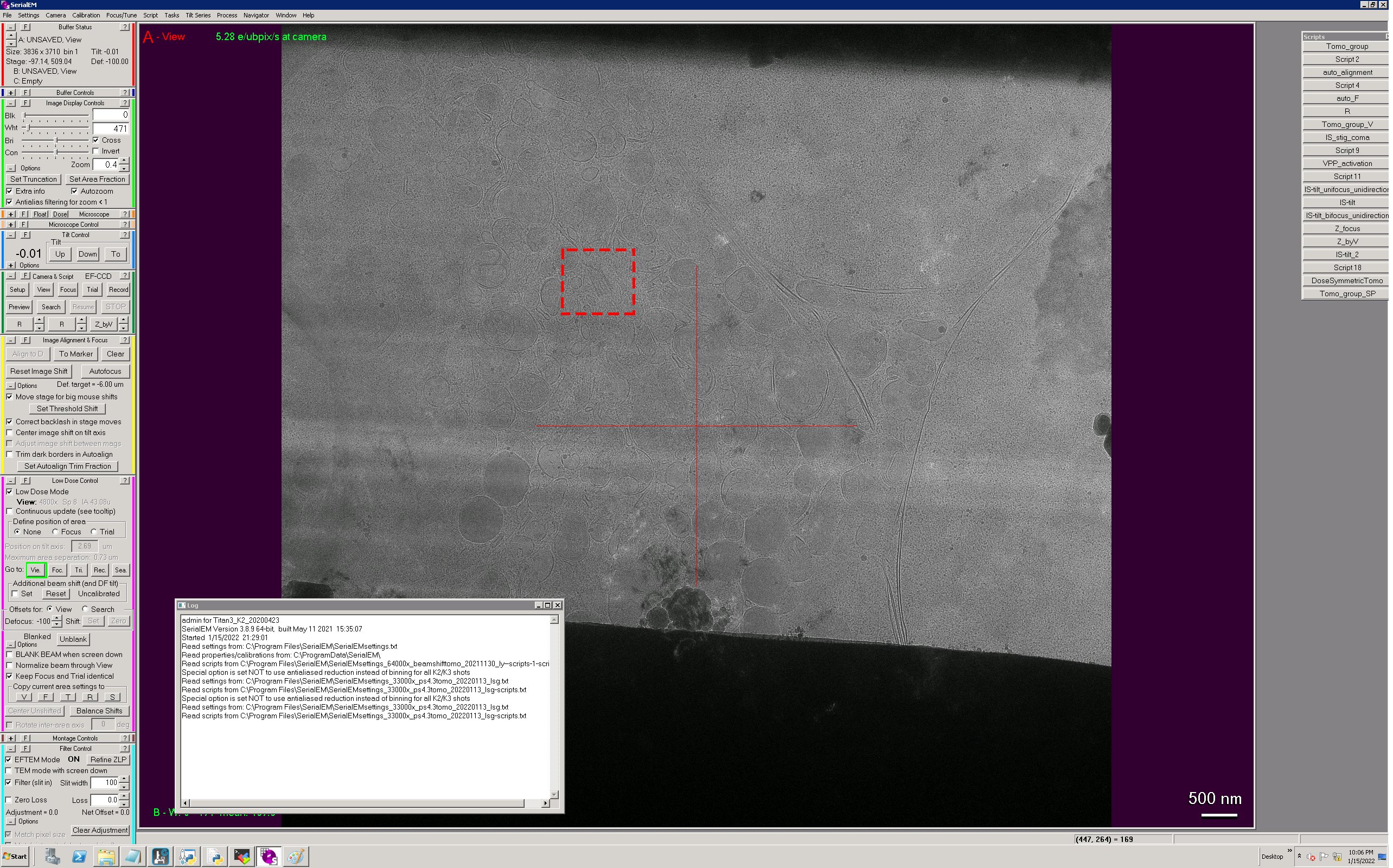

Supplement: Supplementary file 8 — Raw cryo-EM images of all the cryo-lamellae shown in Supplementary Fig. 1. The locations of centrioles are marked by dashed squares. [file 41592_2022_1748_MOESM8_ESM.zip › Supplementary_Data1/Lamella74_Location67.jpg]

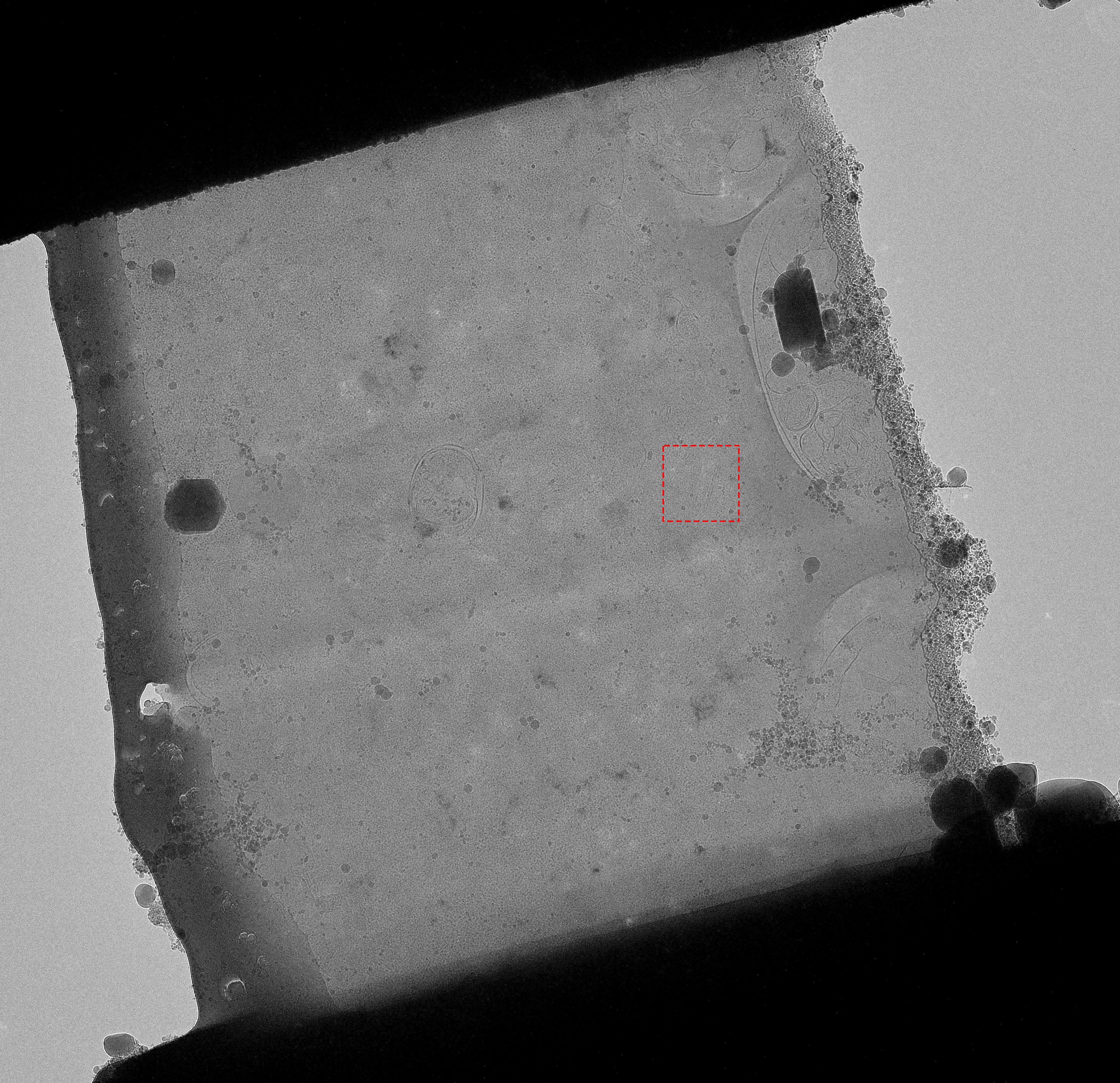

Supplement: Supplementary file 8 — Raw cryo-EM images of all the cryo-lamellae shown in Supplementary Fig. 1. The locations of centrioles are marked by dashed squares. [file 41592_2022_1748_MOESM8_ESM.zip › Supplementary_Data1/Lamella34_Location32.jpg]

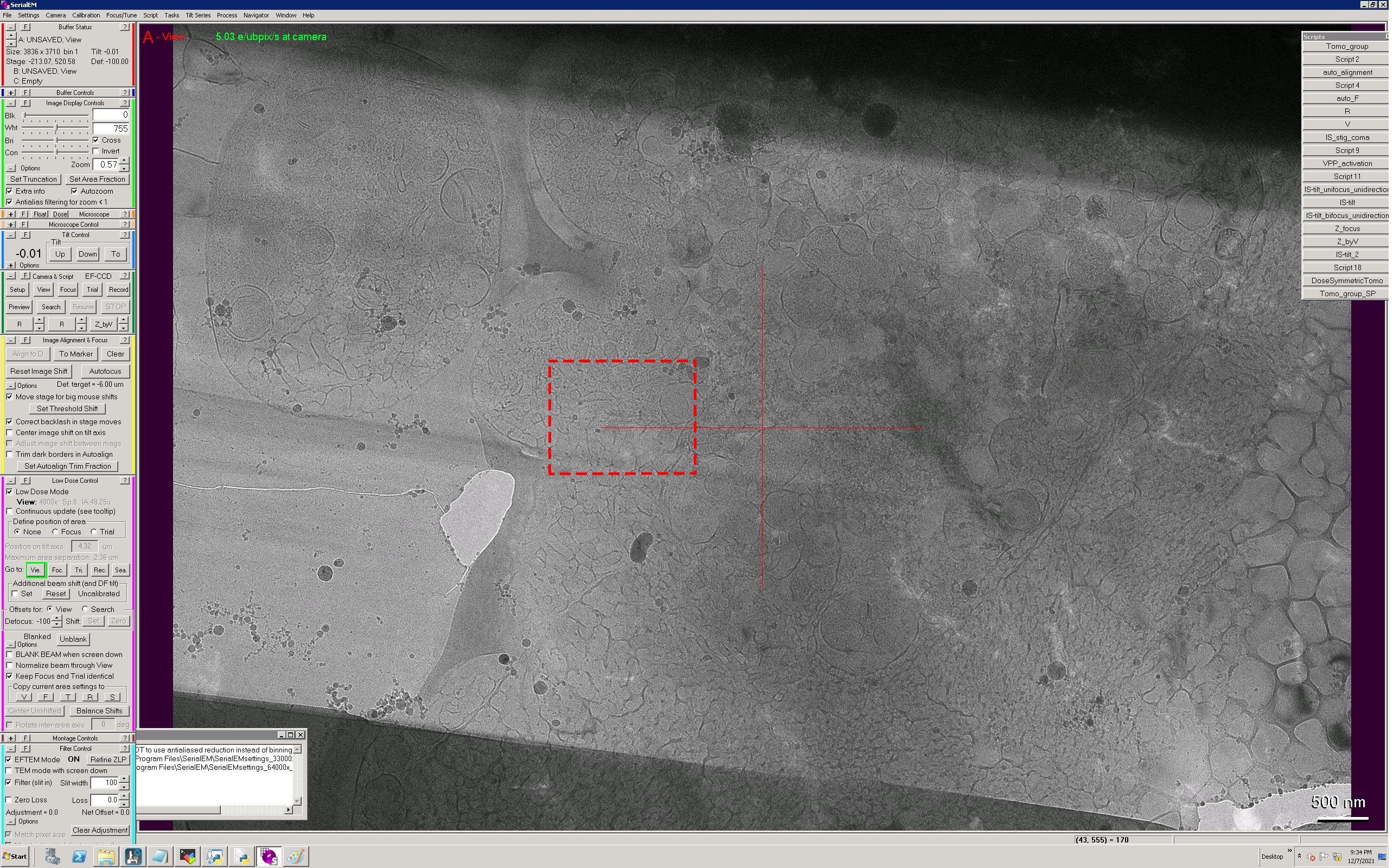

Supplement: Supplementary file 8 — Raw cryo-EM images of all the cryo-lamellae shown in Supplementary Fig. 1. The locations of centrioles are marked by dashed squares. [file 41592_2022_1748_MOESM8_ESM.zip › Supplementary_Data1/Lamella53_Location50.jpg]

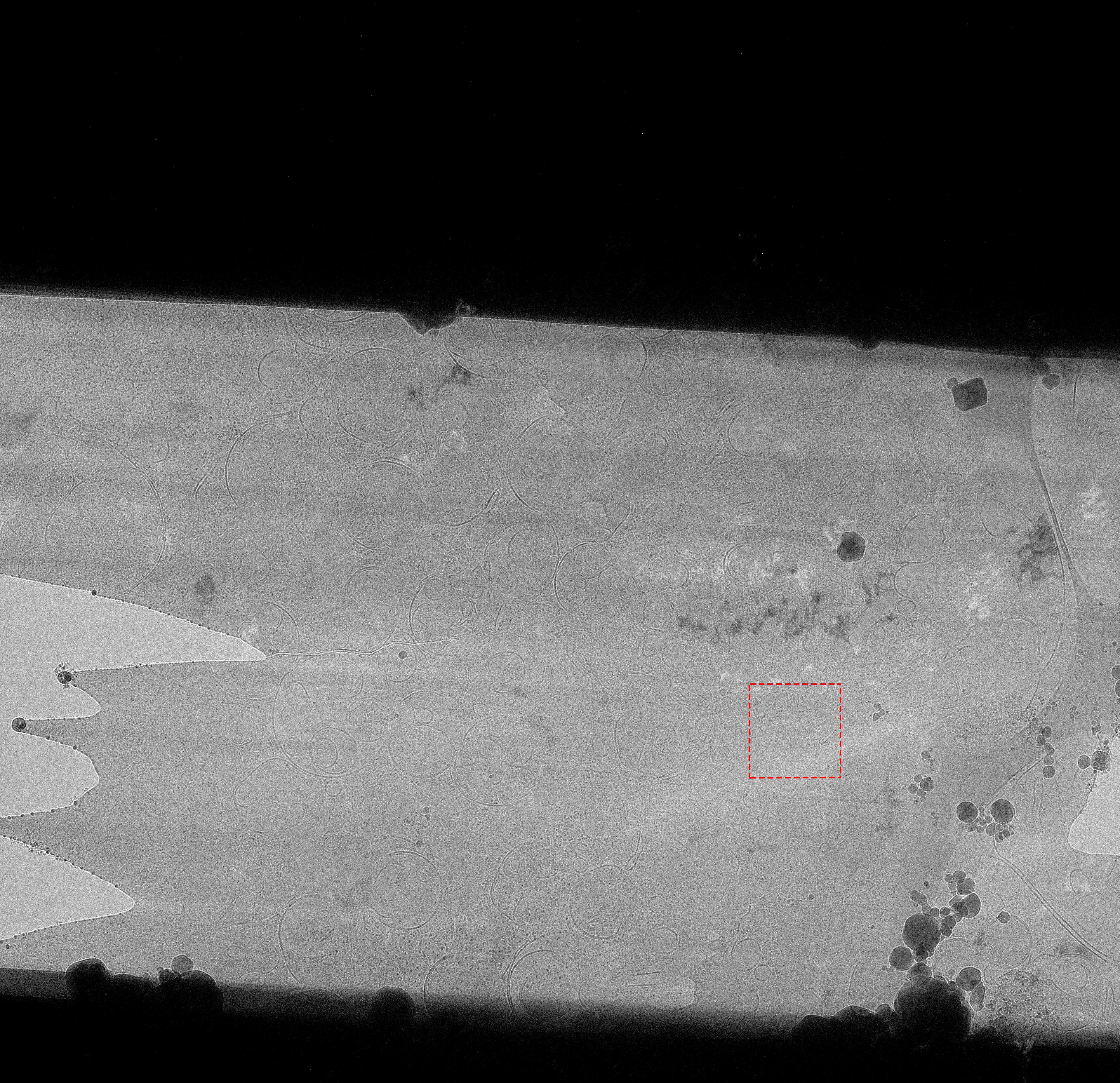

Supplement: Supplementary file 8 — Raw cryo-EM images of all the cryo-lamellae shown in Supplementary Fig. 1. The locations of centrioles are marked by dashed squares. [file 41592_2022_1748_MOESM8_ESM.zip › Supplementary_Data1/Lamella49_Location46.jpg]

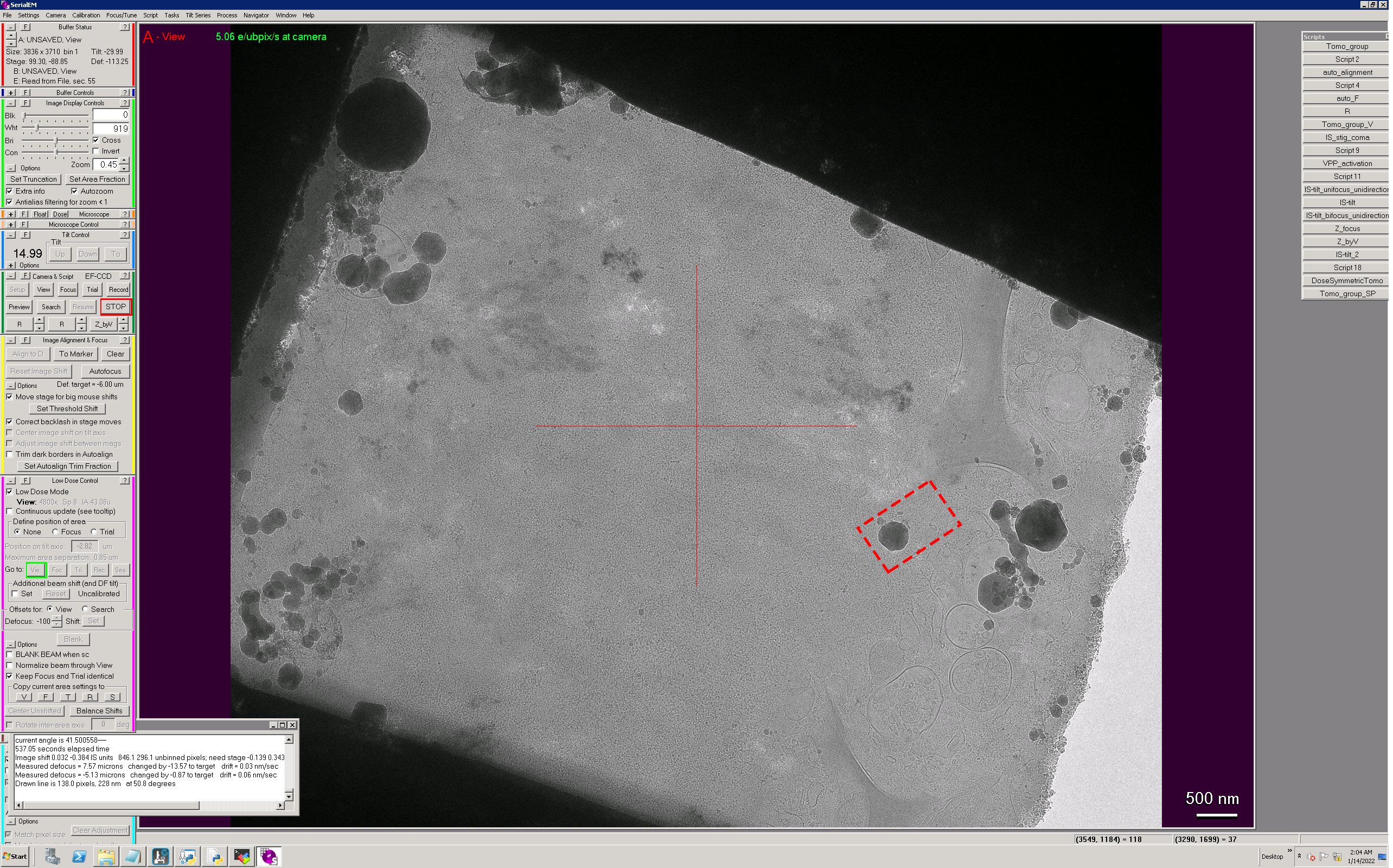

Supplement: Supplementary file 8 — Raw cryo-EM images of all the cryo-lamellae shown in Supplementary Fig. 1. The locations of centrioles are marked by dashed squares. [file 41592_2022_1748_MOESM8_ESM.zip › Supplementary_Data1/Lamella67_Location61.jpg]

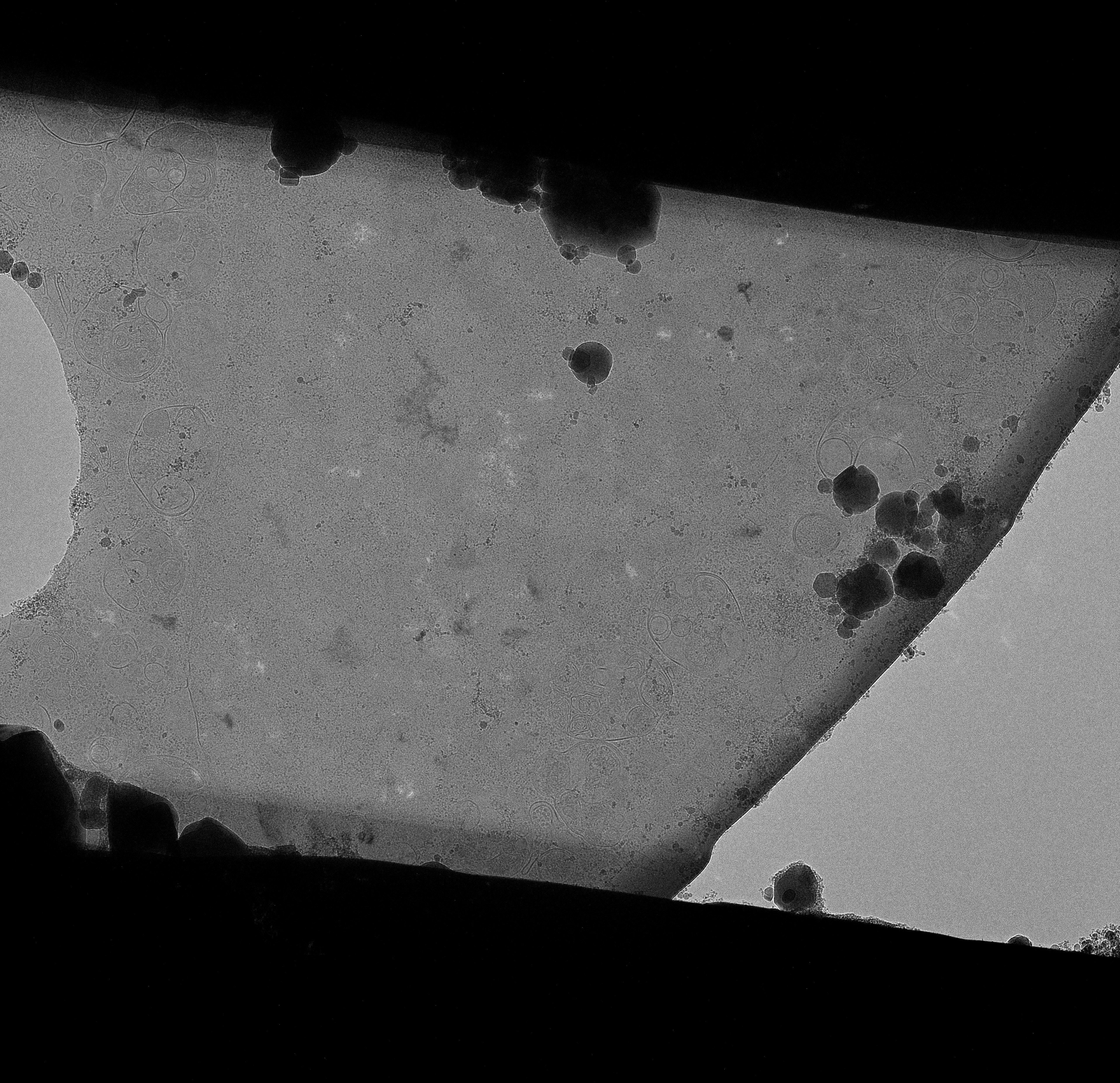

Supplement: Supplementary file 8 — Raw cryo-EM images of all the cryo-lamellae shown in Supplementary Fig. 1. The locations of centrioles are marked by dashed squares. [file 41592_2022_1748_MOESM8_ESM.zip › Supplementary_Data1/Lamella16_NoLocation.jpg]

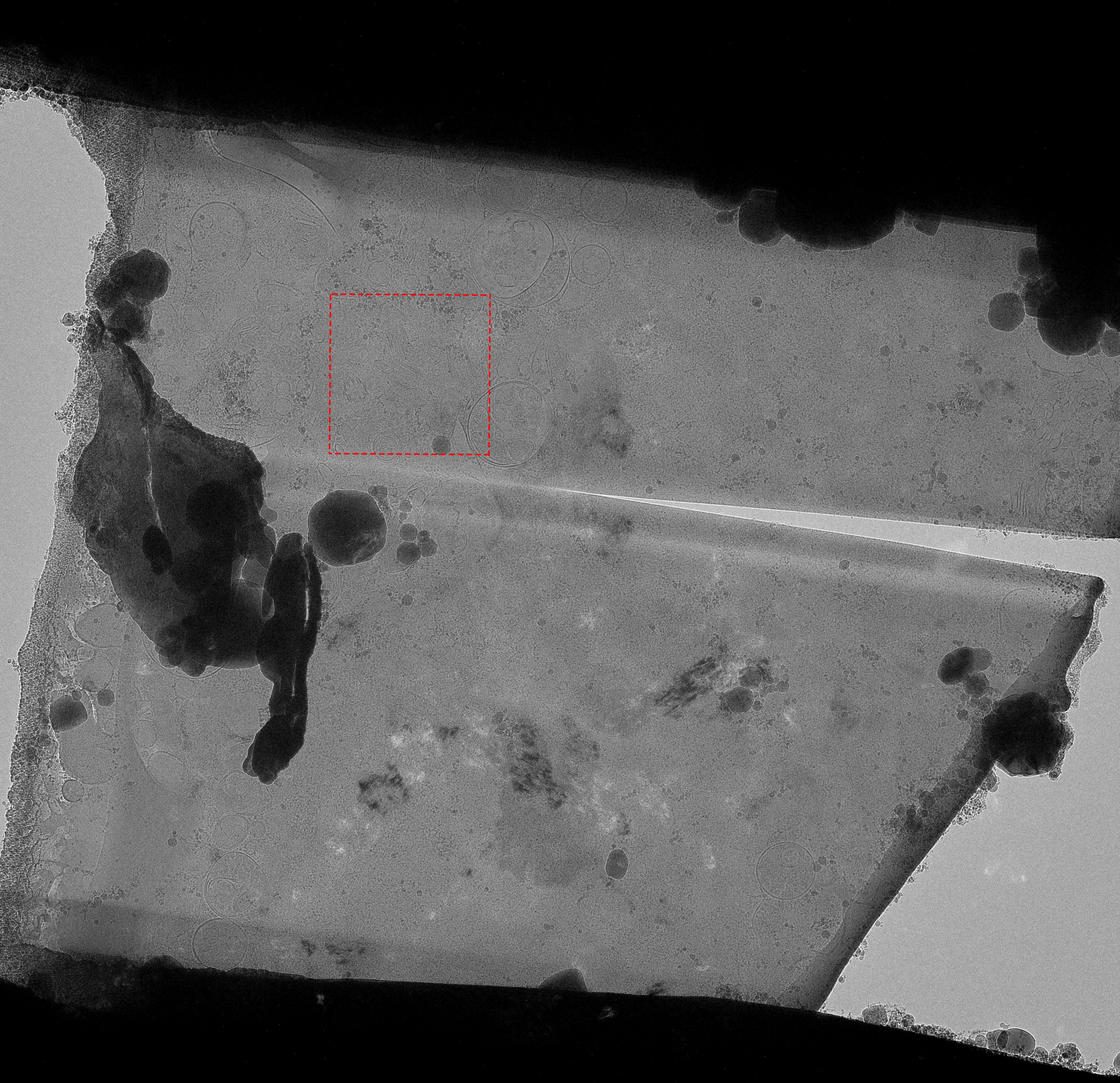

Supplement: Supplementary file 8 — Raw cryo-EM images of all the cryo-lamellae shown in Supplementary Fig. 1. The locations of centrioles are marked by dashed squares. [file 41592_2022_1748_MOESM8_ESM.zip › Supplementary_Data1/Lamella15_Location15.jpg]

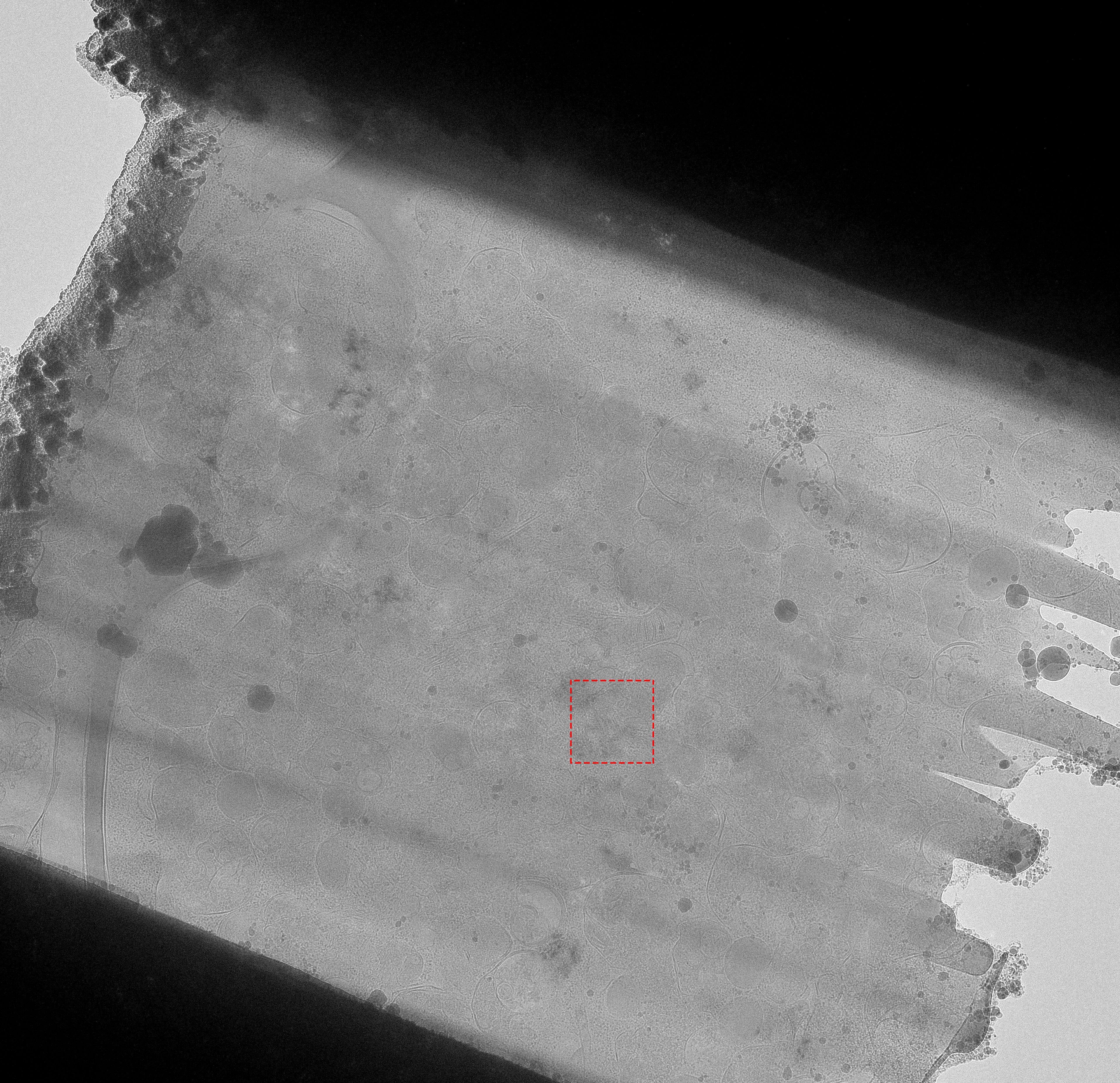

Supplement: Supplementary file 8 — Raw cryo-EM images of all the cryo-lamellae shown in Supplementary Fig. 1. The locations of centrioles are marked by dashed squares. [file 41592_2022_1748_MOESM8_ESM.zip › Supplementary_Data1/Lamella06_Location06.jpg]

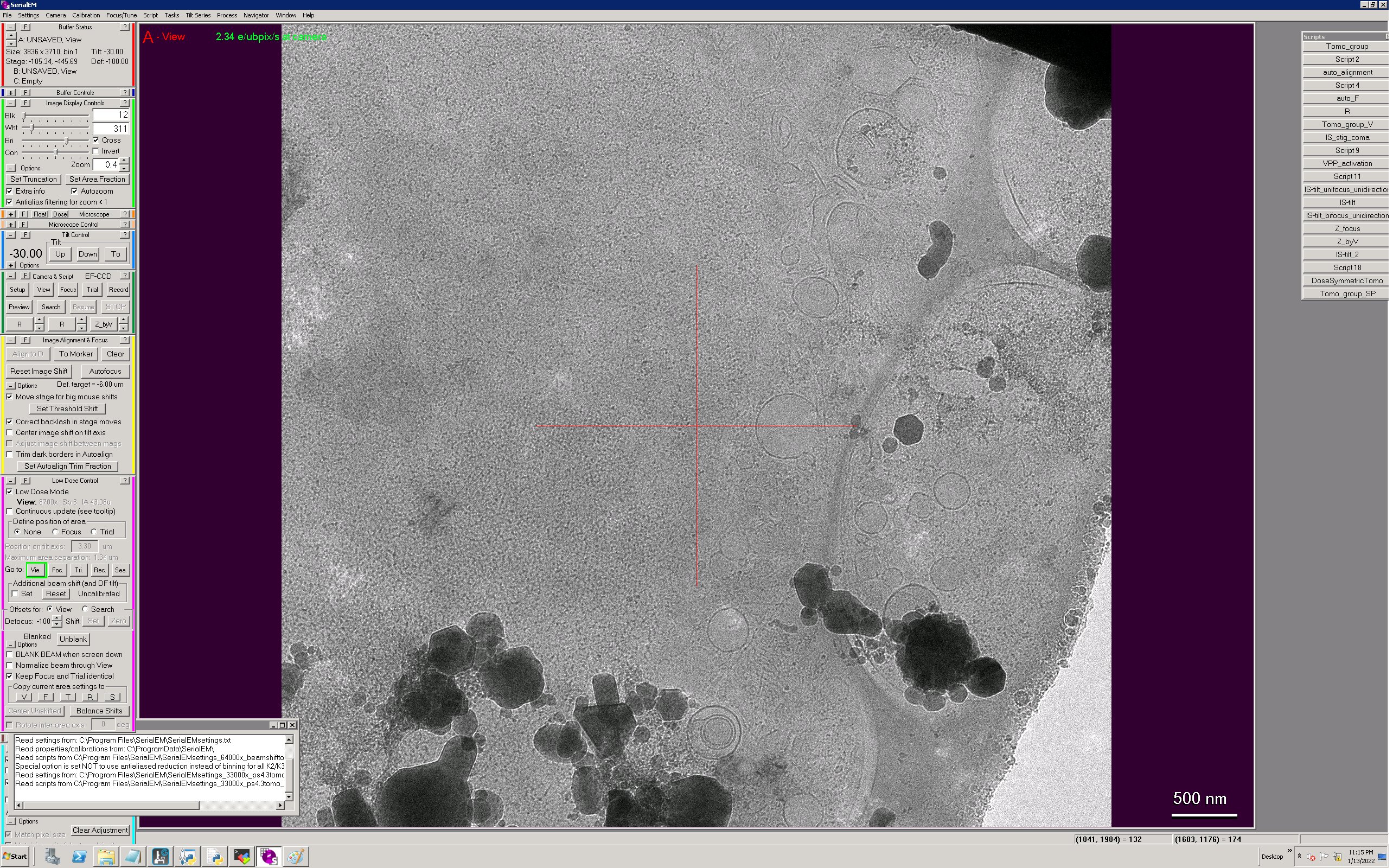

Supplement: Supplementary file 8 — Raw cryo-EM images of all the cryo-lamellae shown in Supplementary Fig. 1. The locations of centrioles are marked by dashed squares. [file 41592_2022_1748_MOESM8_ESM.zip › Supplementary_Data1/Lamella68_NoLocation.jpg]

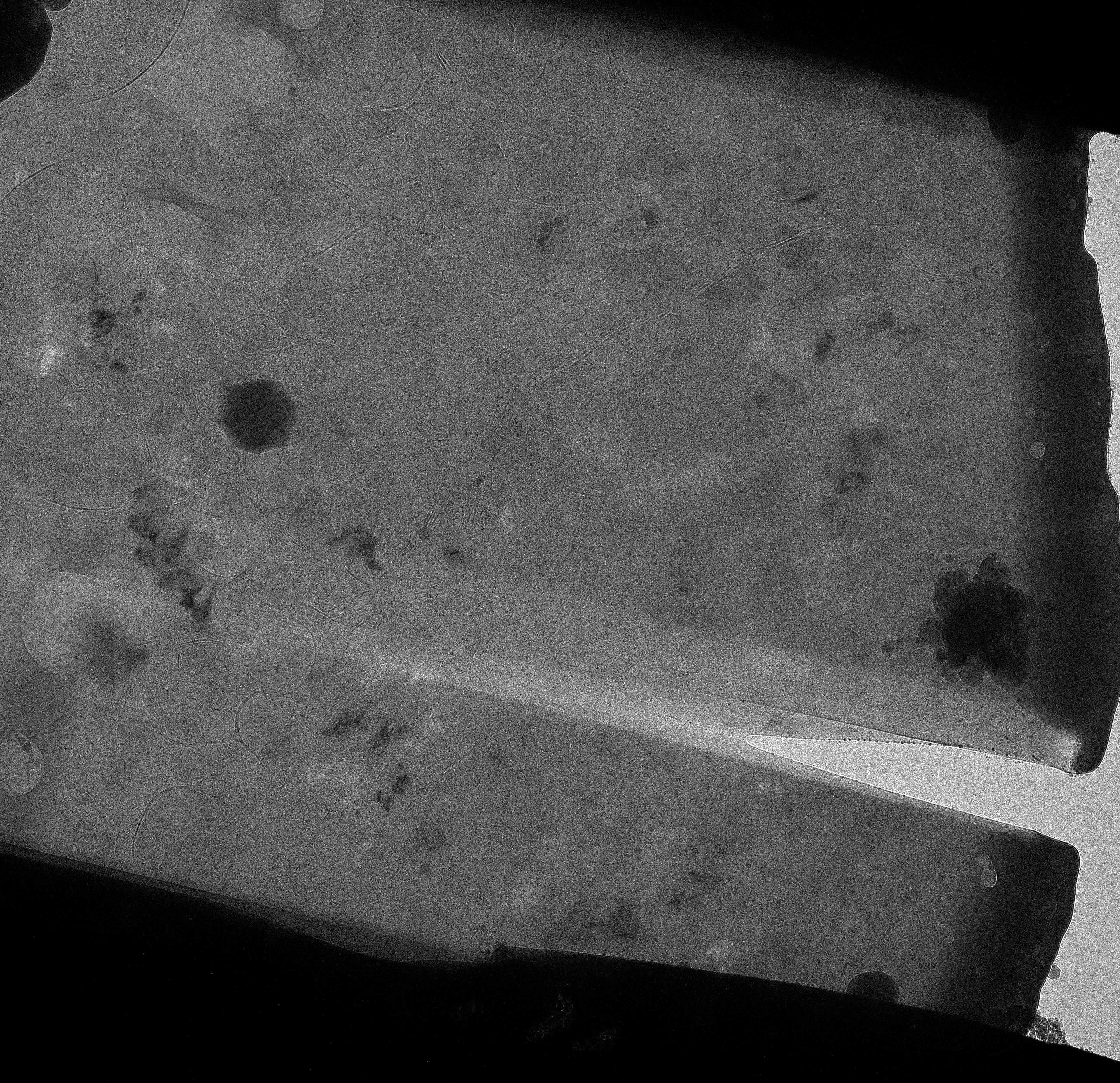

Supplement: Supplementary file 8 — Raw cryo-EM images of all the cryo-lamellae shown in Supplementary Fig. 1. The locations of centrioles are marked by dashed squares. [file 41592_2022_1748_MOESM8_ESM.zip › Supplementary_Data1/Lamella60_NoLocation.jpg]

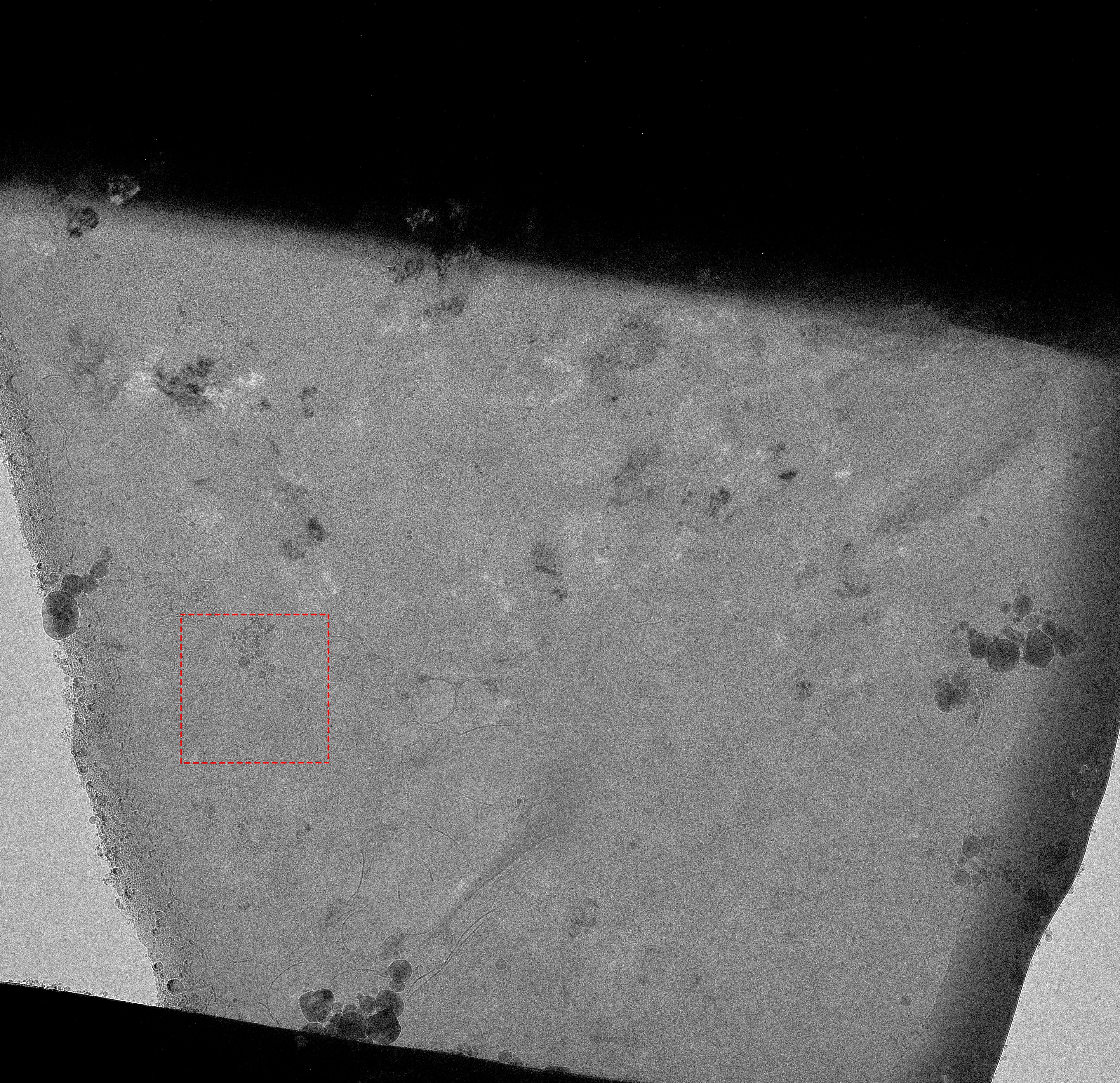

Supplement: Supplementary file 8 — Raw cryo-EM images of all the cryo-lamellae shown in Supplementary Fig. 1. The locations of centrioles are marked by dashed squares. [file 41592_2022_1748_MOESM8_ESM.zip › Supplementary_Data1/Lamella54_Location51.jpg]

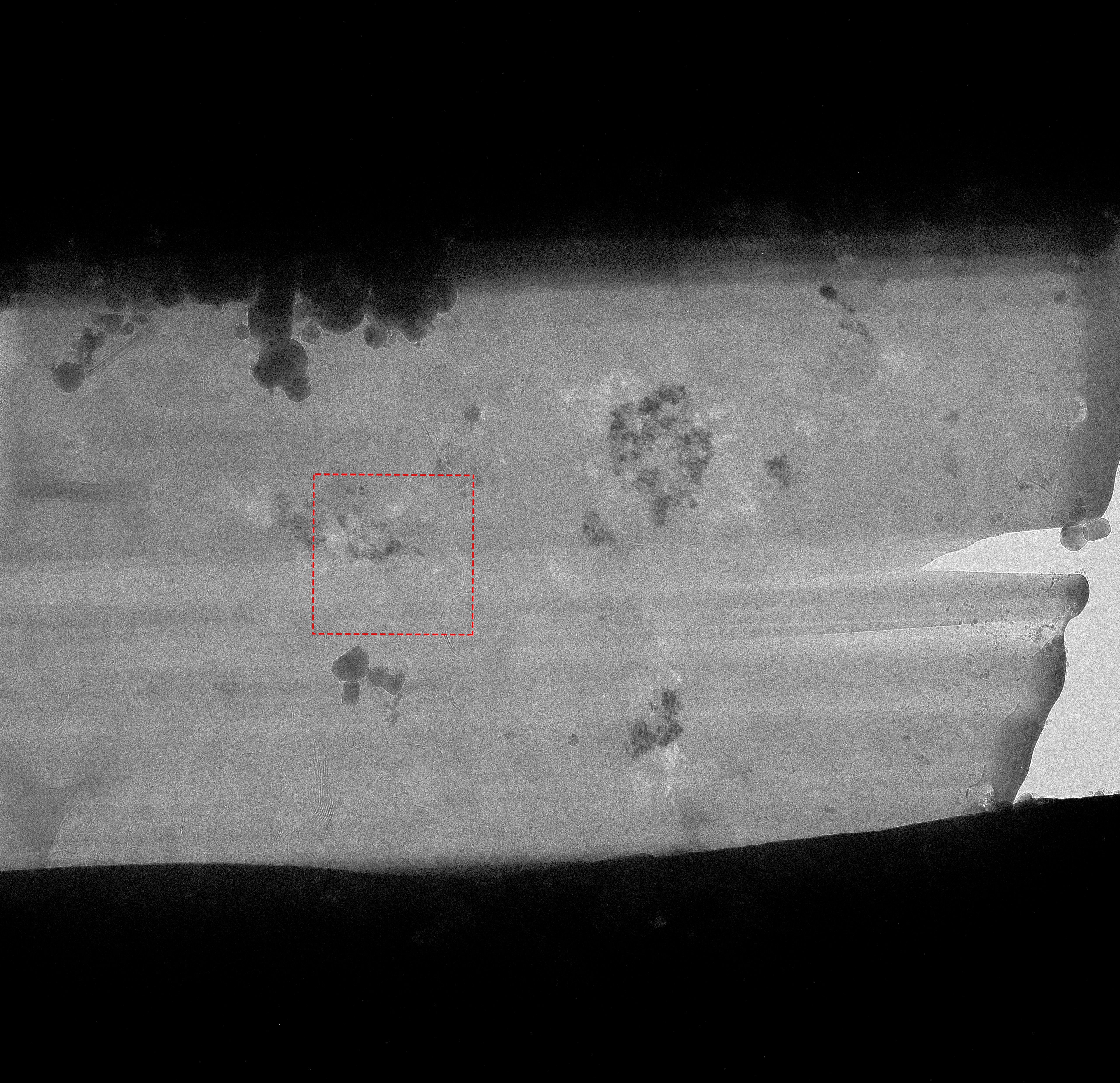

Supplement: Supplementary file 8 — Raw cryo-EM images of all the cryo-lamellae shown in Supplementary Fig. 1. The locations of centrioles are marked by dashed squares. [file 41592_2022_1748_MOESM8_ESM.zip › Supplementary_Data1/Lamella13_Location13.jpg]

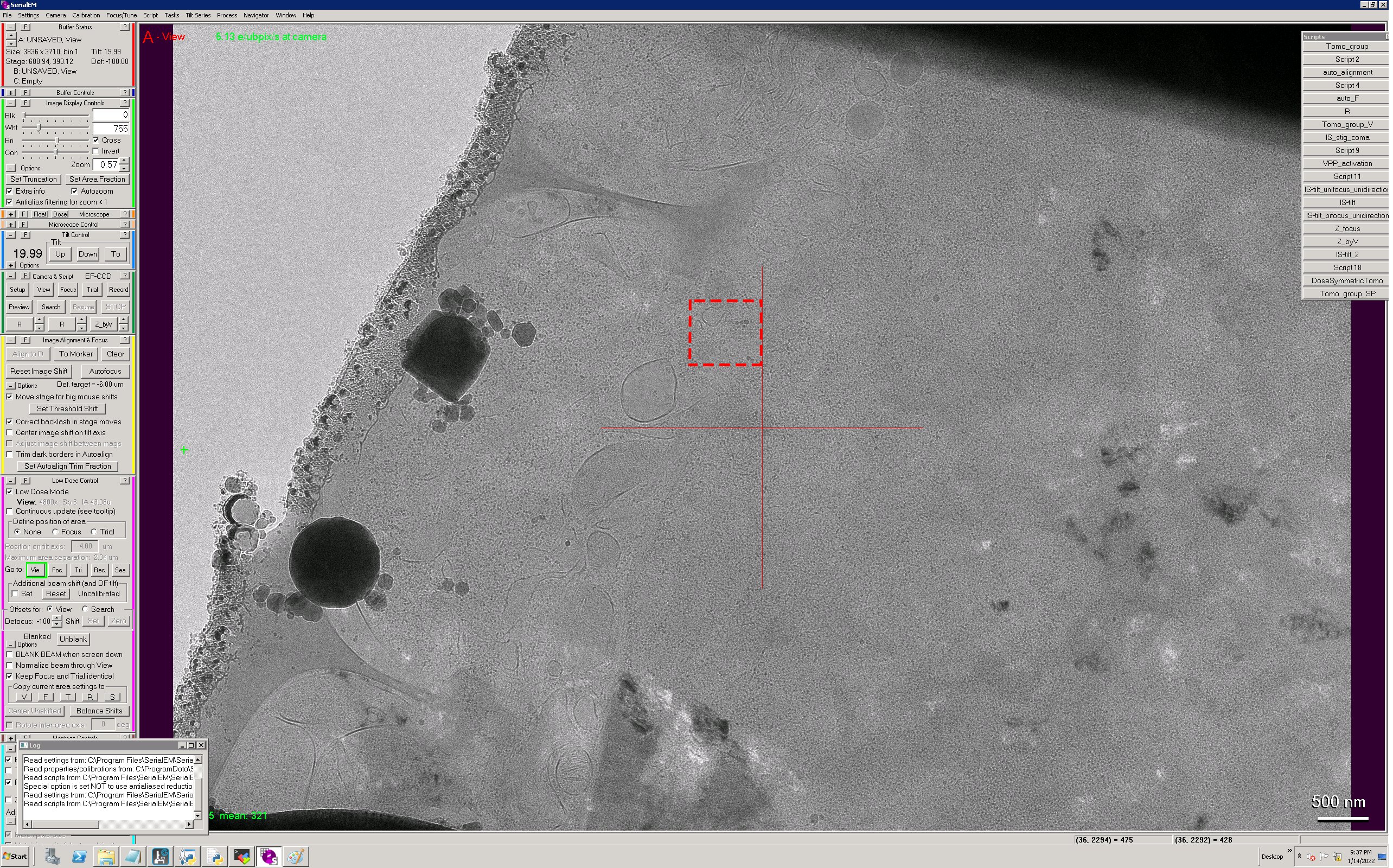

Supplement: Supplementary file 8 — Raw cryo-EM images of all the cryo-lamellae shown in Supplementary Fig. 1. The locations of centrioles are marked by dashed squares. [file 41592_2022_1748_MOESM8_ESM.zip › Supplementary_Data1/Lamella73_Location66.jpg]

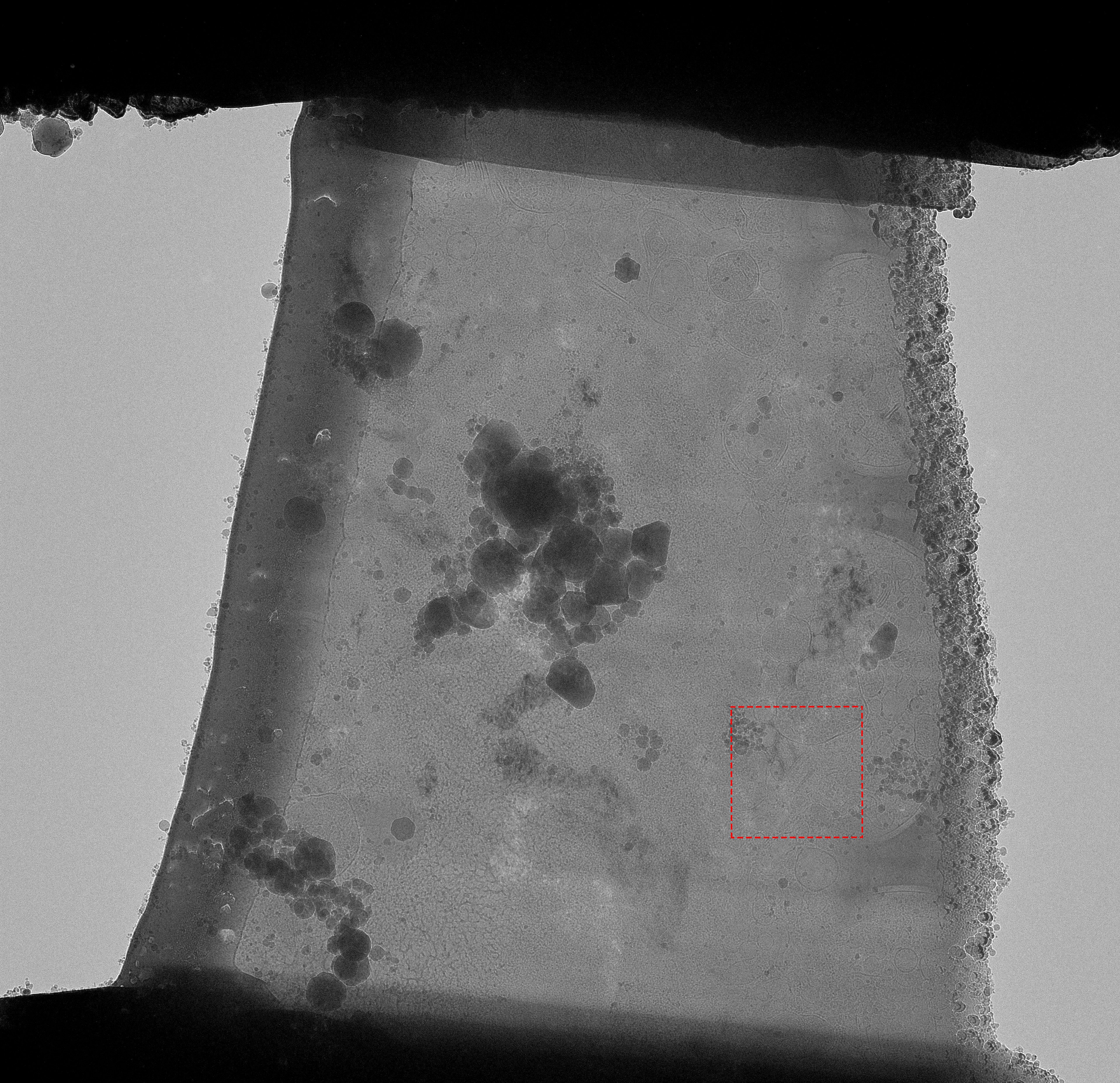

Supplement: Supplementary file 8 — Raw cryo-EM images of all the cryo-lamellae shown in Supplementary Fig. 1. The locations of centrioles are marked by dashed squares. [file 41592_2022_1748_MOESM8_ESM.zip › Supplementary_Data1/Lamella22_Location21.jpg]

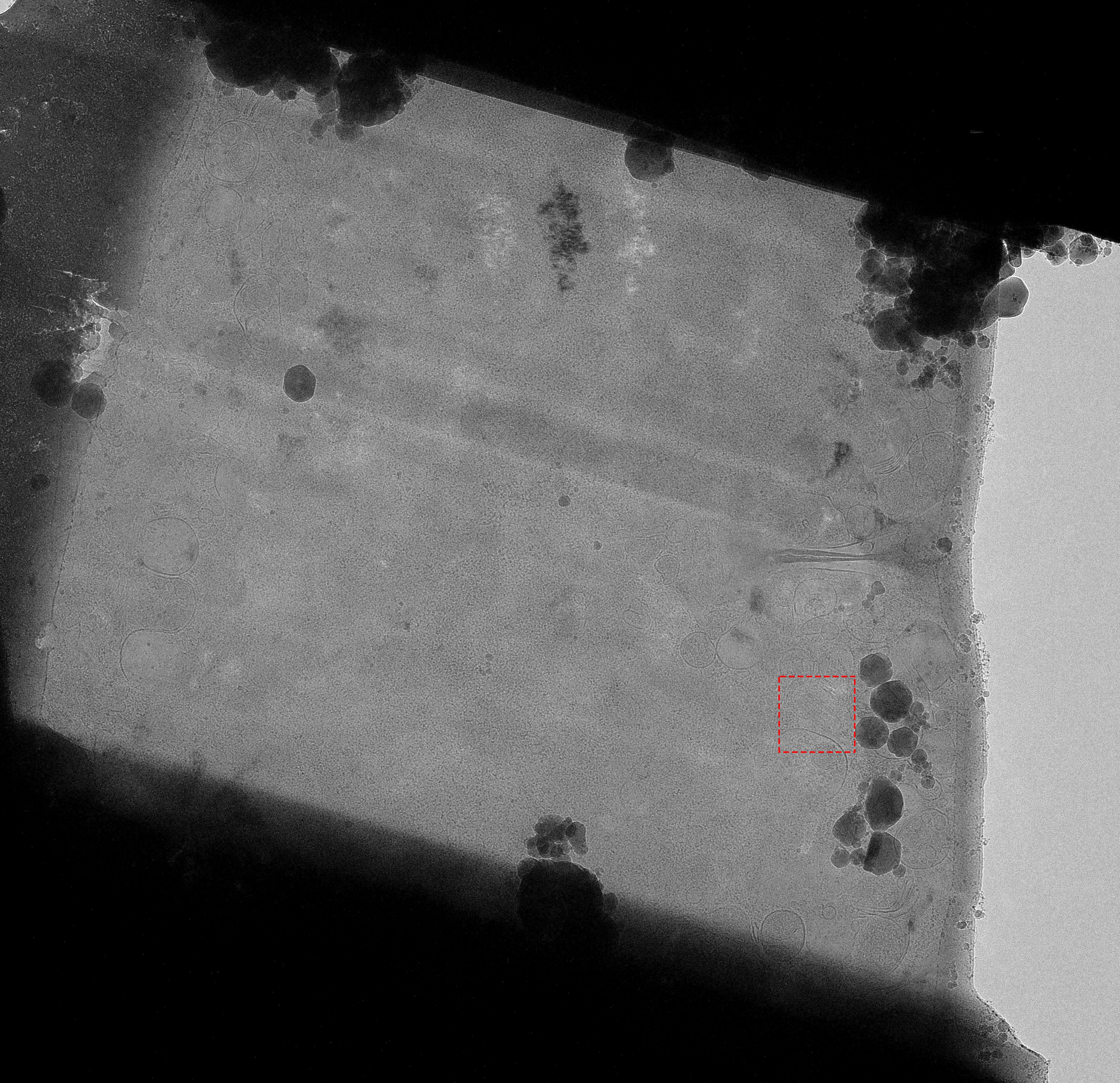

Supplement: Supplementary file 8 — Raw cryo-EM images of all the cryo-lamellae shown in Supplementary Fig. 1. The locations of centrioles are marked by dashed squares. [file 41592_2022_1748_MOESM8_ESM.zip › Supplementary_Data1/Lamella37_Location35.jpg]

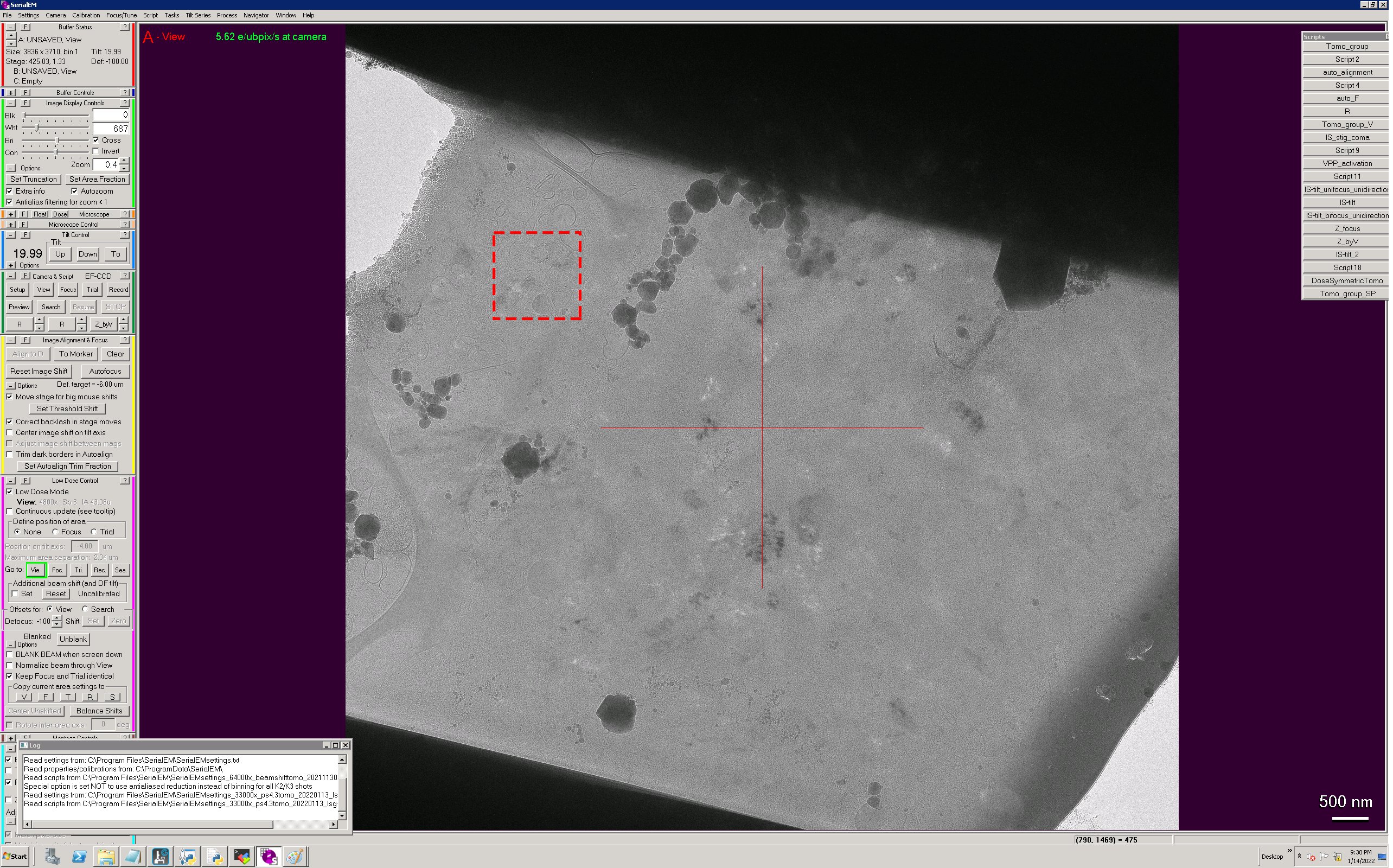

Supplement: Supplementary file 8 — Raw cryo-EM images of all the cryo-lamellae shown in Supplementary Fig. 1. The locations of centrioles are marked by dashed squares. [file 41592_2022_1748_MOESM8_ESM.zip › Supplementary_Data1/Lamella70_Location63.jpg]

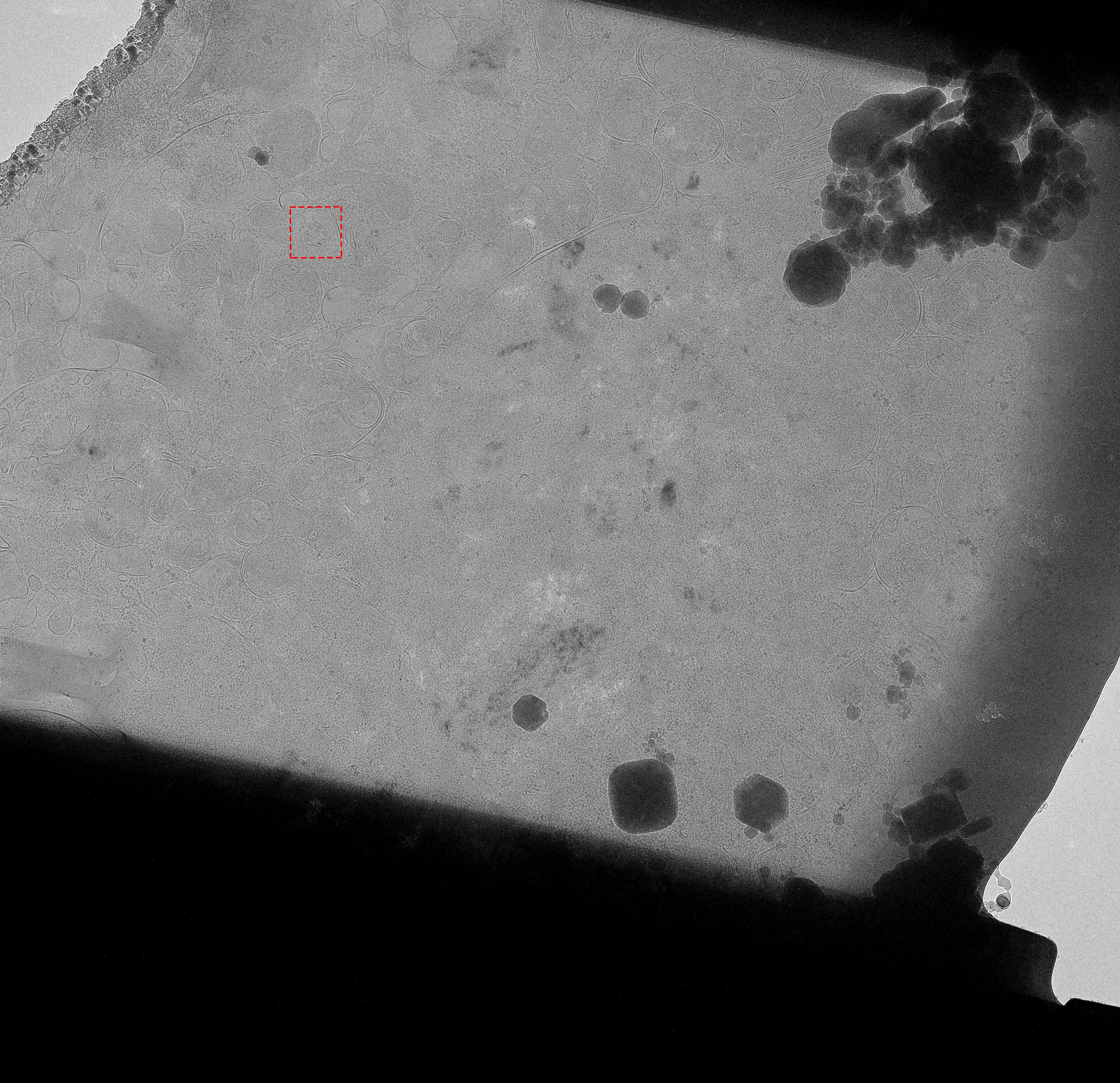

Supplement: Supplementary file 8 — Raw cryo-EM images of all the cryo-lamellae shown in Supplementary Fig. 1. The locations of centrioles are marked by dashed squares. [file 41592_2022_1748_MOESM8_ESM.zip › Supplementary_Data1/Lamella57_Location54.jpg]

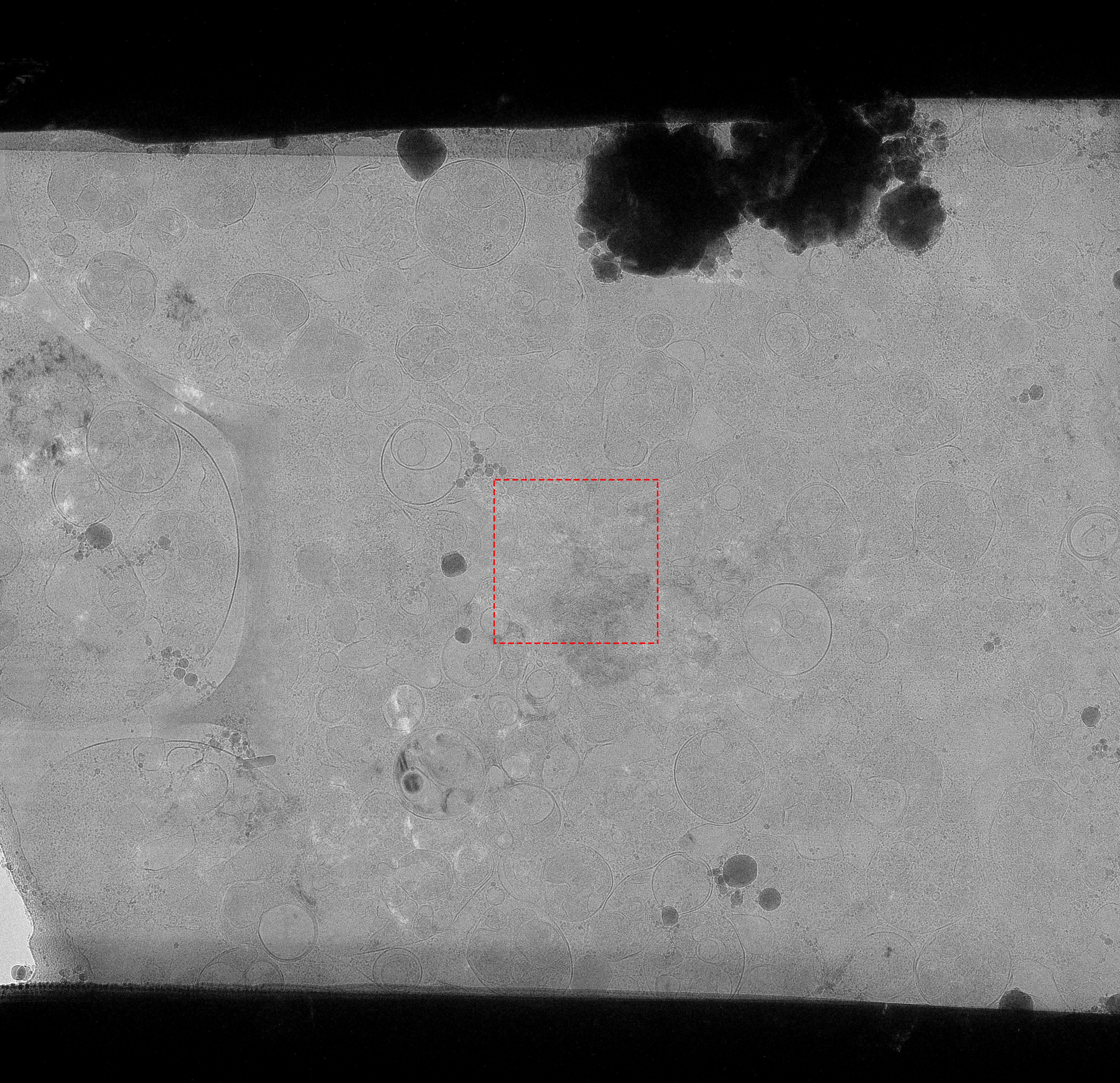

Supplement: Supplementary file 8 — Raw cryo-EM images of all the cryo-lamellae shown in Supplementary Fig. 1. The locations of centrioles are marked by dashed squares. [file 41592_2022_1748_MOESM8_ESM.zip › Supplementary_Data1/Lamella18_Location17.jpg]

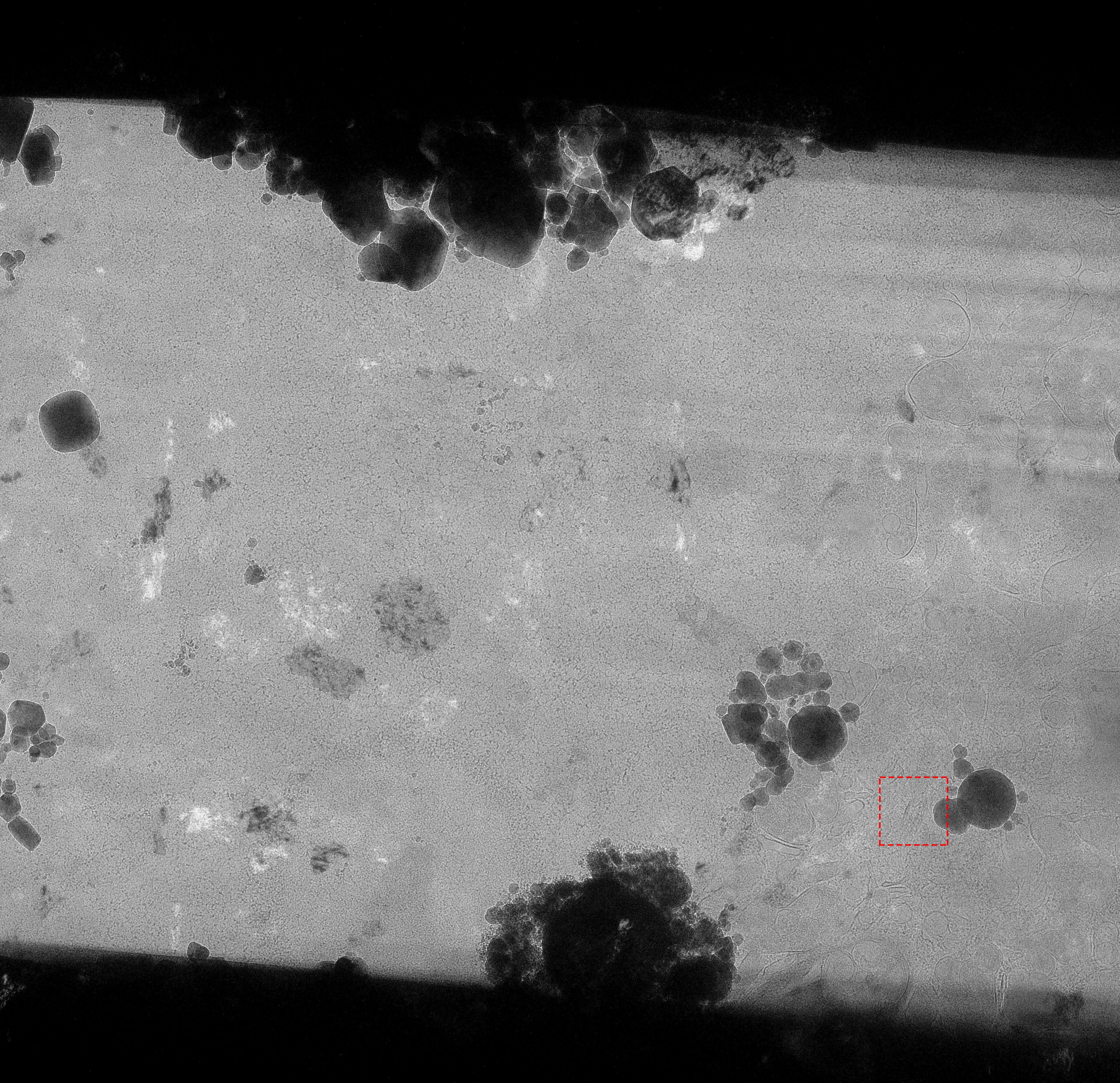

Supplement: Supplementary file 8 — Raw cryo-EM images of all the cryo-lamellae shown in Supplementary Fig. 1. The locations of centrioles are marked by dashed squares. [file 41592_2022_1748_MOESM8_ESM.zip › Supplementary_Data1/Lamella45_Location42.jpg]

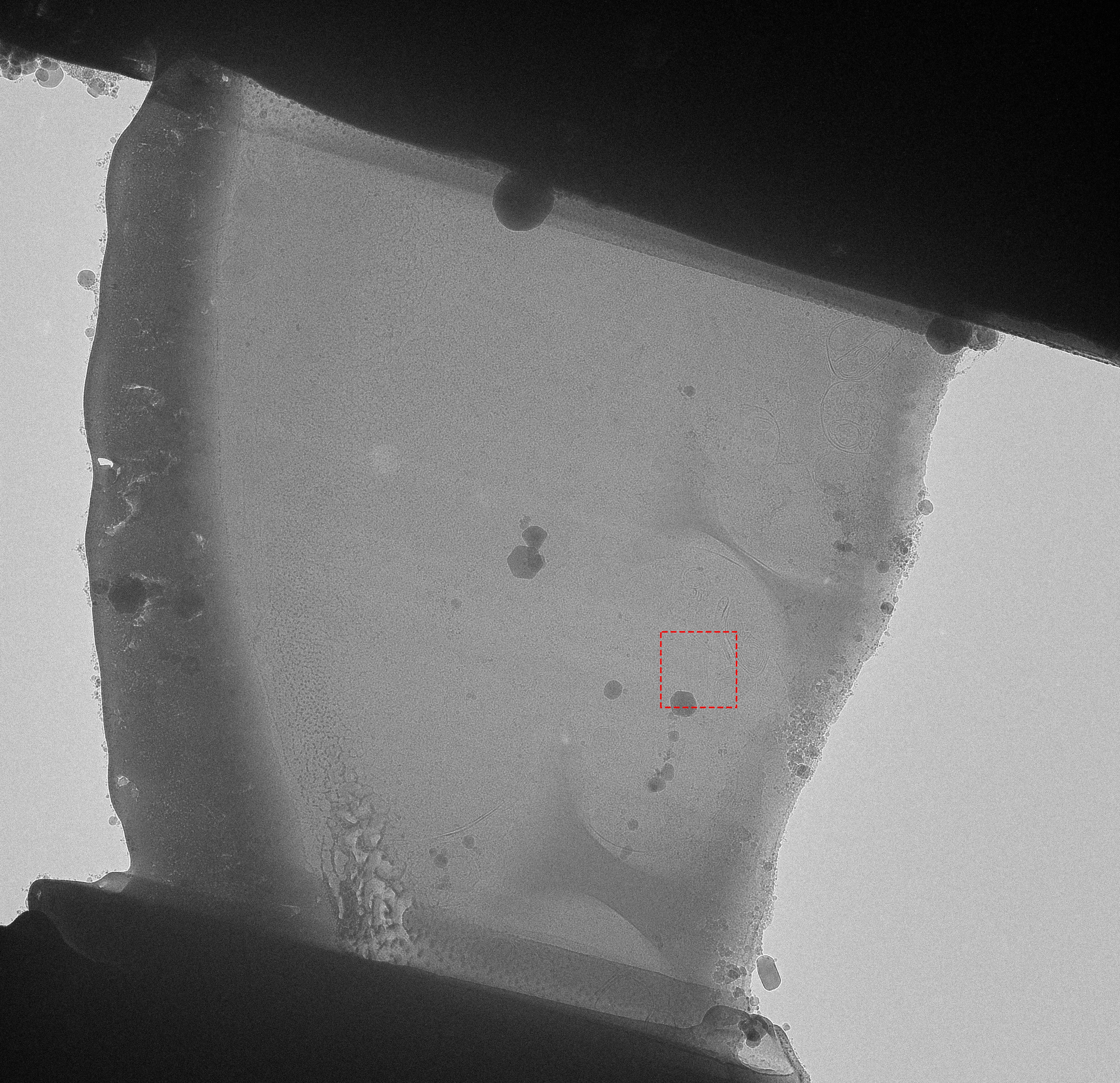

Supplement: Supplementary file 8 — Raw cryo-EM images of all the cryo-lamellae shown in Supplementary Fig. 1. The locations of centrioles are marked by dashed squares. [file 41592_2022_1748_MOESM8_ESM.zip › Supplementary_Data1/Lamella38_Location36.jpg]

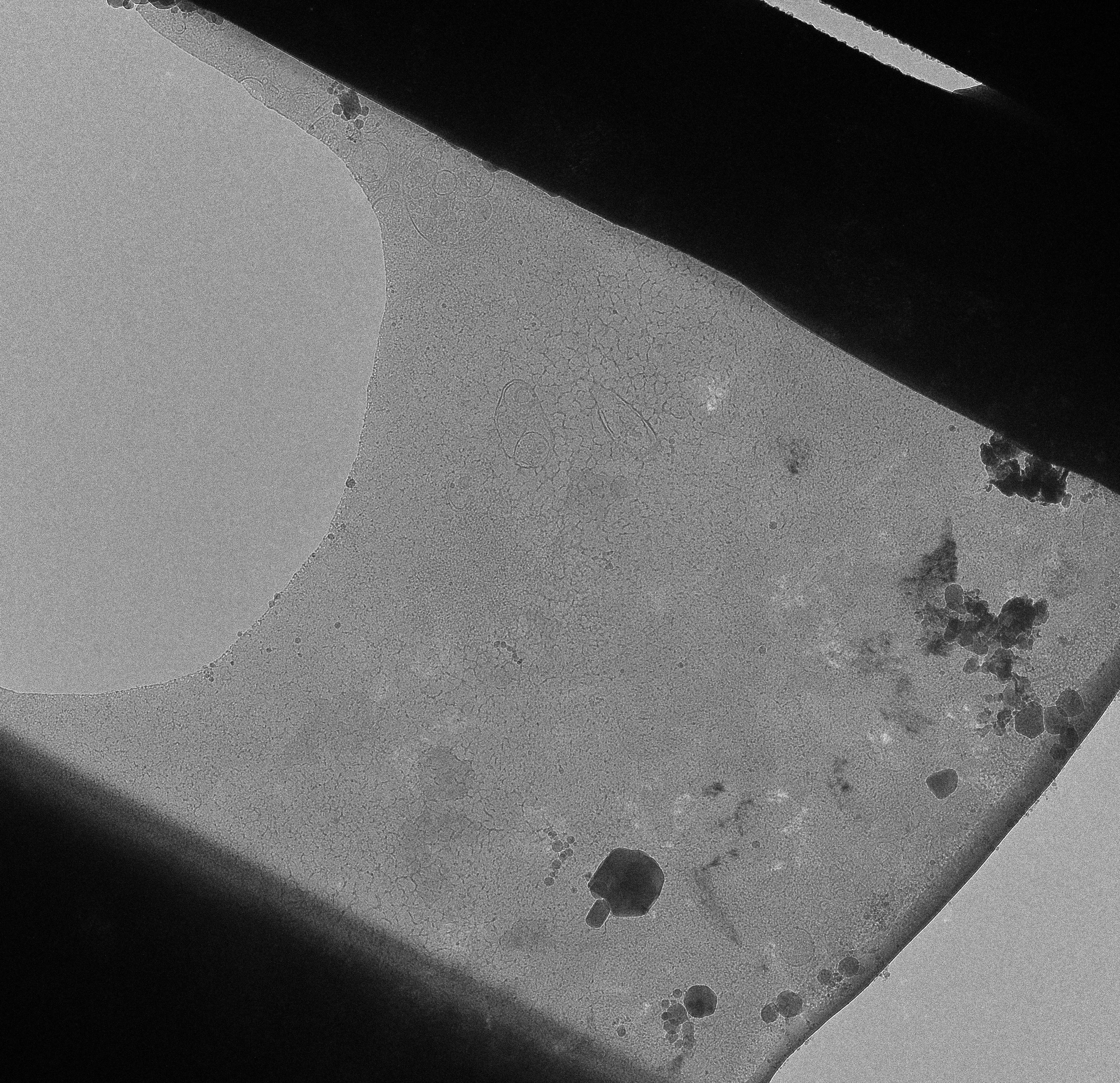

Supplement: Supplementary file 8 — Raw cryo-EM images of all the cryo-lamellae shown in Supplementary Fig. 1. The locations of centrioles are marked by dashed squares. [file 41592_2022_1748_MOESM8_ESM.zip › Supplementary_Data1/Lamella62_NoLocation.jpg]

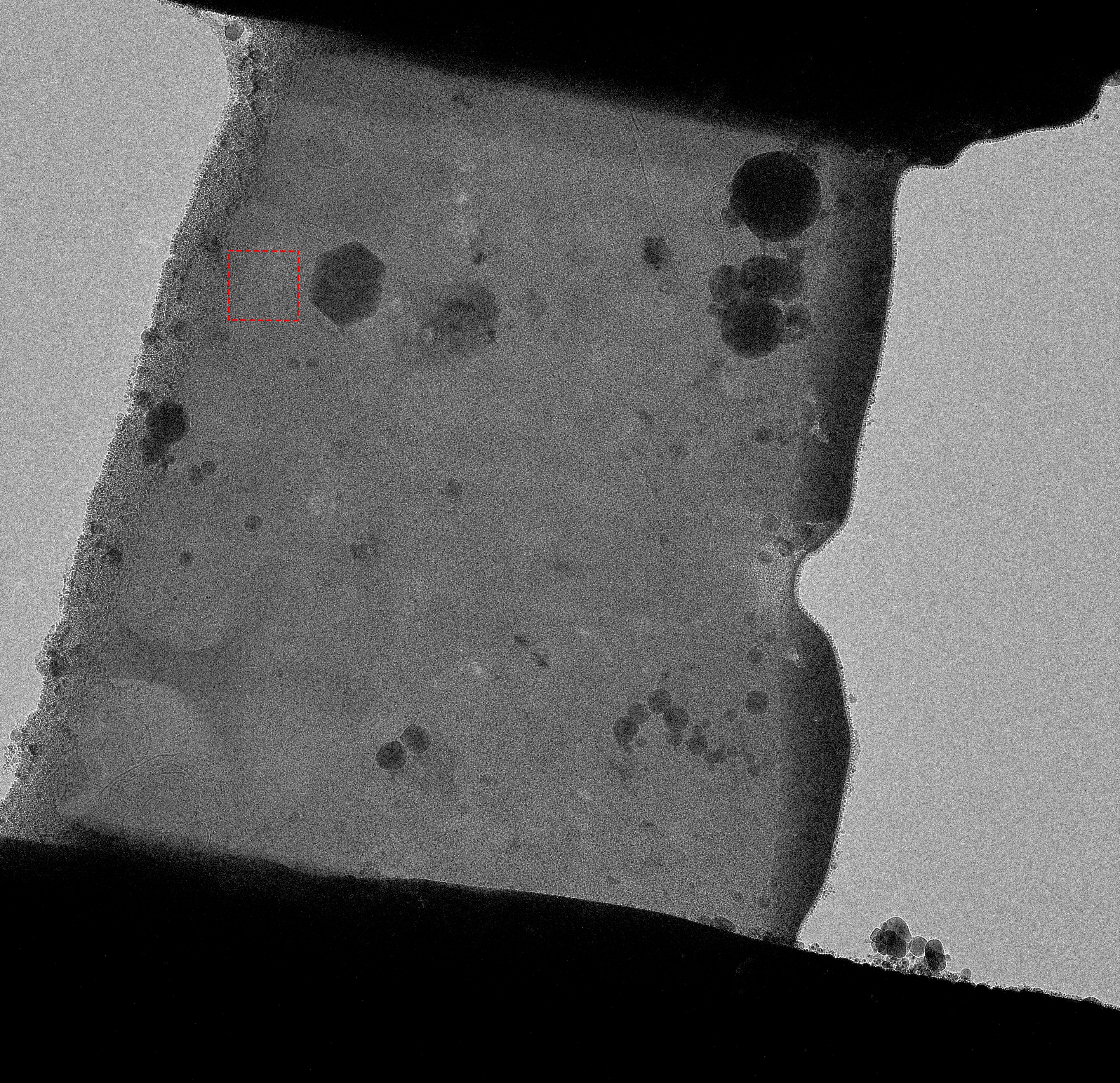

Supplement: Supplementary file 8 — Raw cryo-EM images of all the cryo-lamellae shown in Supplementary Fig. 1. The locations of centrioles are marked by dashed squares. [file 41592_2022_1748_MOESM8_ESM.zip › Supplementary_Data1/Lamella56_Location53.jpg]

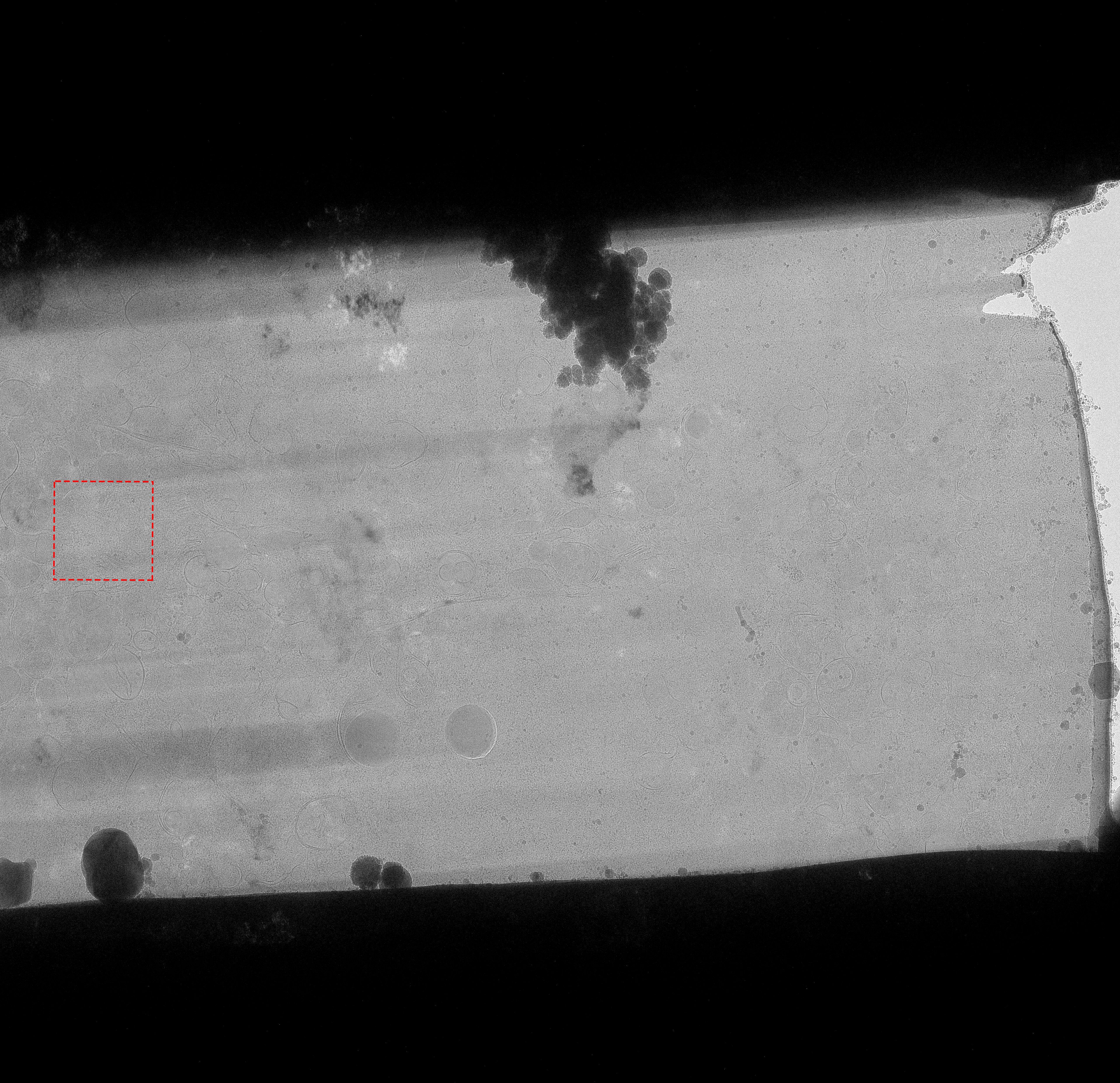

Supplement: Supplementary file 8 — Raw cryo-EM images of all the cryo-lamellae shown in Supplementary Fig. 1. The locations of centrioles are marked by dashed squares. [file 41592_2022_1748_MOESM8_ESM.zip › Supplementary_Data1/Lamella11_Location11.jpg]

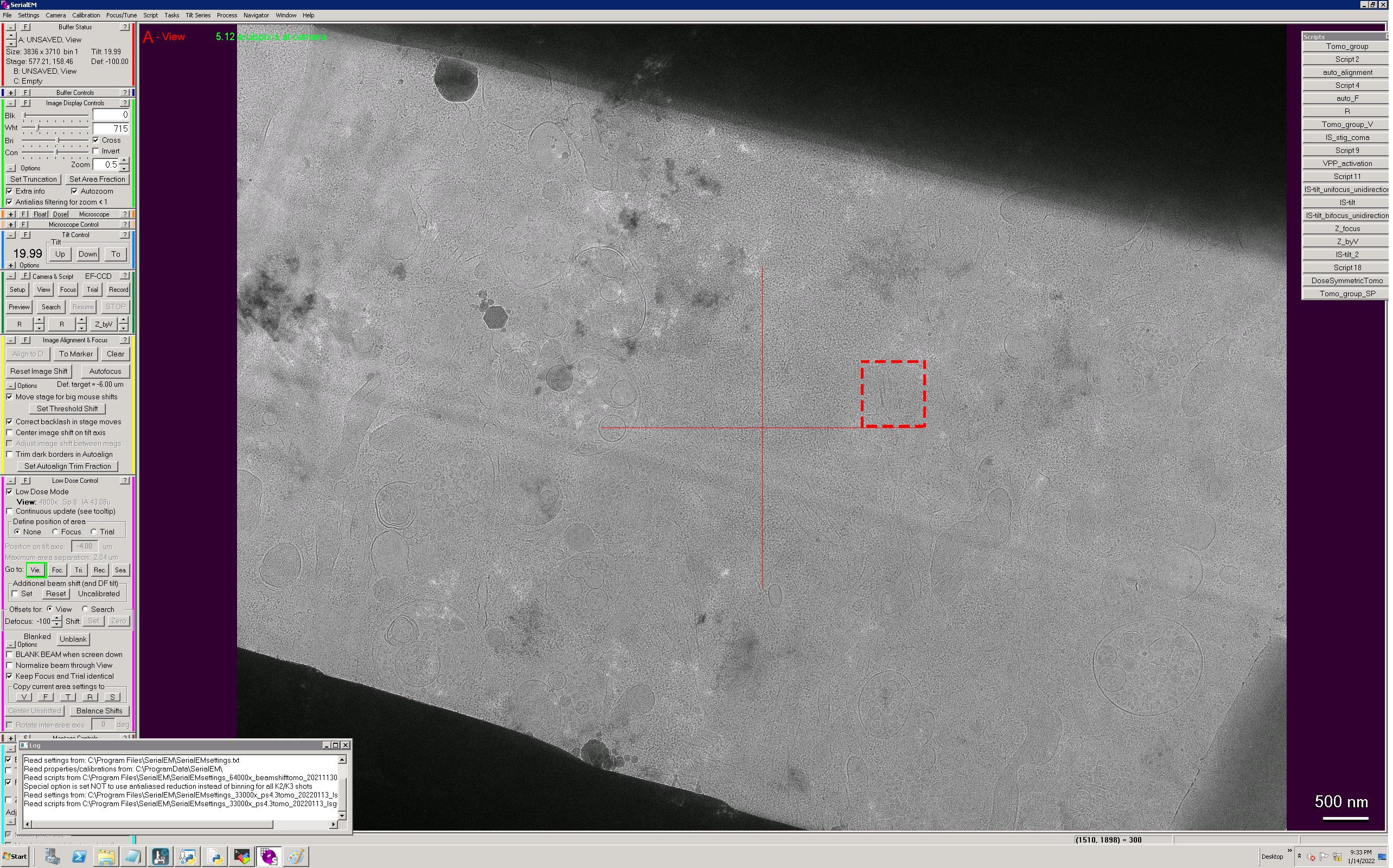

Supplement: Supplementary file 8 — Raw cryo-EM images of all the cryo-lamellae shown in Supplementary Fig. 1. The locations of centrioles are marked by dashed squares. [file 41592_2022_1748_MOESM8_ESM.zip › Supplementary_Data1/Lamella71_Location64.jpg]

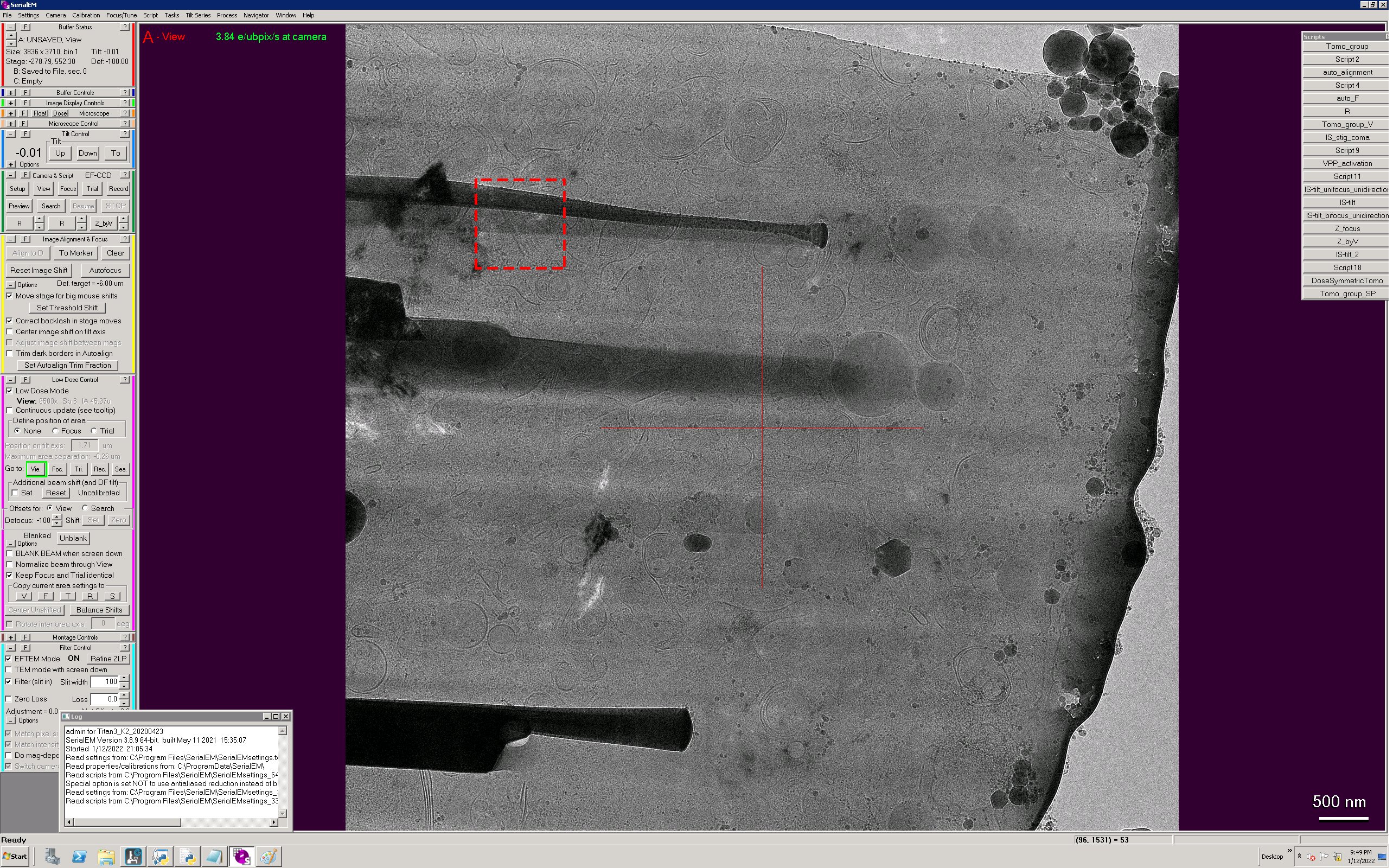

Supplement: Supplementary file 8 — Raw cryo-EM images of all the cryo-lamellae shown in Supplementary Fig. 1. The locations of centrioles are marked by dashed squares. [file 41592_2022_1748_MOESM8_ESM.zip › Supplementary_Data1/Lamella64_Location58.jpg]

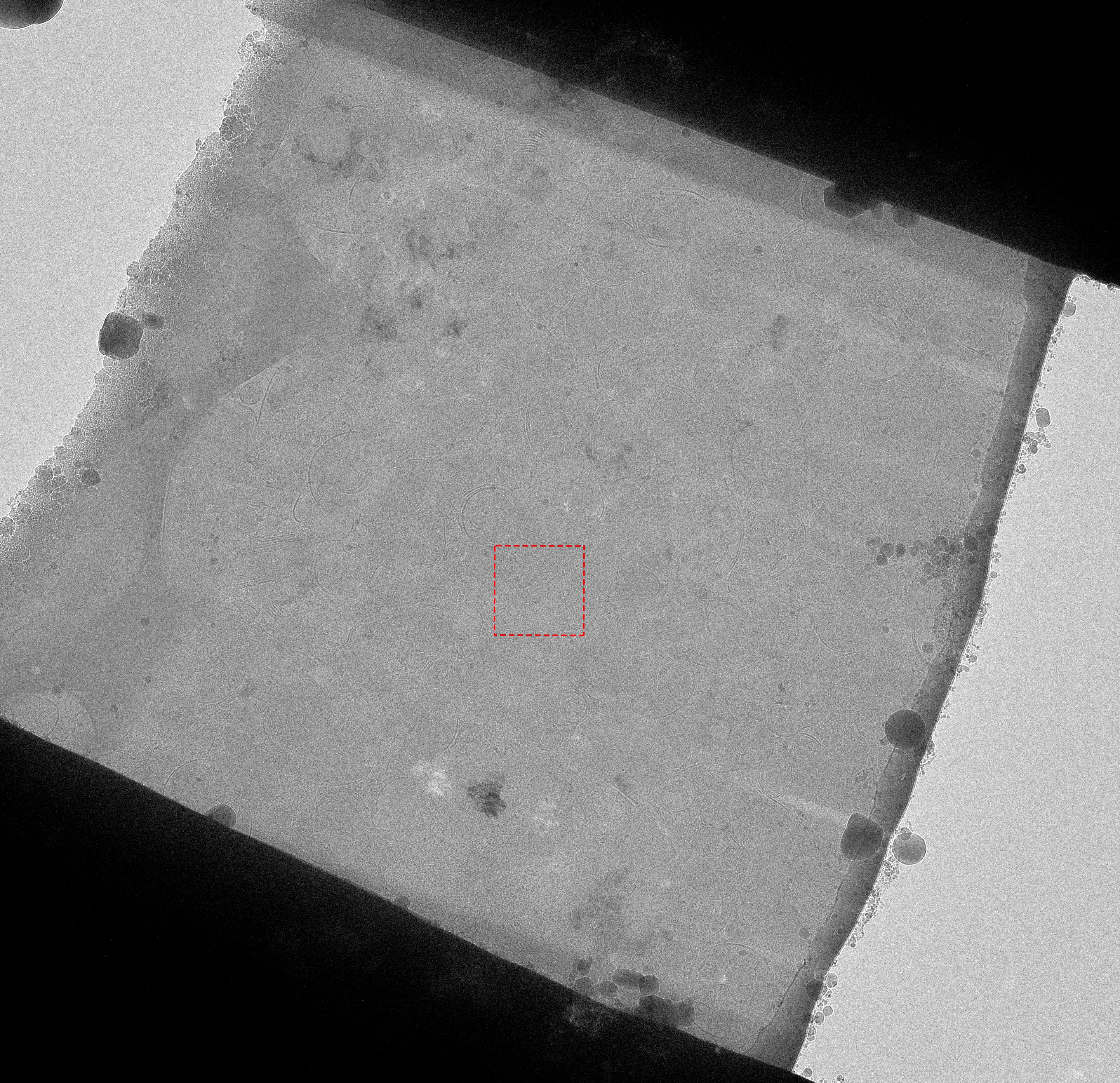

Supplement: Supplementary file 8 — Raw cryo-EM images of all the cryo-lamellae shown in Supplementary Fig. 1. The locations of centrioles are marked by dashed squares. [file 41592_2022_1748_MOESM8_ESM.zip › Supplementary_Data1/Lamella02_Location02.jpg]

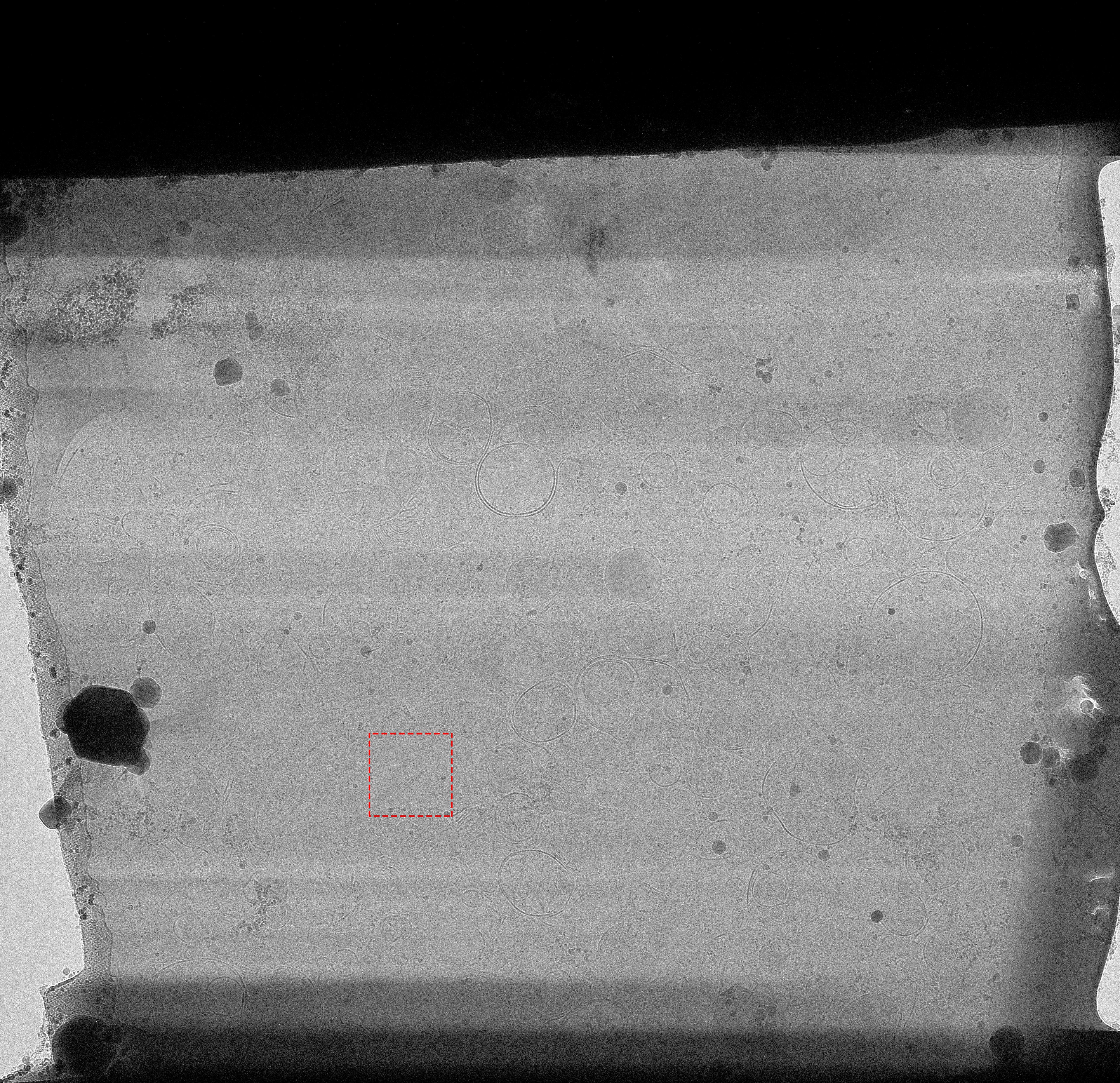

Supplement: Supplementary file 8 — Raw cryo-EM images of all the cryo-lamellae shown in Supplementary Fig. 1. The locations of centrioles are marked by dashed squares. [file 41592_2022_1748_MOESM8_ESM.zip › Supplementary_Data1/Lamella17_Location16.jpg]

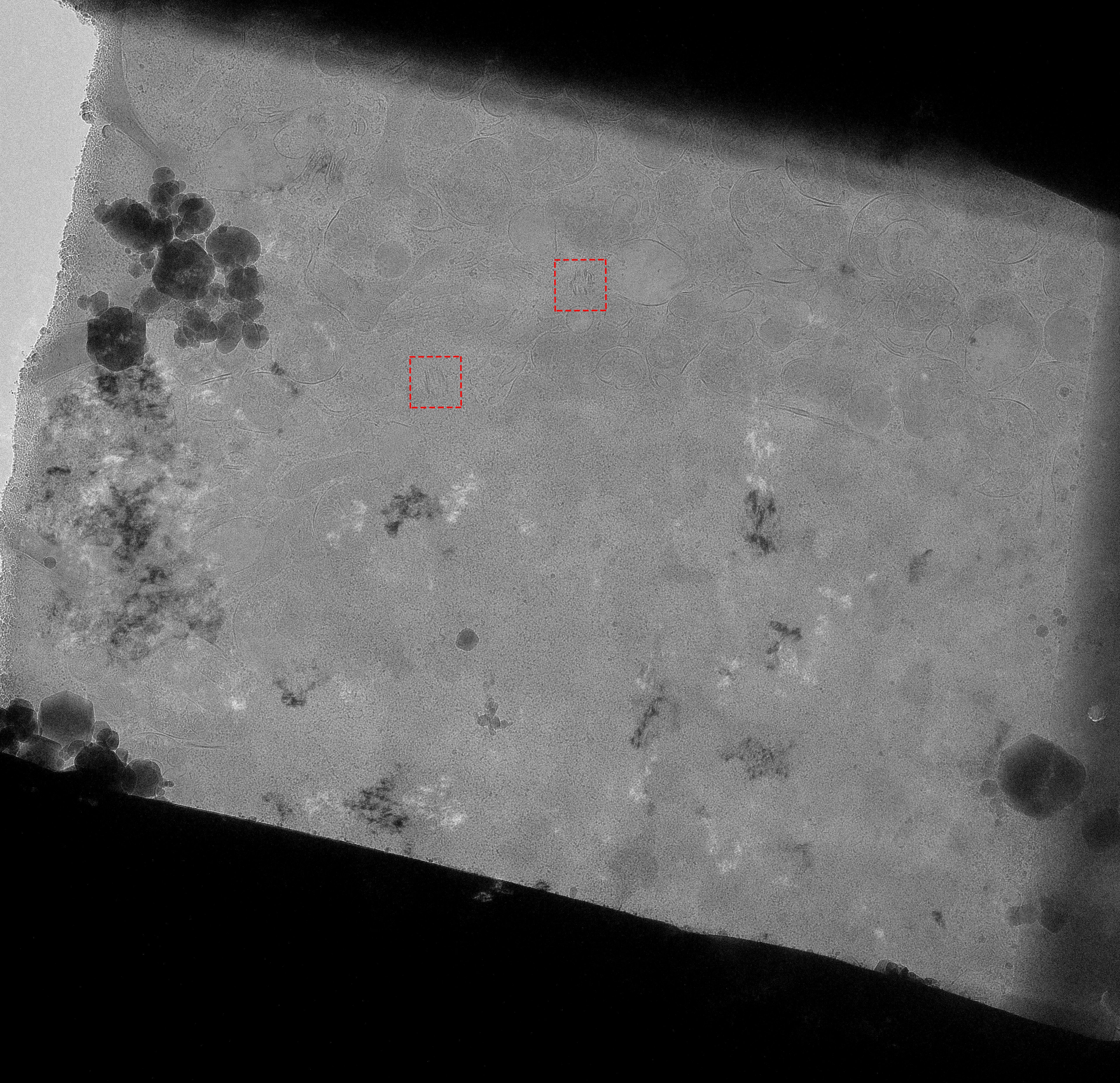

Supplement: Supplementary file 8 — Raw cryo-EM images of all the cryo-lamellae shown in Supplementary Fig. 1. The locations of centrioles are marked by dashed squares. [file 41592_2022_1748_MOESM8_ESM.zip › Supplementary_Data1/Lamella58_Location55.jpg]

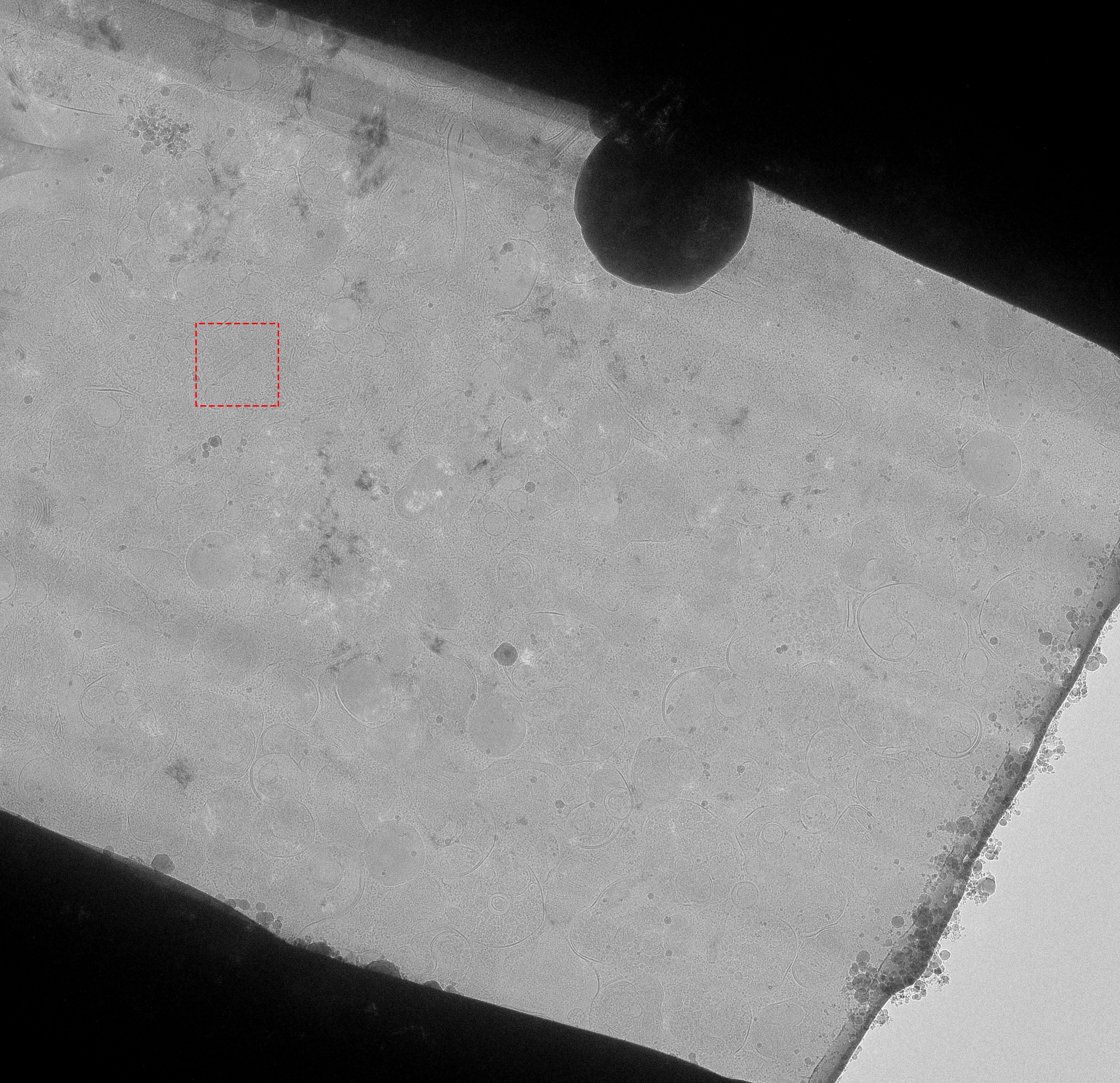

Supplement: Supplementary file 8 — Raw cryo-EM images of all the cryo-lamellae shown in Supplementary Fig. 1. The locations of centrioles are marked by dashed squares. [file 41592_2022_1748_MOESM8_ESM.zip › Supplementary_Data1/Lamella04_Location04.jpg]

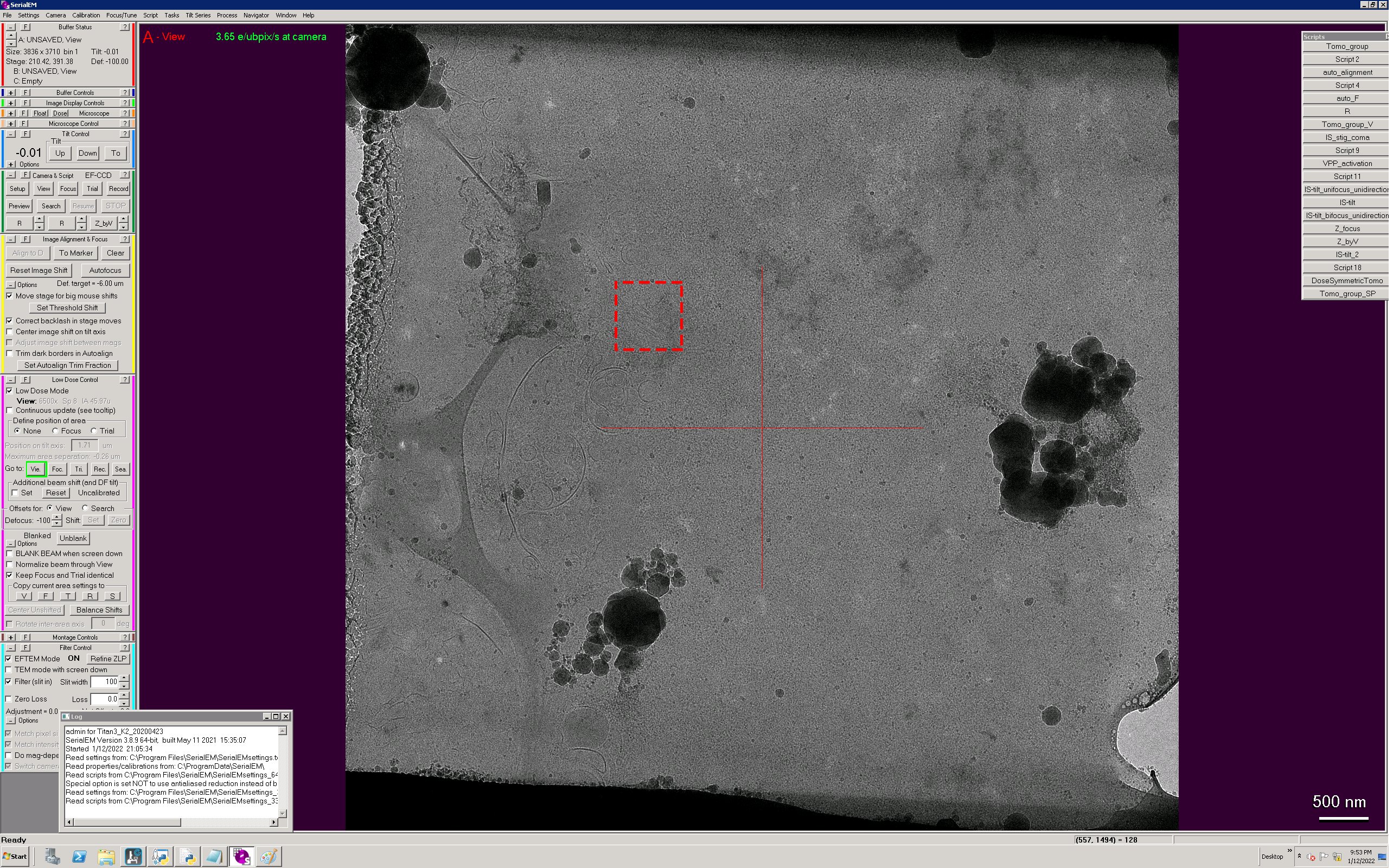

Supplement: Supplementary file 8 — Raw cryo-EM images of all the cryo-lamellae shown in Supplementary Fig. 1. The locations of centrioles are marked by dashed squares. [file 41592_2022_1748_MOESM8_ESM.zip › Supplementary_Data1/Lamella65_Location59.jpg]

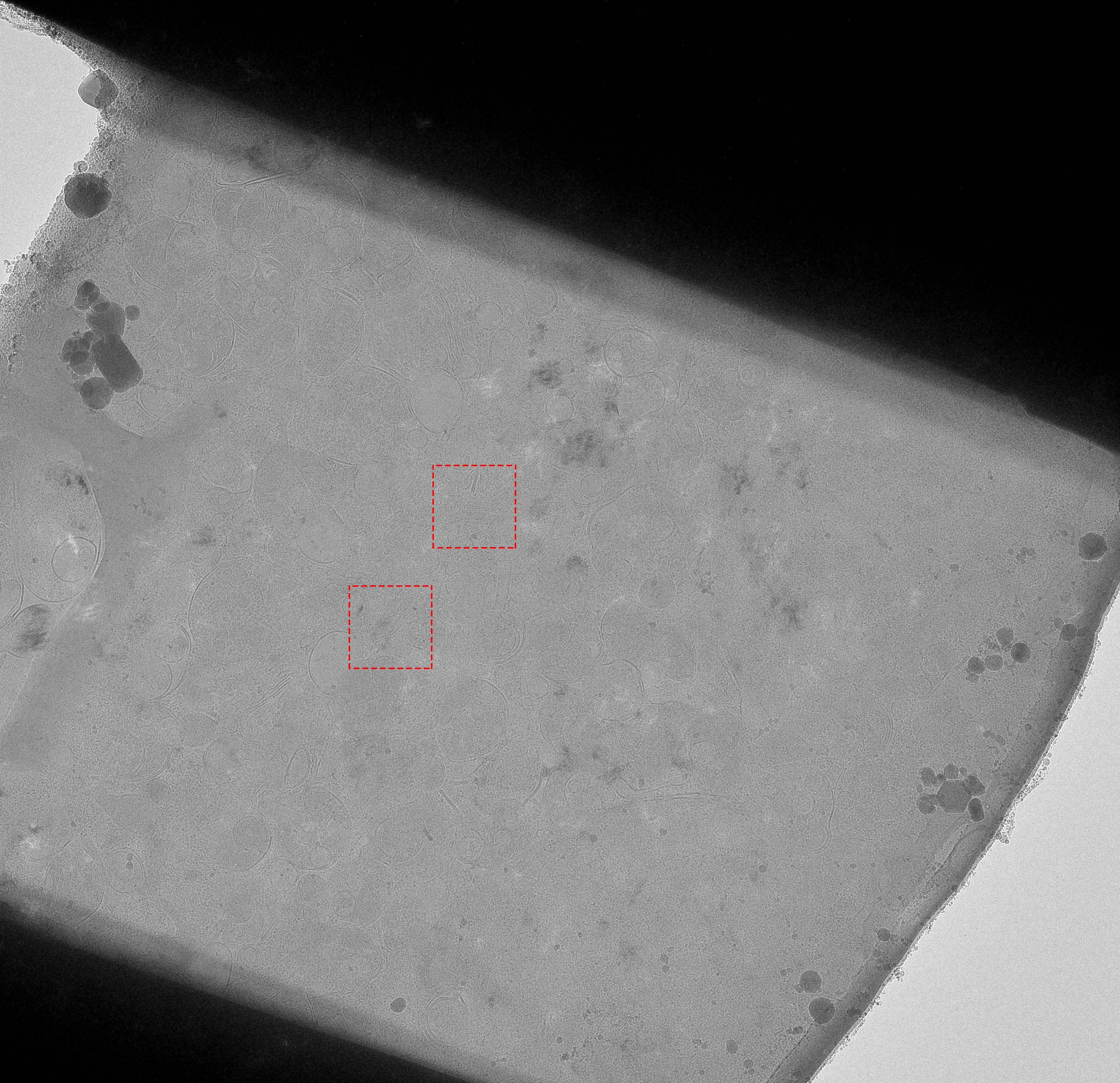

Supplement: Supplementary file 8 — Raw cryo-EM images of all the cryo-lamellae shown in Supplementary Fig. 1. The locations of centrioles are marked by dashed squares. [file 41592_2022_1748_MOESM8_ESM.zip › Supplementary_Data1/Lamella03_Location03.jpg]

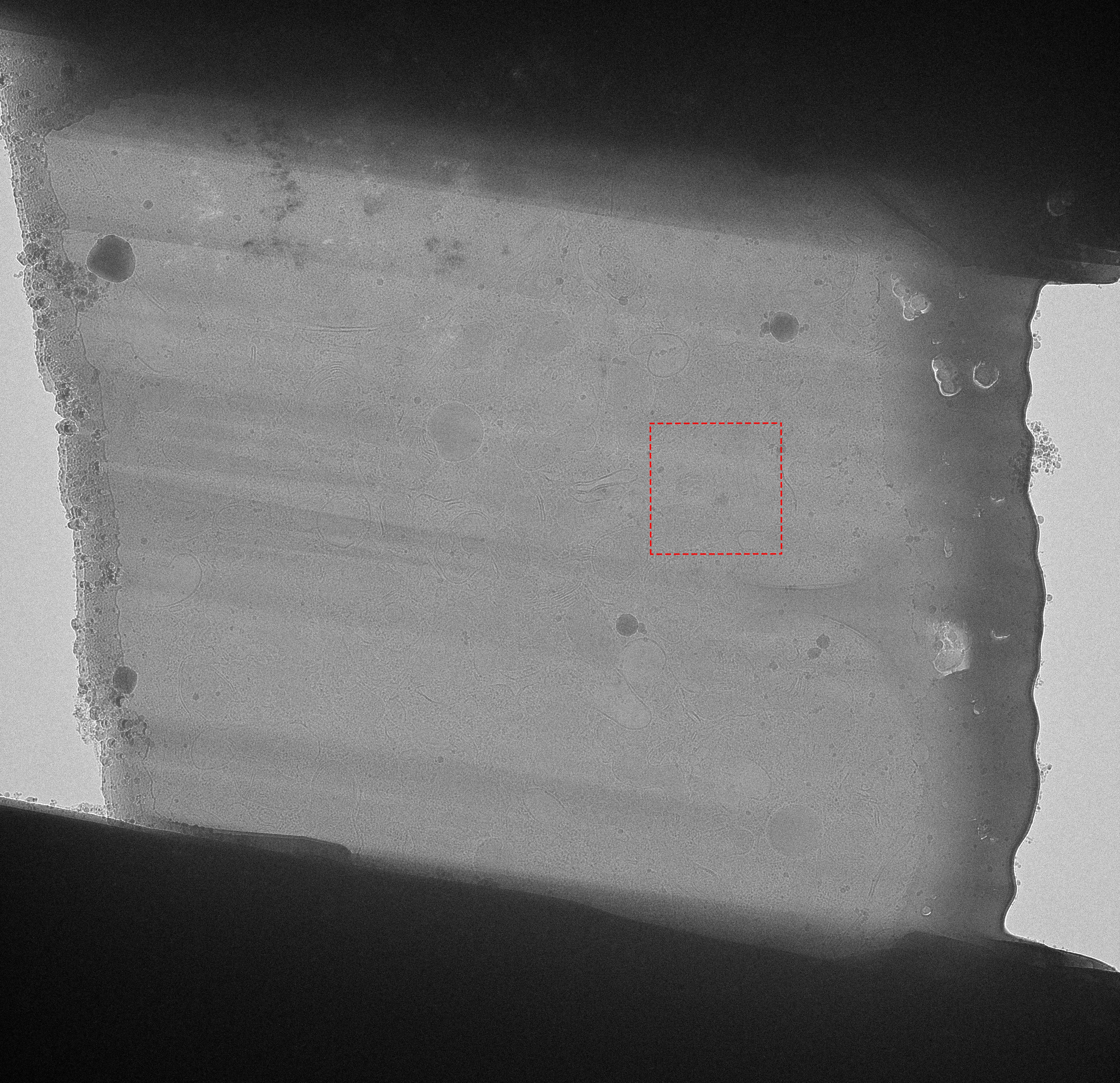

Supplement: Supplementary file 8 — Raw cryo-EM images of all the cryo-lamellae shown in Supplementary Fig. 1. The locations of centrioles are marked by dashed squares. [file 41592_2022_1748_MOESM8_ESM.zip › Supplementary_Data1/Lamella25_Location24.jpg]

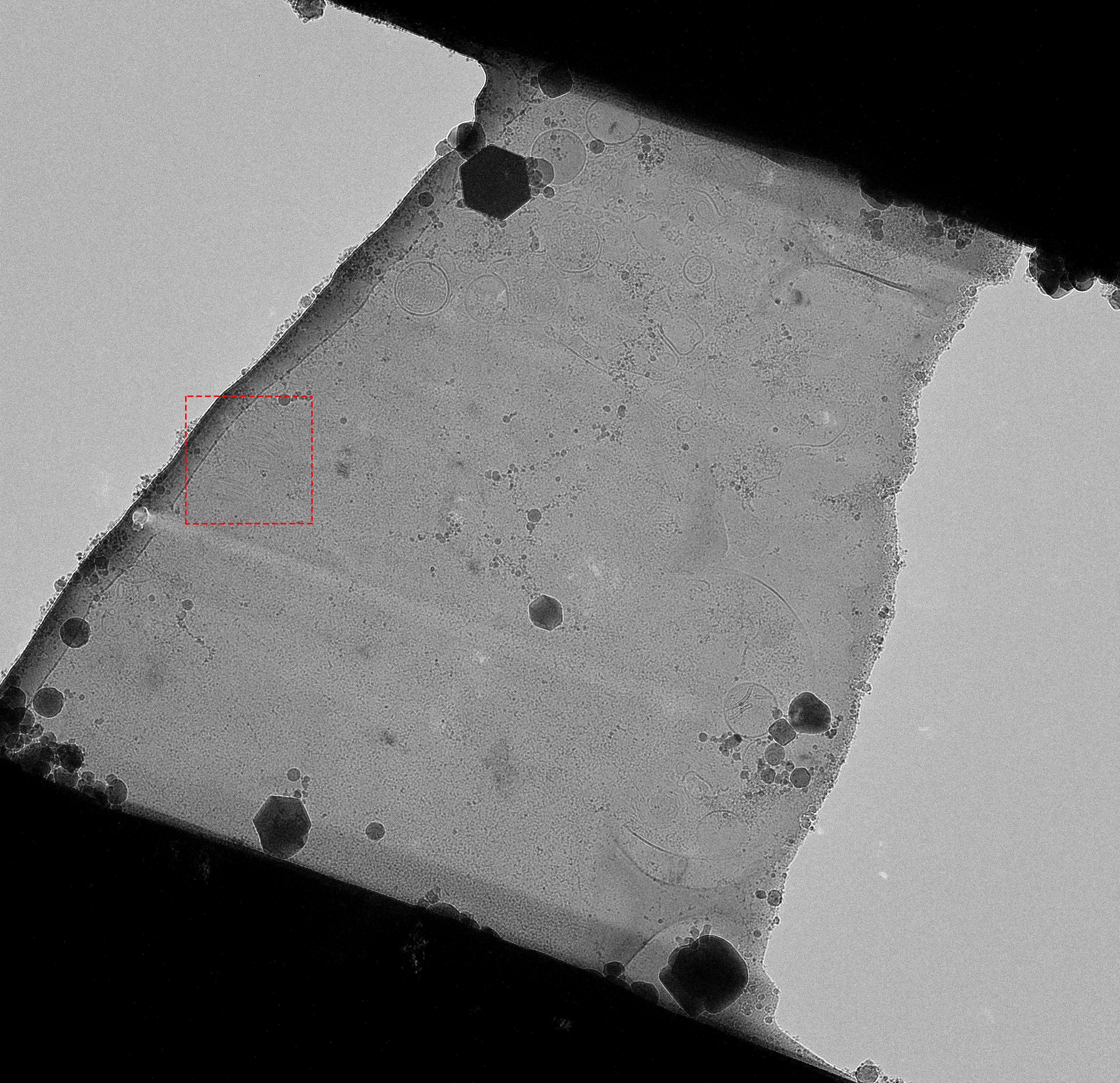

Supplement: Supplementary file 8 — Raw cryo-EM images of all the cryo-lamellae shown in Supplementary Fig. 1. The locations of centrioles are marked by dashed squares. [file 41592_2022_1748_MOESM8_ESM.zip › Supplementary_Data1/Lamella44_Location41.jpg]

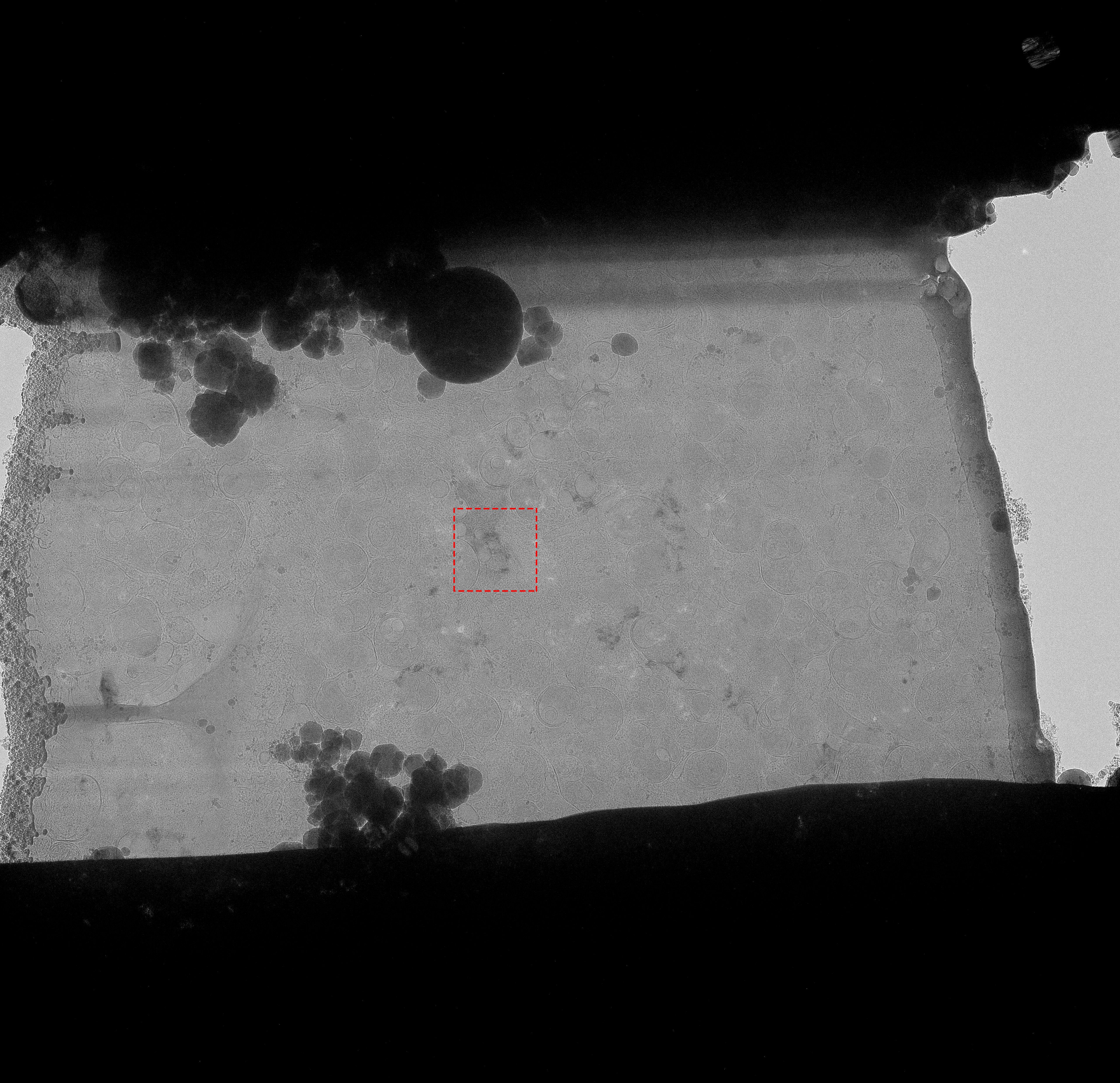

Supplement: Supplementary file 8 — Raw cryo-EM images of all the cryo-lamellae shown in Supplementary Fig. 1. The locations of centrioles are marked by dashed squares. [file 41592_2022_1748_MOESM8_ESM.zip › Supplementary_Data1/Lamella10_Location10.jpg]

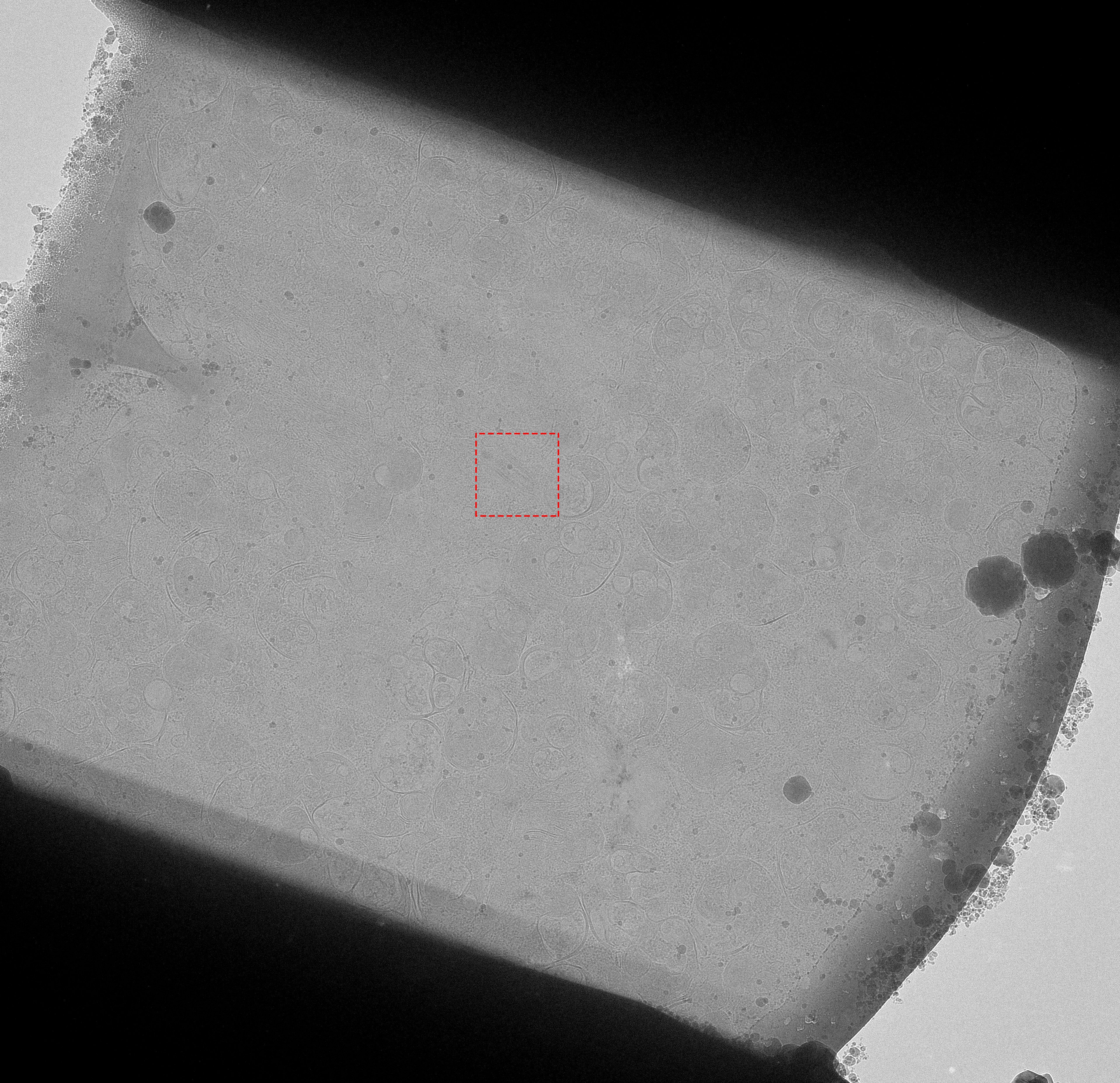

Supplement: Supplementary file 8 — Raw cryo-EM images of all the cryo-lamellae shown in Supplementary Fig. 1. The locations of centrioles are marked by dashed squares. [file 41592_2022_1748_MOESM8_ESM.zip › Supplementary_Data1/Lamella05_Location05.jpg]

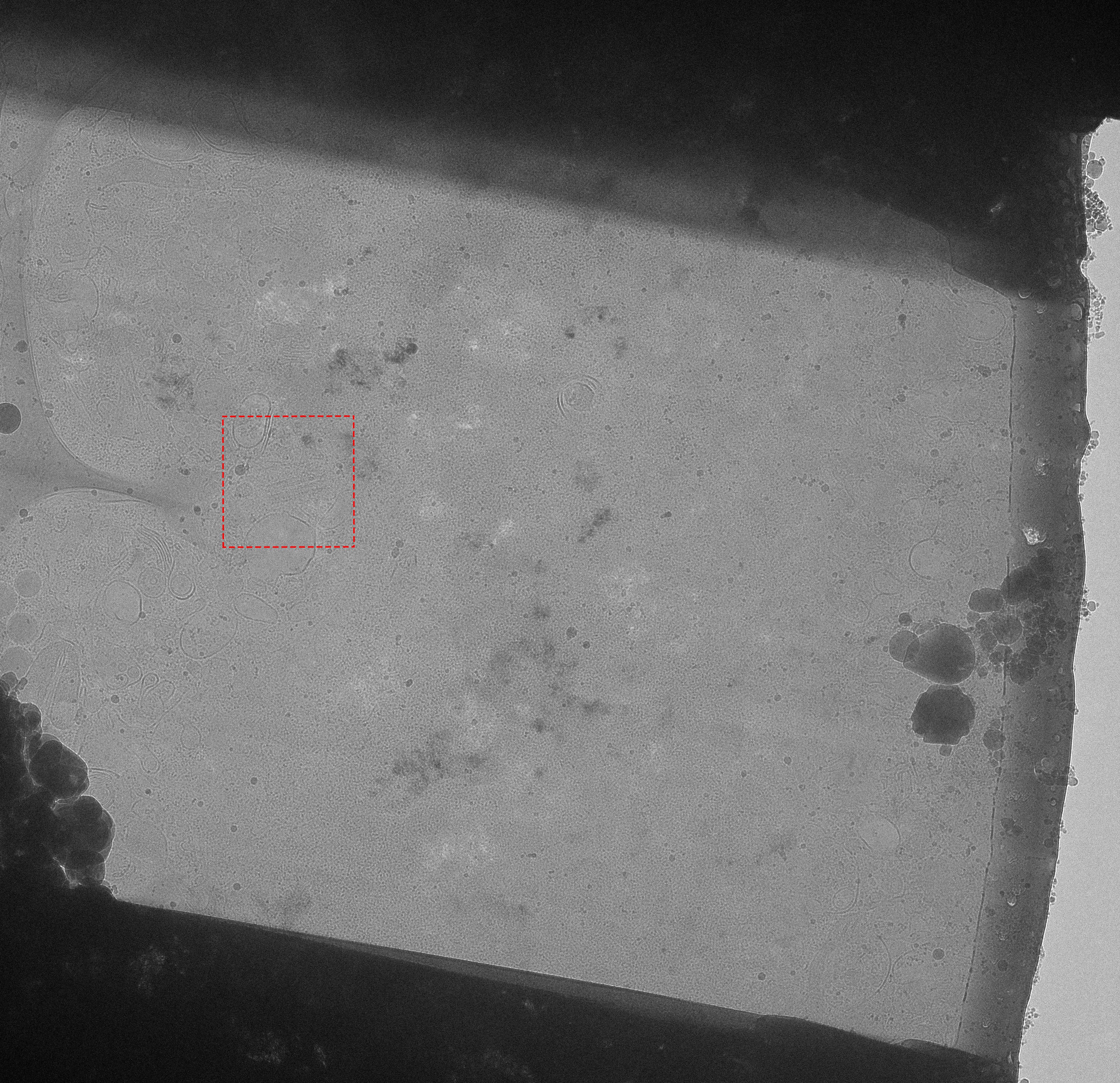

Supplement: Supplementary file 8 — Raw cryo-EM images of all the cryo-lamellae shown in Supplementary Fig. 1. The locations of centrioles are marked by dashed squares. [file 41592_2022_1748_MOESM8_ESM.zip › Supplementary_Data1/Lamella23_Location22.jpg]

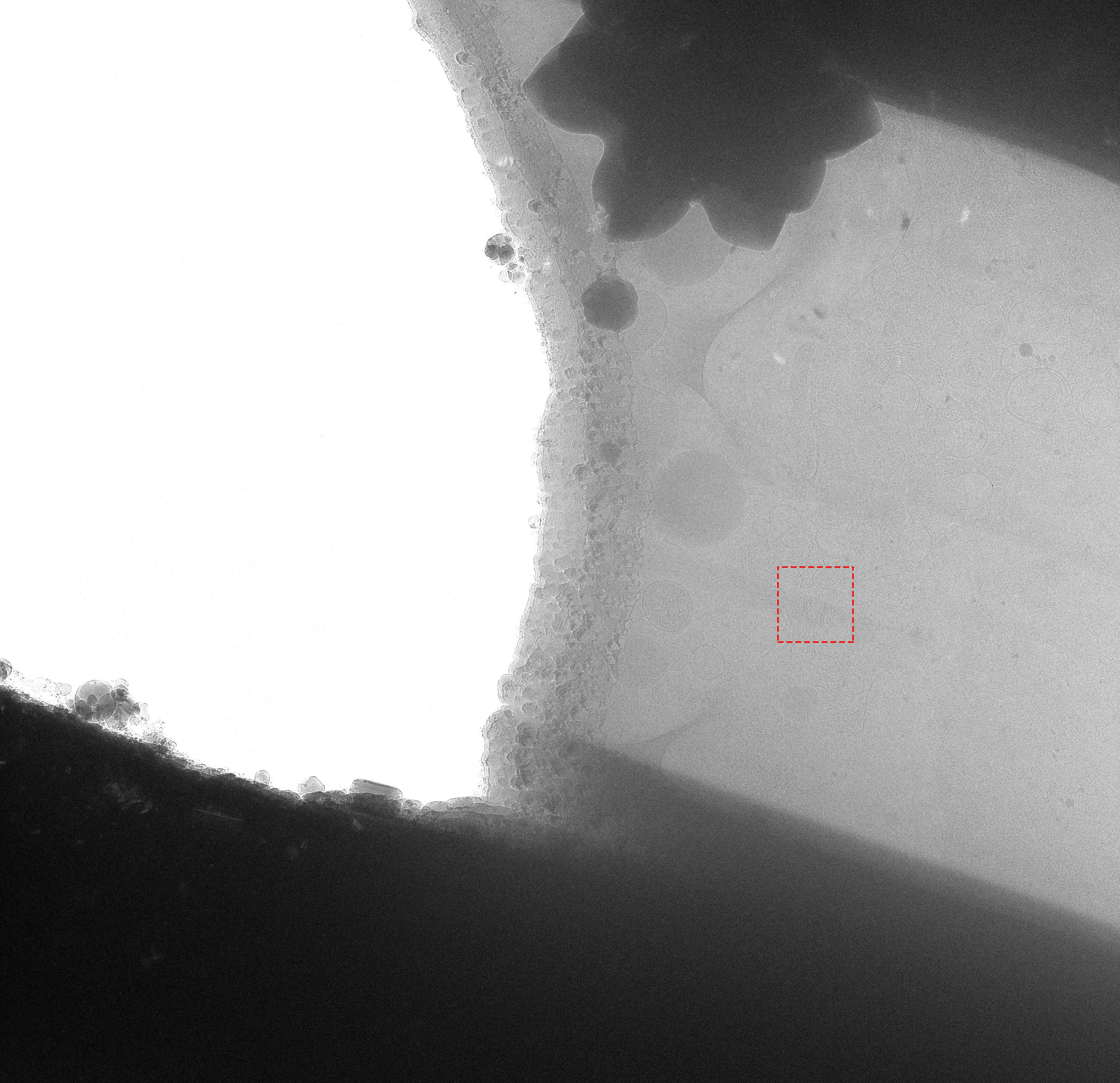

Supplement: Supplementary file 8 — Raw cryo-EM images of all the cryo-lamellae shown in Supplementary Fig. 1. The locations of centrioles are marked by dashed squares. [file 41592_2022_1748_MOESM8_ESM.zip › Supplementary_Data1/Lamella78_Location71.jpg]

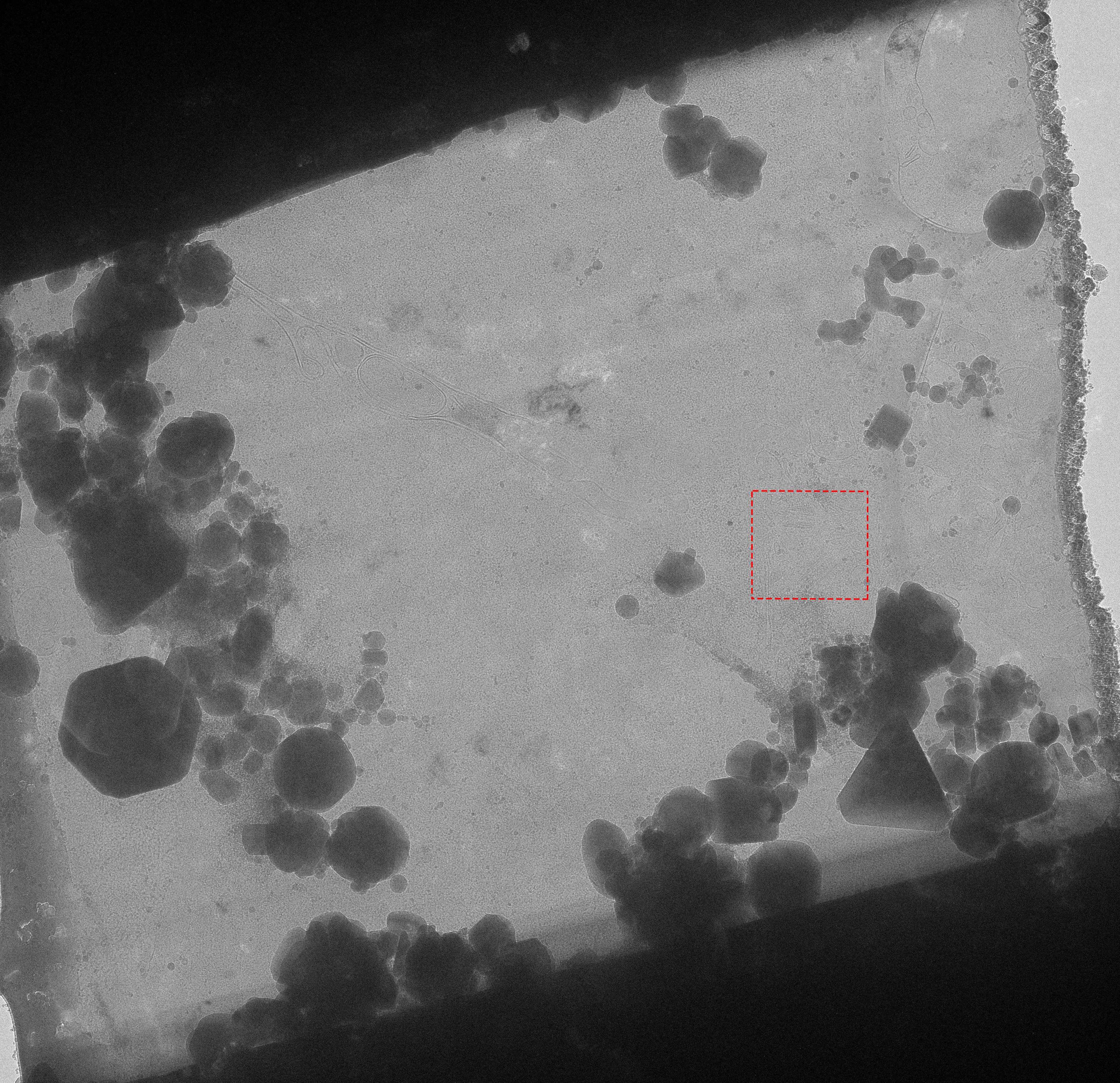

Supplement: Supplementary file 8 — Raw cryo-EM images of all the cryo-lamellae shown in Supplementary Fig. 1. The locations of centrioles are marked by dashed squares. [file 41592_2022_1748_MOESM8_ESM.zip › Supplementary_Data1/Lamella36_Location34.jpg]

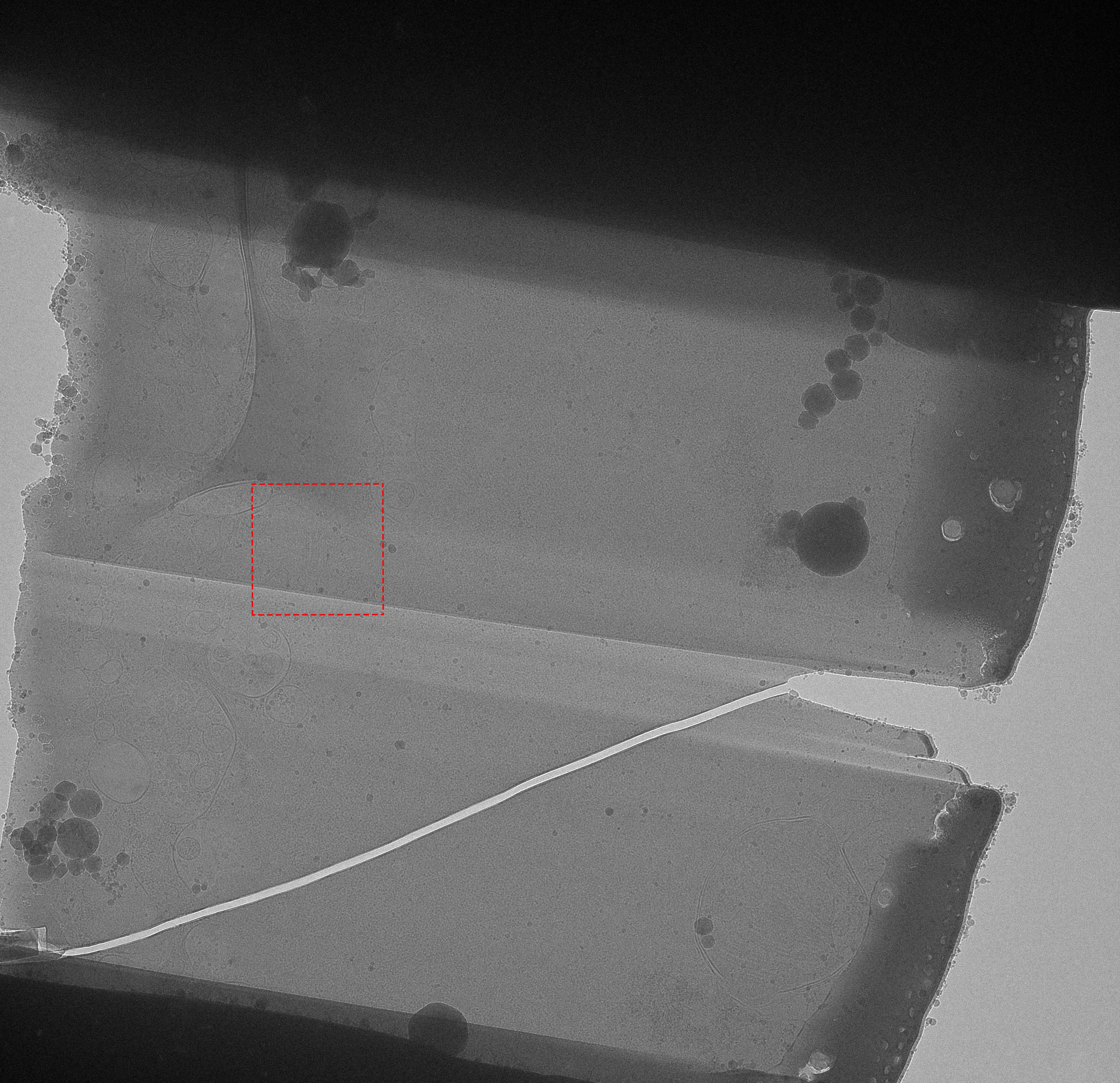

Supplement: Supplementary file 8 — Raw cryo-EM images of all the cryo-lamellae shown in Supplementary Fig. 1. The locations of centrioles are marked by dashed squares. [file 41592_2022_1748_MOESM8_ESM.zip › Supplementary_Data1/Lamella24_Location23.jpg]

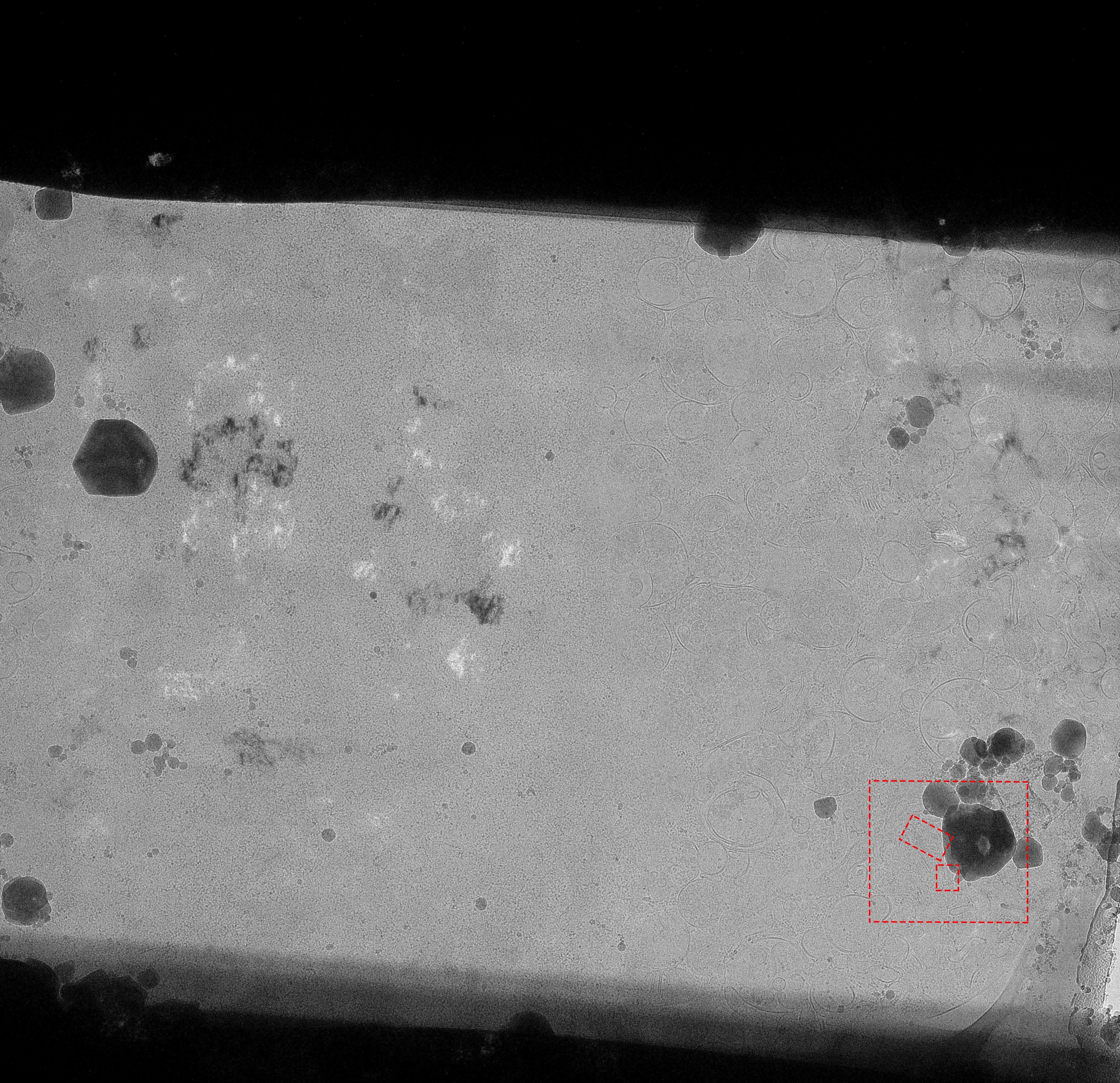

Supplement: Supplementary file 8 — Raw cryo-EM images of all the cryo-lamellae shown in Supplementary Fig. 1. The locations of centrioles are marked by dashed squares. [file 41592_2022_1748_MOESM8_ESM.zip › Supplementary_Data1/Lamella50_Location47.jpg]

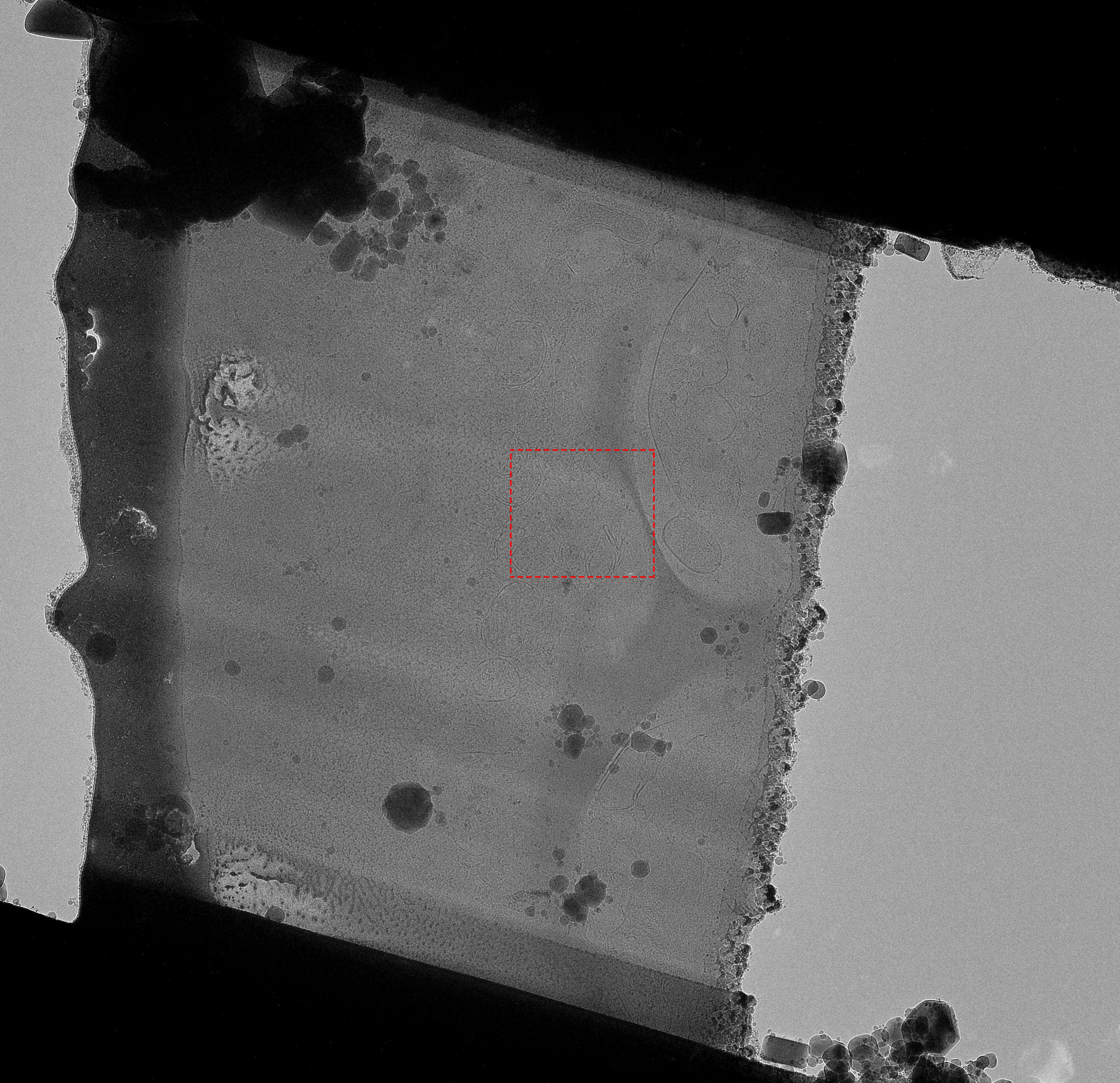

Supplement: Supplementary file 8 — Raw cryo-EM images of all the cryo-lamellae shown in Supplementary Fig. 1. The locations of centrioles are marked by dashed squares. [file 41592_2022_1748_MOESM8_ESM.zip › Supplementary_Data1/Lamella39_Location37.jpg]

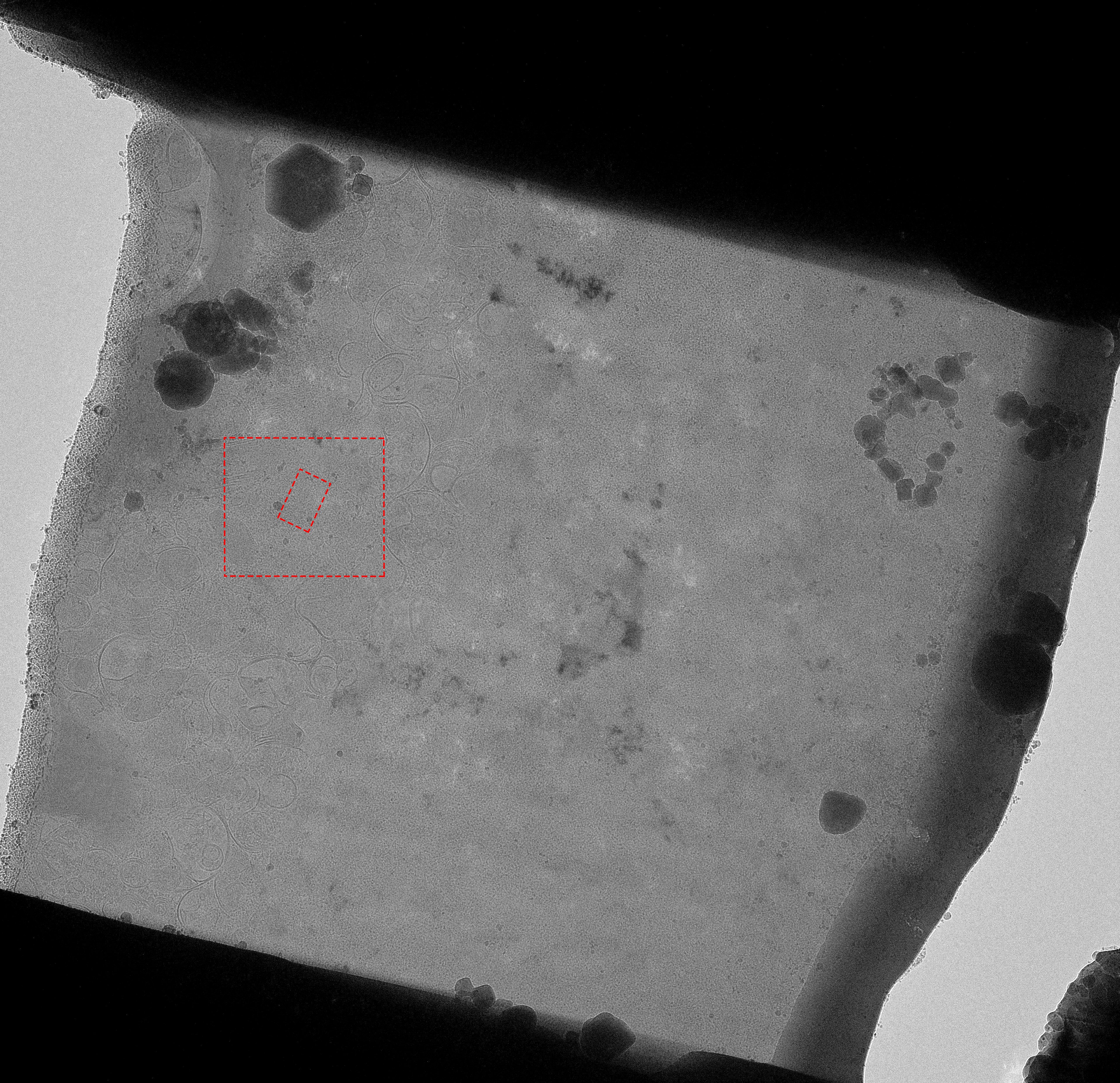

Supplement: Supplementary file 8 — Raw cryo-EM images of all the cryo-lamellae shown in Supplementary Fig. 1. The locations of centrioles are marked by dashed squares. [file 41592_2022_1748_MOESM8_ESM.zip › Supplementary_Data1/Lamella59_Location56.jpg]

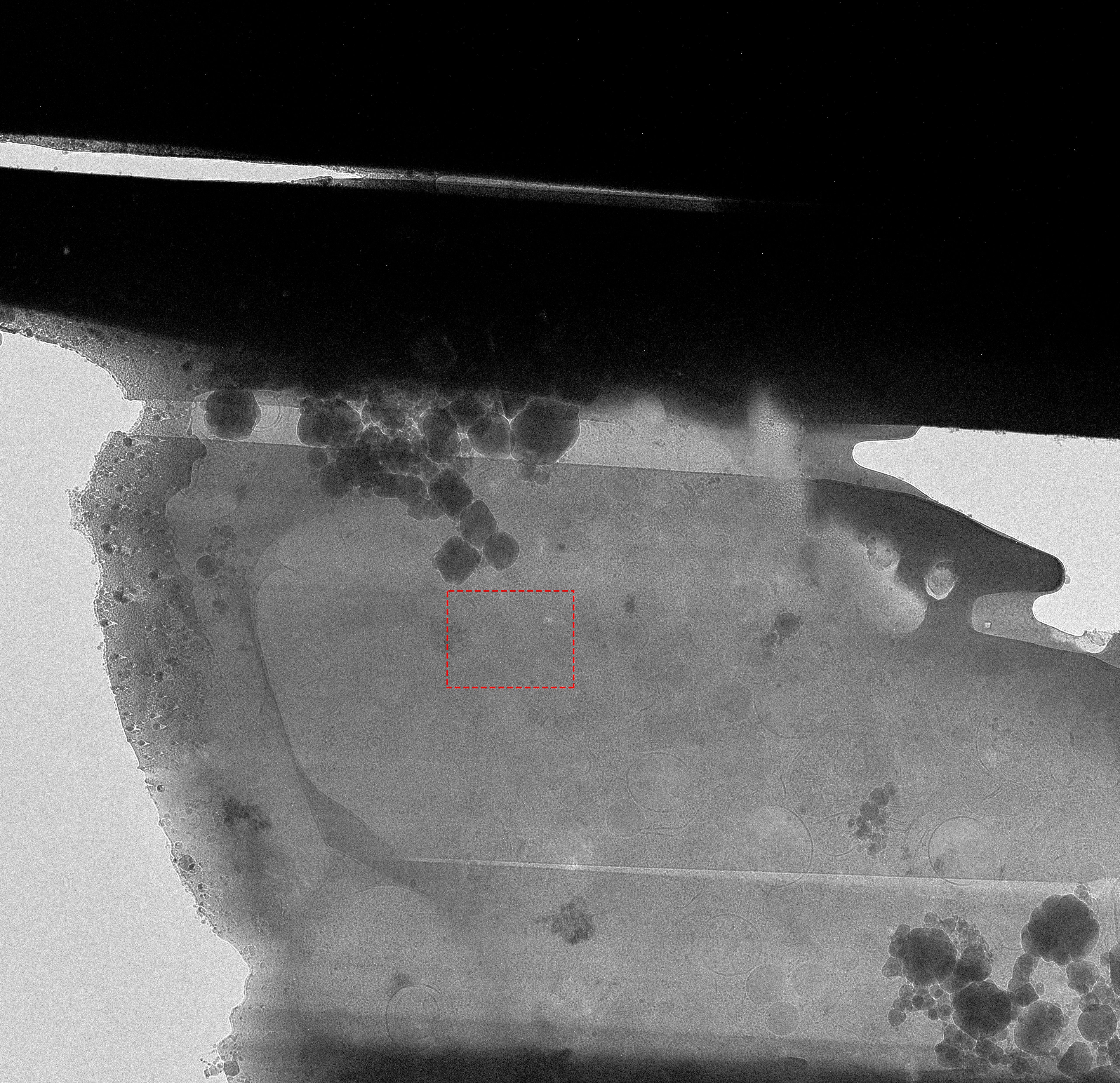

Supplement: Supplementary file 8 — Raw cryo-EM images of all the cryo-lamellae shown in Supplementary Fig. 1. The locations of centrioles are marked by dashed squares. [file 41592_2022_1748_MOESM8_ESM.zip › Supplementary_Data1/Lamella77_Location70.jpg]

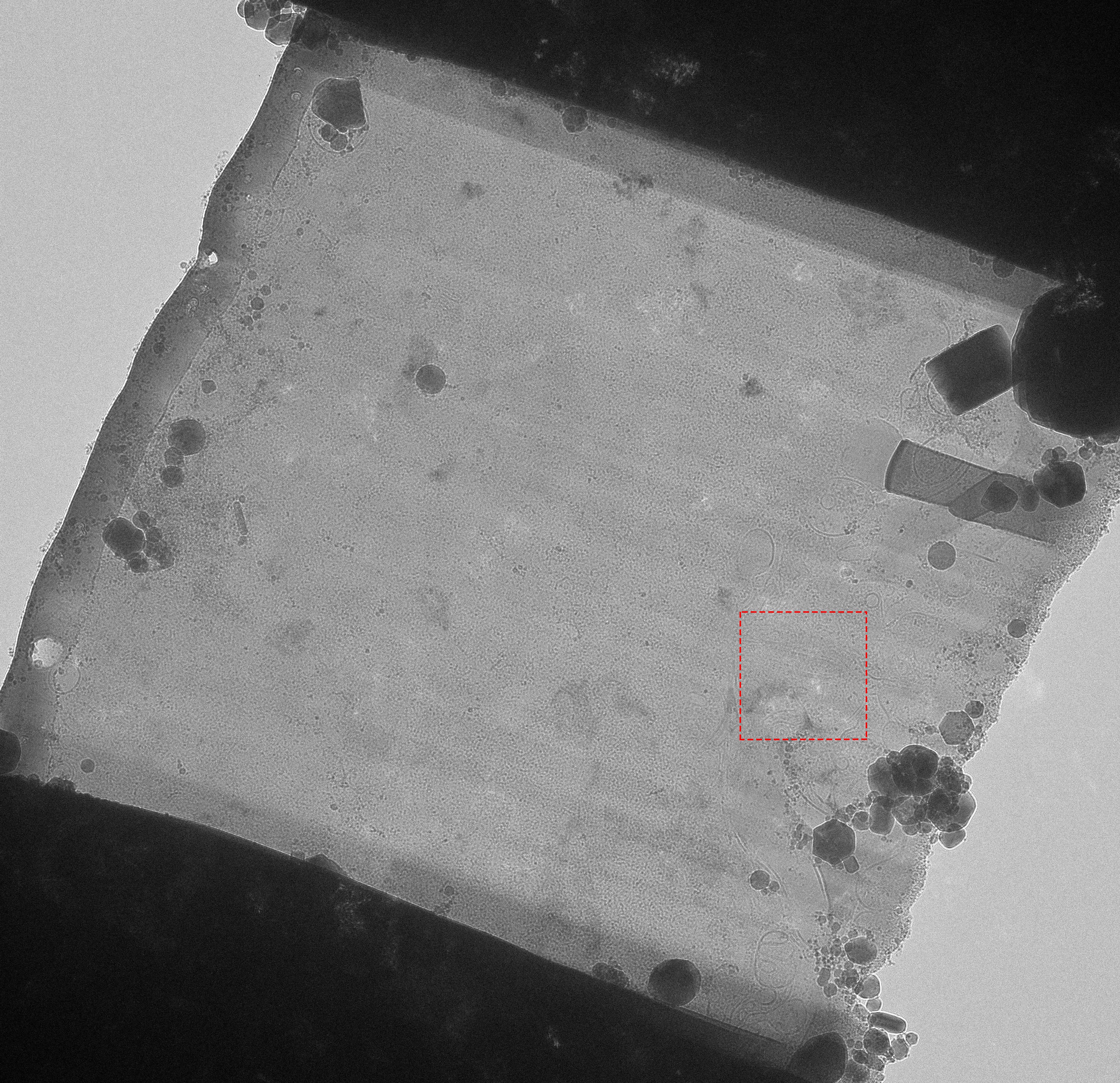

Supplement: Supplementary file 8 — Raw cryo-EM images of all the cryo-lamellae shown in Supplementary Fig. 1. The locations of centrioles are marked by dashed squares. [file 41592_2022_1748_MOESM8_ESM.zip › Supplementary_Data1/Lamella43_Location40.jpg]
